# Early risk assessment in paediatric and adult household contacts of confirmed tuberculosis cases by novel diagnostic tests (ERASE-TB): protocol for a prospective, non-interventional, longitudinal, multicountry cohort study

**DOI:** 10.1136/bmjopen-2022-060985

**Published:** 2022-07-19

**Authors:** Edson Tawanda Marambire, Denise Banze, Alfred Mfinanga, Junior Mutsvangwa, Theodora D Mbunda, Nyanda Elias Ntinginya, Khosa Celso, Gunilla Kallenius, Claire J Calderwood, Christof Geldmacher, Kathrin Held, Tejaswi Appalarowthu, Friedrich Rieß, Ursula Panzner, Norbert Heinrich, Katharina Kranzer, the ERASE-TB Consortium, Anna Shepherd

**Affiliations:** 1 Biomedical Research and Training Institute, Harare, Zimbabwe; 2 Instituto Nacional de Saúde, Marracuene, Mozambique; 3 National Institute for Medical Research- Mbeya Medical Research Centre, Mbeya, Tanzania; 4 Karolinska Institutet, Stockholm, Sweden; 5 Department of Clinical Research, Faculty of Infectious and Tropical Diseases, London School of Hygiene & Tropical Medicine, London, UK; 6 Division of Infectious Diseases and Tropical Medicine, University Hospital, LMU Munich, Munich, Germany; 7 German Center for Infection Research (DZIF), Partner site Munich, Munich, Germany

**Keywords:** Tuberculosis, Diagnostic microbiology, RESPIRATORY MEDICINE (see Thoracic Medicine)

## Abstract

**Introduction:**

The WHO End-TB Strategy calls for the development of novel diagnostics to detect tuberculosis (TB) earlier and more accurately. Better diagnostics, together with tools to predict disease progression, are critical for achieving WHO End-TB targets. The **E**arly **R**isk **A**ssessment in TB Contacts by new diagno**S**tic t**E**sts (ERASE-TB) study aims to evaluate novel diagnostics and testing algorithms for early TB diagnosis and accurate prediction of disease progression among household contacts (HHCs) exposed to confirmed index cases in Mozambique, Tanzania and Zimbabwe.

**Methods and analysis:**

A total of 2100 HHCs (aged ≥10 years) of adults with microbiologically-confirmed pulmonary TB will be recruited and followed up at 6-month intervals for 18–24 months. At each time point, a WHO symptom screen and digital chest radiograph (dCXR) will be performed, and blood and urine samples will be collected. Individuals screening positive (WHO symptom screen or dCXR) will be requested to provide sputum for Xpert MTB/Rif Ultra. At baseline, HHCs will also be screened for HIV, diabetes (HbA1c), chronic lung disease (spirometry), hypertension and anaemia. Study outcomes will be coprevalent TB (diagnosed at enrolment), incident TB (diagnosed during follow-up) or no TB at completion of follow-up. Novel diagnostics will be validated using fresh and biobanked samples with a nested case–control design. Cases are defined as HHCs diagnosed with TB (for early diagnosis) or with incident TB (for prediction of progression) and will be matched by age, sex and country to HHCs who remain healthy (controls). Statistical analyses will include assessment of diagnostic accuracy by constructing receiver operating curves and calculation of sensitivity and specificity.

**Ethics and dissemination:**

ERASE-TB has been approved by regulatory and ethical committees in each African country and by each partner organisation. Consent, with additional assent for participants <18 years, is voluntary. Attestation by impartial witnesses is sought in case of illiteracy. Confidentiality of participants is being maintained throughout. Study findings will be presented at scientific conferences and published in peer-reviewed international journals.

**Trial registration number:**

NCT04781257.Cite Now

**Strengths and limitations of this study**

- Recruitment of highly infectious index cases aimed at maximising the number of tuberculosis (TB) diagnoses in the household contact (HHCs) cohort.
- Sequencing of *Mycobacterium tuberculosis* isolates from both index cases and HHCs allows confirmation of household transmission and, thus, determination of timing of the transmission event; resulting in more precise estimates of new test sensitivity compared with population-based cohorts with unknown timing of infection.
- Large sample size across three southern African countries with high HIV prevalence; including adolescents will ensure that study findings are generalisable to the clinically relevant population at high risk of TB compared with studies focused on adults only.
- Despite the large cohort of HHCs, the number of diagnosed TB cases will be small, limiting the power of the study and subgroup analyses such as by age and HIV status.
- Geographically limited to sub-Saharan Africa; therefore, results may not be generalisable to other populations, including those with lower HIV prevalence such as in South-East Asia or the Americas.

## Introduction

Tuberculosis (TB) remains a leading global public health problem, with an estimated 10 million new cases and 1.5 million deaths globally in 2020.^1^ In 2014, the World Health Assembly approved the WHO End-TB Strategy, aiming for a 90% reduction in TB incidence and 95% reduction in TB deaths by 2035.^2^ However, in 2019, three million TB cases (‘the missing millions’) remained undiagnosed and untreated globally, resulting in potentially avoidable morbidity, mortality and onward transmission. The COVID-19 pandemic has resulted in a large decrease in the number of people newly diagnosed with TB and reported. This has increased the diagnostic gap by a further 1.3 million, resulting in an estimated 4.2 million undiagnosed TB cases in 2020.^3^ Also, for the first time in a decade, TB deaths have risen, from an estimated 1.4 million in 2019 to 1.5 million in 2020, as a result of reduced access to and provision of essential TB services, including diagnostics during the COVID-19 pandemic.

Without an efficacious and safe vaccine, early detection and containment are the main tools to interrupt transmission and successfully control TB. Similar to SARS-CoV2, asymptomatic spreading of *M.tuberculosis* and subclinical but infectious disease states are a major concern in the control of airborne infectious diseases.^4^ Early and accurate identification of persons with TB, combined with identification of those at risk of progression to TB and provision of targeted preventive treatment, are critical to reducing TB-associated morbidity and mortality, and preventing onward transmission.

Currently available diagnostics such as sputum microscopy, mycobacterial culture and nucleic acid amplification tests are based on direct pathogen detection, thus requiring a high mycobacterial load; they, therefore, predominately target advanced TB when onward transmission and significant lung damage has occurred.^5 6^ Furthermore, for many patients with minimal or no symptoms, expectoration of high-quality sputum specimens remains challenging, limiting the accuracy of sputum-based tests. The same holds true for young children and people living with HIV.

The **E**arly **R**isk **A**ssessment in TB Contacts by new diagno**S**tic t**E**sts (ERASE-TB) study aims to fill this diagnostic gap by evaluating new sputum and non-sputum-based TB diagnostics for early TB detection (before onward transmission occurs) as well as tools for more accurate prediction of TB progression to allow for targeted preventive therapy.

## Methods and analyses

### Study objectives

ERASE-TB’s primary objectives are (1) to determine the sensitivity and specificity of novel diagnostics to detect TB, in particular, asymptomatic or minimally symptomatic TB, (2) to evaluate novel diagnostics for detection of likely TB progression and (3) to enhance the performance of novel diagnostics by simulating testing algorithms coupled with individual risk estimates from a mathematical model. The secondary objectives are (1) to determine the TB prevalence among household contacts (HHCs) of infectious TB index cases (ICs) at baseline and during 18–24 months follow-up, (2) to establish a biorepository of cryopreserved specimens from HHCs for future development and validation of diagnostic tests and (3) to assess the association of selected chronic disease conditions and TB among HHCs.

### Study endpoints

The study’s primary endpoints are the presence or development of TB among HHCs with the following possible scenarios of (1) prevalent symptomatic TB at baseline, (2) incident TB during follow-up and (3) remained healthy until study completion. An endpoint review committee will review the data and case classification before finalisation.

Through the sequencing of *Mycobacterium tuberculosis* (*Mtb*) isolates, cases of coprevalent or incident TB will be classified either as secondary, infected by the source case—the timepoint of infection will be known; or as infected by another, unknown source of infection, with an unknown timepoint of infection.

### Recruitment sites

**Figure 1:**
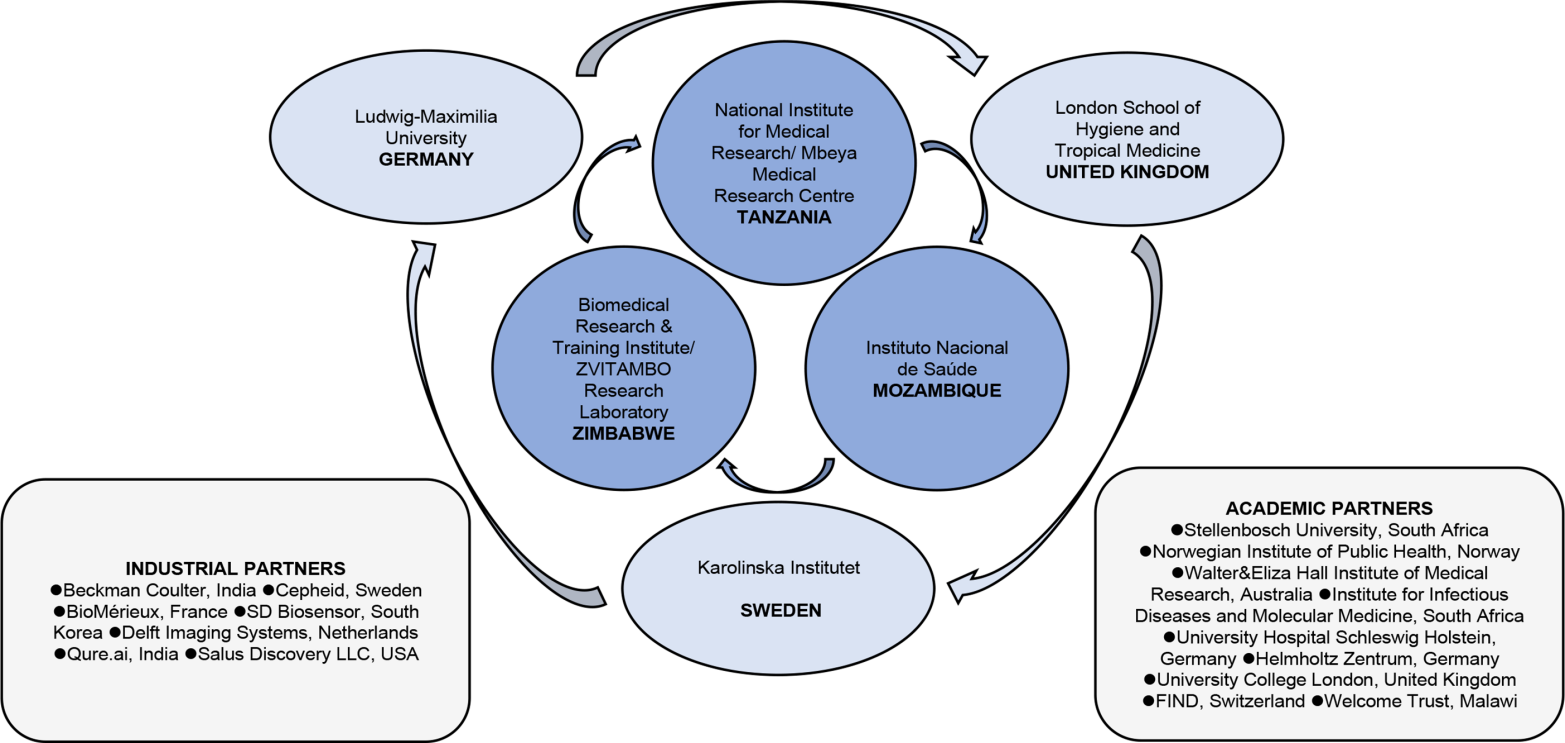
The ERASE-TB consortium. ERASE-TB, Early Risk Assessment in TB contactS by new diagnostic tEsts.

**Figure 2:**
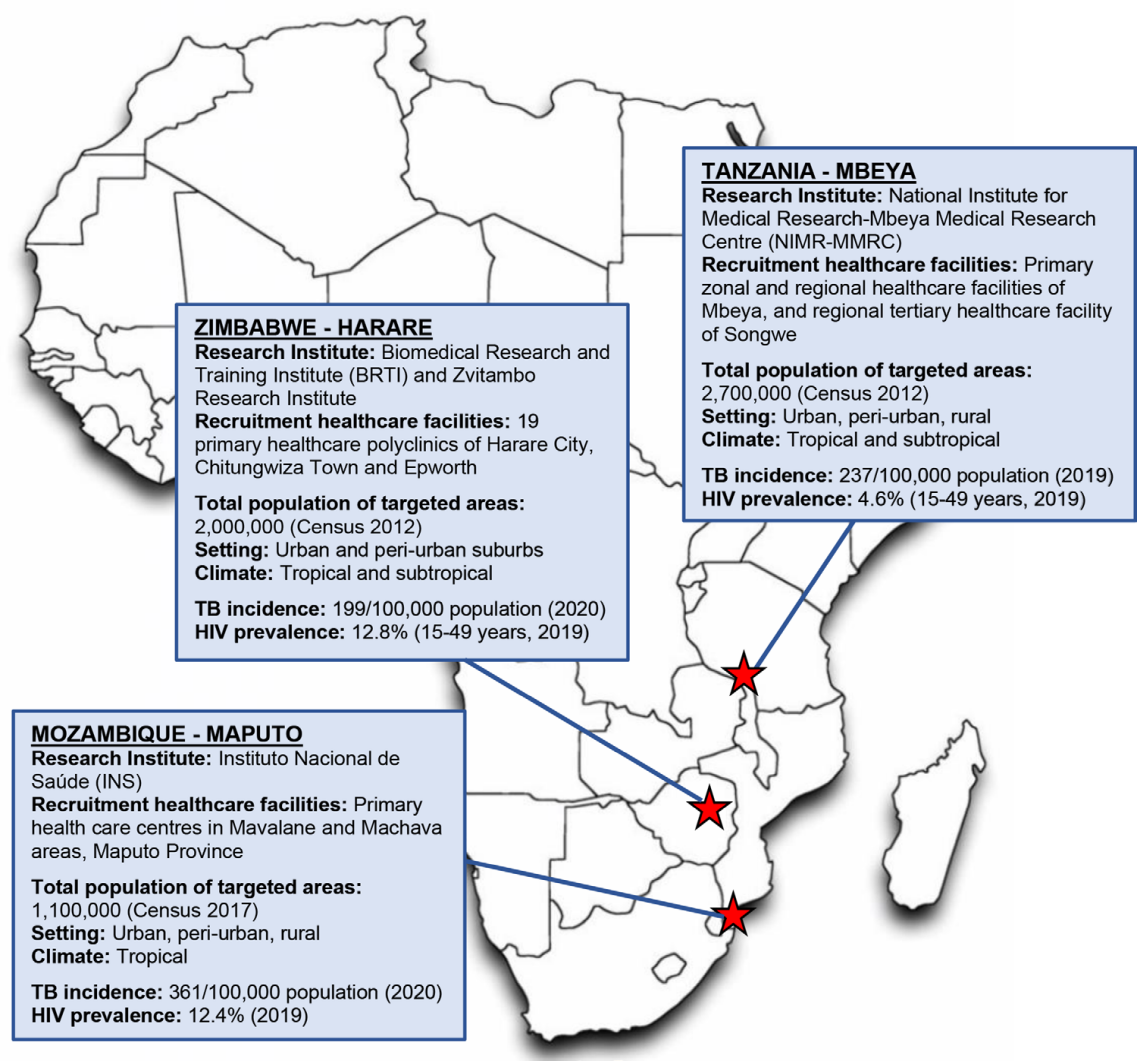
Location and characteristics of ERASE-TB study sites. The location of each study site is indicated by a red asterisk. Source data used within this figure are taken from the references.^7–15 40–42^ ERASE, Early Risk Assessment in TB contactS by new diagnostic tEsts; TB, tuberculosis.

Recruitment of ICs and HHCs at selected primary healthcare facilities and communities has commenced in Harare, Zimbabwe in March 2021, Maputo, Mozambique in August 2021, and Mbeya, Tanzania in September 2021. Partners of the ERASE-TB consortium are illustrated in figure 1. All three countries have a high TB incidence ranging from 100 to 499/100 000 population^1^ and HIV prevalence among adults aged 15 years and older of 5%–20%.^7^ The African research institutions have established collaborations with their respective National Tuberculosis Programs ensuring referral and appropriate follow-up of patients with TB. Figure 2 illustrates the geographic location of research institutions, healthcare facilities where recruitment is taking place, demographic characteristics of study populations and estimates on TB incidence and HIV prevalence.^8–15^

### Study design

**Figure 3:**
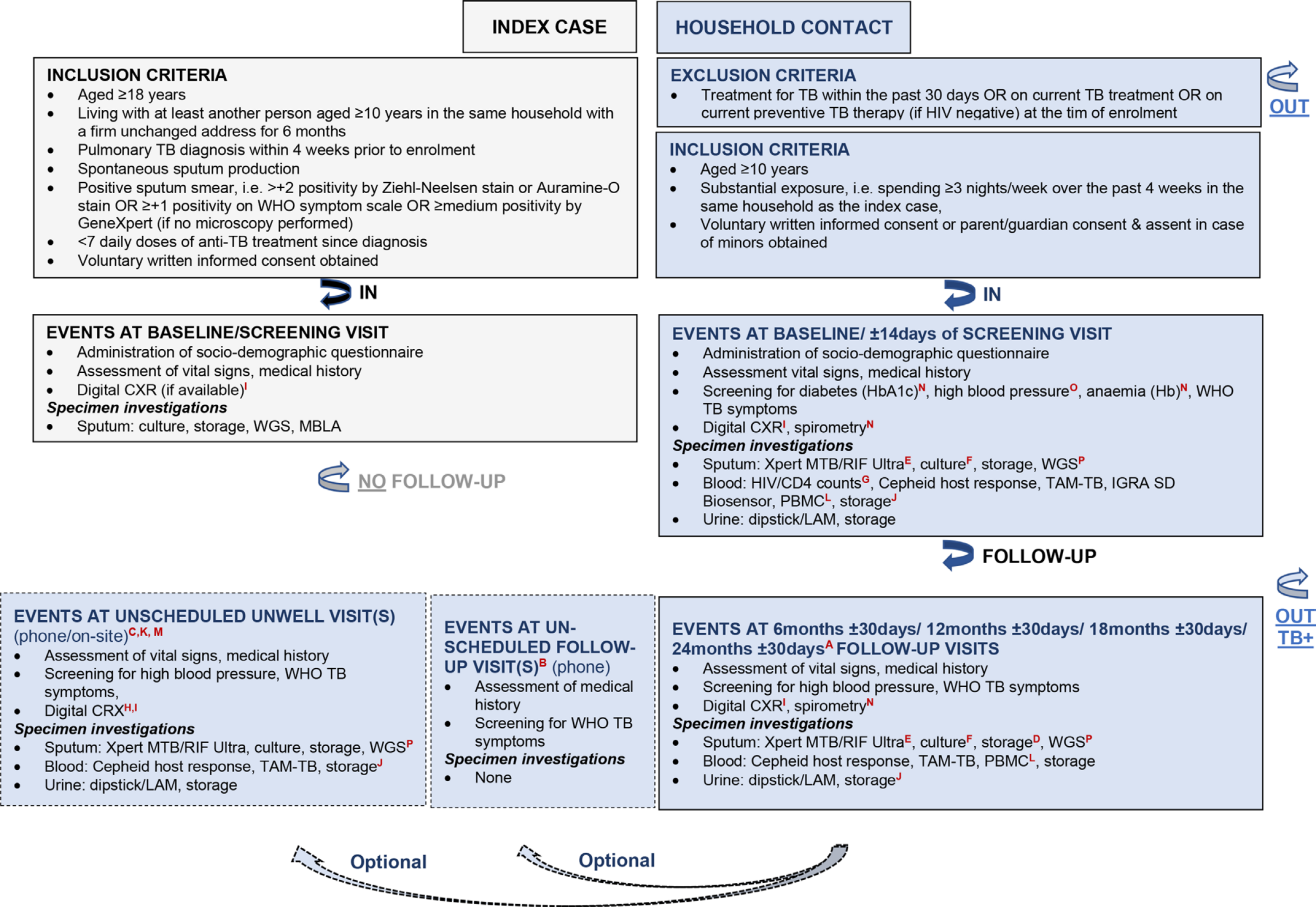
Eligibility criteria and schedules of events for index cases and household contacts. A=depending on the time point of study enrolment and consequently on the duration available for follow-up, that is, 18 or 24 months, the follow-up visit at 24 months±30 days may be conditional; B=the follow-up visit by phone may be conducted after the last scheduled follow-up visit at 18 months±30 days or 24 months ±30 days to assess whether symptoms suggestive of TB have occurred, TB diagnosis has been made or anti-TB treatment has been initiated; C=unwell visits by phone or on-site may be conducted between scheduled follow-up visits if a participant presents at a recruitment healthcare facility with signs and symptoms suggestive of TB; D=coached spontaneous or induced sputum collection for storage at scheduled follow-up visit at 18 months±30 days or 24 months ±30 days, and for repetition of HIV testing if tested negative at baseline; E=coached spontaneous or induced sputum collection on the decision of the investigating team for testing by Xpert MTB/RIF Ultra if participant presents with signs and symptoms suggestive of TB; F=coached spontaneous or induced sputum collection in case of Xpert MTB/RIF Ultra positivity or strong clinical suspicion of TB for repetition of the Xpert MTB/RIF Ultra; G=in case of HIV positivity to be followed by the assessment of CD4 counts; H=CXR to be conducted at an unscheduled on-site unwell visit on the decision of the investigating team depending on the nature of symptoms reported, and the time elapsed since the last CXR including its findings; I=not to be conducted among pregnant women; J=stored venous blood includes 6 mL EDTA blood for whole blood and plasma, 4 mL serum and 2.5 mL PAXgene blood, all samples will be deep frozen for retrospective testing using new diagnostics as described in text; K=in case the evaluation of symptoms of a participant unable to present at a recruitment healthcare facility is required an unscheduled on-site or home visit will be arranged by phone, the resolution of symptoms can alternatively be addressed by phone; L=collection of PBMC at baseline and follow-up visit at 6 months±30 days is optional, thus will not be performed at each participating site and for each participant; M=in case the evaluation of symptoms of a participant unable to present at a recruitment healthcare facility is required or doubtful if required an unscheduled unwell visit by phone will be arranged, the resolution of symptoms can alternatively be addressed by phone; N=spirometry and/or diabetes (HbA1c) will be performed at scheduled follow-up visits at 6 months±30 days, 12 months ±30 days and 18 or 24 months±30 days if required or not performed at baseline, anaemia (Hb) will be performed at baseline and scheduled follow-up visits at 6 months±30 days, 12 months ±30 days and 18 or 24 months±30 days if possible; O=blood pressure measurement will be performed at baseline and scheduled follow-up visits at 6 months±30 days, 12 months ±30 days and 18 or 24 months±30 days; P=WGS to be performed once *Mtb* infection is confirmed and an isolate could be recovered. CD4, cluster of differentiation 4; CXR, chest radiograph; Hb, haemoglobin; HbA1c, glycated haemoglobin; IGRA, interferon gamma release assay; LAM, lioparabinomannan; MTB, *Mycobacterium tuberculosis*; MBLA, molecular bacterial load assay; PBMC, peripheral blood mononuclear cell; RIF, rifampicin; TAM-TB, T- cell activation marker tuberculosis; TB, tuberculosis; WGS, whole genome sequencing.

**Figure 4:**
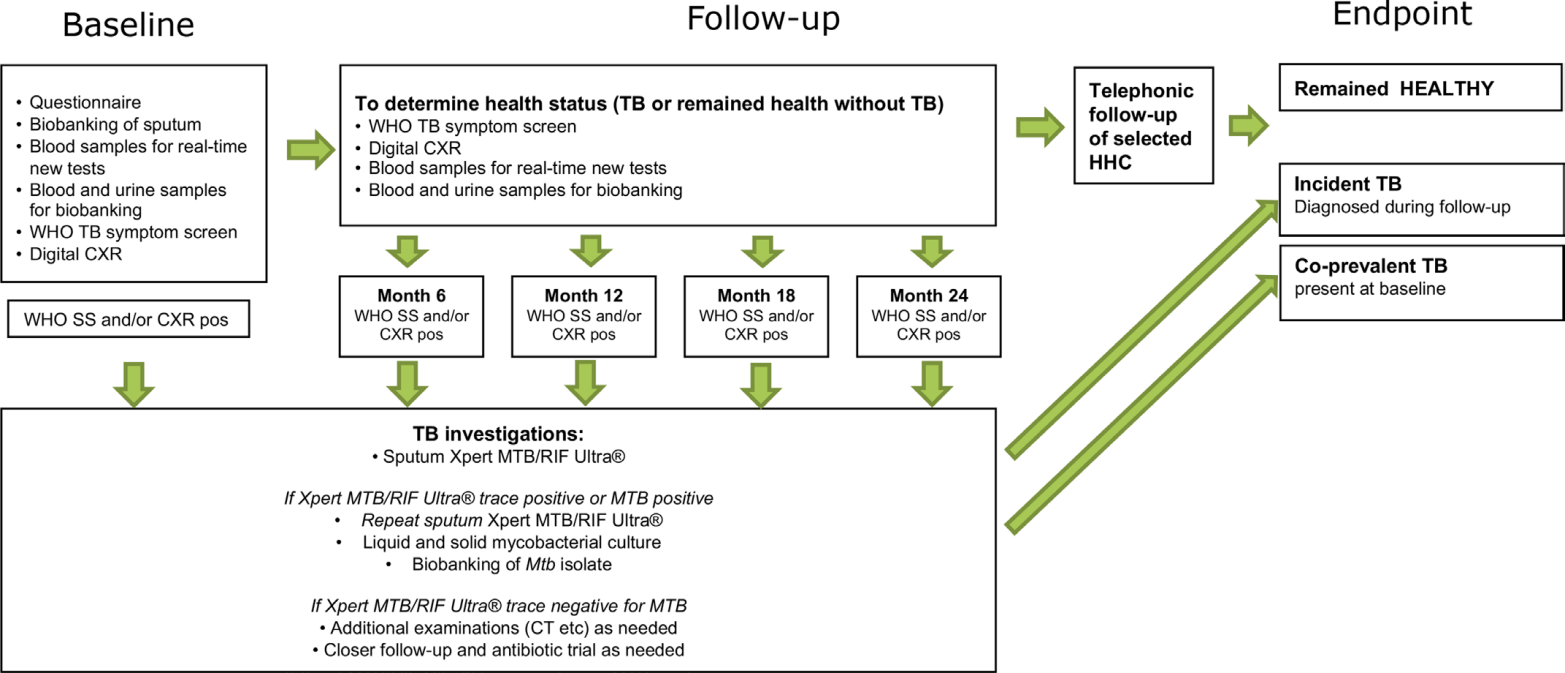
Study design. CXR, chest radiograph; FU, follow-up; HHC, household contact; IC, index case; MTB, *Mycobacterium tuberculosis*; pos=positive; RIF, rifampicin; SS, symptom score; TB, tuberculosis.

ERASE-TB is a non-interventional, longitudinal, prospective cohort study among HHCs aged ≥10 years exposed to highly infectious pulmonary TB ICs aged ≥18 years. Eligibility criteria are detailed in figure 3 and the study design is shown in figure 4. TB ICs are eligible if the bacterial load in their sputum is at least at the ‘medium’ level according to Xpert MTB/RIF or Xpert MTB/RIF Ultra, and they have received less than seven daily doses of anti-TB treatment before enrolment. This maximises the likelihood of culturing and storing *Mtb* isolates. The total study duration will be 36 months. This includes 12-month enrolment of ICs and HHCs, and 18-month to 24-month follow-ups of HHCs. Follow-up ends when a HHCs withdraws from the study, is lost to follow-up, dies or is diagnosed with TB and referred for treatment. Scheduled or unscheduled unwell visits can be conducted physically and/or telephonically in case of abnormal finding, for example, by abnormal digital chest radiograph (dCXR), or when a participant feels unwell in between scheduled follow-up visits.

### Procedures

#### TB index cases

Following informed consent obtained, a questionnaire is administered to collect sociodemographic information, TB risk factors and the medical history of TB, HIV and other diseases. Two spontaneous sputum samples are obtained, of which one is for mycobacterial culturing and one for storage for performing retrospectively Molecular Bacterial Load Assay to quantify viable *Mtb* by 16s rRNA^6^; an alternative means to quantify expectorated bacterial load for an estimate of infectiousness. Both liquid and solid mycobacterial cultures are performed on decontaminated sputum samples, with all *Mtb* isolates stored at −80° for future DNA extraction and whole genome sequencing. A questionnaire on symptom duration and TB risk factors is also administered.

#### Household key informant

At baseline, a household key informant (either the TB IC or one of the HHCs) is identified and asked to answer questions of a household questionnaire that collects socioeconomic elements like structure of the house or flat, income and household assets and covariates possibly associated with risk of TB infection, for example, windows/air exchange, presence of comorbid conditions and risk factors like the source of cooking energy and properties of the household kitchen.

#### Household contacts

Informed consent is obtained from all eligible adult HHCs. For HHC <18 years of age, the guardian is asked to provide informed consent, with assent also sought from children dependent on local guidance. At baseline, a questionnaire is administered collecting information on socioeconomic and demographic characteristics, previous medical history of TB, HIV and other diseases, exposure risk factors, smoking and alcohol history. The physical examination includes height, weight, mid-upper arm circumference and blood pressure measurement. In addition, all HHCs are offered free HIV testing according to the National Guidelines. All people with confirmed HIV infection will have CD4 counts performed and be referred for TB preventive therapy. Those not yet on antiretroviral therapy (ART) and those who interrupted ART are referred for ART at local services.

Point of care HbA1c (A1cCare, SD Biosensor, Gyeonggi-do, Republic of Korea) and haemoglobin (Hemocue 301+, Hemocue, Angelholm, Sweden) tests and spirometry (including prebronchodilation and postbronchodilation with inhaled salbutamol) are performed at baseline or the 6-month visit. HHCs who did not take up HIV testing or other screening at baseline are offered these tests at each study visit. Any HHCs with test results requiring treatment or further investigations are referred for respective services.

HHCs are screened for TB using the WHO symptom screening questionnaire and a dCXR, reviewed by a clinical officer. dCXRs are not performed in pregnant HHCs. HHCs with a positive WHO symptom screening and/or abnormal dCXR are asked to provide sputum samples for TB investigations, that is, for GeneXpert and mycobacterial culture. Those with negative symptom screen and normal dCXR are asked to provide a spontaneous sputum sample for storage (with sputum induction performed if required).

At baseline, urine, serum, plasma, whole blood (native, and with RNA preservation in PAXgene tubes (BD Biosciences, New Jersey)) are stored. A finger-prick sample is taken and investigated using the Xpert TB Host Response RUO Prototype cartridge (Cepheid, Sunnyvale, California). T-cell Activation Marker Tuberculosis (TAM-TB) assay and Interferon Gamma Release Assay (IGRA; STANDARDTM F TB-Feron FIA (IFN-gamma; SD Biosensor, Republic of Korea) are performed on fresh venous blood. In Tanzania and Mozambique, storage of peripheral blood mononuclear cells for later characterisation of the TB-specific immune response is also performed.

Procedures for follow-up and unwell visits are similar to those at baseline. Measurement of HIV status, haemoglobin (HB), HbA1c, spirometry, CD4 count and IGRA testing are not performed at follow-up visits, unless not done previously. At the last scheduled visit, all HHCs not known to have HIV are reoffered HIV testing and a spontaneous or induced sputum sample is stored for all participants.

#### HHCs screening positive for TB symptoms and/or with a dCXR suggestive of TB

HHCs screening positive for symptoms and/or those with dCXRs suggestive of TB are asked for a sputum sample, which is investigated using Xpert MTB/RIF Ultra (Cepheid). If this sample is positive for *Mtb* (including a trace result), a minimum of two additional sputum samples are investigated, following decontamination, with Xpert MTB/RIF Ultra, solid and liquid culture. Isolates stored from these cultures will be sequenced for matching with the IC isolates in order to verify intrahousehold transmission. Sputum induction is performed for those unable to provide a spontaneous sputum sample. HHCs with microbiologically confirmed TB are referred for TB treatment to the National TB Programme.

### Patient and public involvement

The ERASE-TB study sites have established Community Advisory Boards, which are voices of communities, people affected and study participants, providing a strategic link between the communities and the study team. Community Advisory Boards meet regularly and provide feedback on design, procedures and conduct of the study. They will also be closely involved in the dissemination of study results. In addition to the Community Advisory Boards, each study site conducts community engagement activities focused on young people with the aim to foster interst in science and research, specifically in the field of respiratory diseases/illness. This includes close partnership with schools and universities. Furthermore, planned qualitative research will specifically aim to understand the perceptions of HHCs with regards to TB diagnostics and screening.

### Sample size

An estimated 800 to 900 TB-confirmed ICs are required for the subsequent enrolment of an anticipated 2100 HHCs, that is, 700 HHCs per country. Loss to follow-up of HHCs is estimated to be 10%. A total of 64 HHCs (3%) are estimated to be diagnosed with TB during the study period, based on previous active case finding studies among HHCs.^16^ Validation for subclinical and early TB will include incident (n=49) and coprevalent TB cases (n=15). Validation for detection of incipient *Mtb* infection will include samples of participants with incident TB (n=49) matched 1:4 to samples of participants without TB (n=196). For tests diagnosing incipient *Mtb* infection sensitivities of 73% and 82% would be detected with a precision of 59%–85% and 68%–91%, respectively. For specificities of 92% and 94%, the CIs would be 87% to 95% and 90% to 97%.

### Novel test candidates

A range of novel test candidates targeted at pathogen detection or identification of host responses to *Mtb* are being applied, either in real-time (for all participants) or retrospectively (in a case–control design). While a number of novel test candidates have been prespecified, the ERASE-TB biobanking processes allow for addition of further candidate tests to be evaluated on stored samples as they become available.

DCXRs offer good sensitivity for diagnosis of pulmonary TB. However, high interinvestigator and intrainvestigator variability, and lack of trained interpreters presents a barrier to implementation in many high-TB burden settings. Computer-aided interpretation systems, such as CAD4TB (Delft Imaging, Hertogenbosch, Netherlands) and qXR (Qure.ai, India), may increase image-reading capacity, with good performance, and serve, therefore, as a systematic screening tool to identify individuals in need of confirmatory TB tests.^17 18^

Xpert MTB/RIF Ultra is a nucleic acid amplification test for *Mtb* with a lower limit of detection compared with the previous Xpert MTB/RIF generation, and, therefore, conferring higher sensitivity in paucibacillary specimens. This, however, comes at the expense of specificity, particularly in high TB incidence settings, resulting in ‘false positives’.^19^ WHO guidelines recommend Xpert MTB/RIF Ultra for TB diagnosis among adults and children acknowledging that further evaluation, particularly of the role of Xpert MTB/RIF Ultra for TB screening, is needed.^20 21^

FLOW-TB is an advanced ELISA for the detection of *Mtb* lipoarabinomannan (a mycobacterial cell wall component) in urinary specimens with results available within 65 min.^22^

The TAM-TB detects Mtb-specific CD4 T-cells through in vitro antigen stimulation with *Mtb*-derived peptides, that is, from ESAT-6 to CFP-10, followed by flow cytometry. TAM-TB discriminated latent *Mtb* infection from TB in freshly collected blood with 83% sensitivity and 96%–98% specificity in previous studies. Furthermore, TAM-TB may detect early TB disease progression up to 9 months prior to the identification of *Mtb* in sputum.^23–25^

Multiple transcriptomic signatures, capturing the host response to TB, have been described as promising candidate tests for earlier TB diagnosis (up to 2 years before microbiological diagnosis). An individual patient data meta-analysis suggested equivalent performance of eight signatures, with 25%–40% sensitivity and 92%–95% specificity, 0–24 months before TB diagnosis. Diagnostic accuracy of each signature improved as the interval between testing and microbiological TB diagnosis shortened.^26^ Several signatures have been developed into PCR-based assays to facilitate real-time implementation: the recent The Correlate of Risk Targeted Intervention Study (CORTIS) reported sensitivity of 48% and specificity of 75% for incipient TB for the RISK-11 transcriptomic signature of TB risk.^27^ Cepheid have developed a three-transcript TB score into a fully automated in-cartridge PCR assay performed on finger-prick blood using the Xpert platform (Xpert TB Host Response RUO Prototype cartridge). This cartridge will be evaluated using freshly collected specimens in ERASE-TB; storage of RNA-stabilised blood samples also allows for retrospective evaluation of additional transcriptomic signatures in our cohort.^28 29^

An alternative approach to capture the host response to TB is through protein-based biomarker signatures. Candidate tests in this category include a serum-based or plasma-based multiplex assay assessing 13 protein biomarkers (C reactive protein, procalcitonin,^30^ sTREM-1 (soluble triggering receptor expressed on myeloid cells-1),^31 32^ angiopoietin-2,^33 34^ IL-6,^35^ TRAIL (TNF-related apoptosis-inducing ligand)^36^ and IP-10^35^) that is being developed by the London School of Hygiene and Tropical Medicine; in addition, a seven biomarker signature is under development as a point-of-care test for TB diagnosis, with 94% sensitivity and 73% specificity detected in previous work.^37^

### Statistical analyses

Baseline characteristics and analytical data will be summarised using descriptive statistics inclusive of mean, median, range, SD and absolute as well as relative frequencies depending on the nature of data. A logistic regression model will be used to identify characteristics of TB among ICs, households and HHCs that are predictive of incident TB. From the study database, we will simulate algorithms of different tests to obtain the testing combination with the best accuracy. We will couple tests with a mathematical model that quantifies the risk of infection and/or disease to enhance predictive performance. The reporting of the development of the prediction model will follow the Transparent Reporting of a multivariable prediction model for Individual Prognosis Or Diagnosis Initiative.^38^

The validation of novel diagnostic tests for detecting TB will be analysed as a 1:4 matched nested case–control study with HHCs diagnosed with TB at baseline and during follow-up serving as cases, and HHCs who do not develop TB during follow-up as controls; controls will be matched for site, age, sex, HIV status and other risk factors for developing TB. Sensitivity and specificity of novel tests will be determined using pre-existing positive/negative cut-offs where these exist^39^; and receiver operating curves (ROC) constructed with area under the ROC curve calculated. For tests aiming to identify individuals at high risk of TB in the future, only HHCs who are diagnosed with TB during follow-up will serve as cases (ie, those diagnosed with TB at baseline will be excluded). Stored samples from all timepoints will be retrieved and diagnostic accuracy (ie, sensitivity and specificity) of the novel test determined at different timepoints before TB diagnosis. The decision of assigning the ‘active TB’ endpoints to participants will be blinded from the new test results to avoid inclusion bias.

### Data management

All source data will be kept confidential in secured locations with restricted access by authorised personnel only inclusive of monitors, auditors and reviewers of ethical and regulatory committees in line with applicable data privacy regulations. Each participant is asked to consent to this handling of the data and is assigned a pseudonymous identification number that is used throughout the study on all source data.

Accurate documentation of paper-based and electronic source data, for example, original records and certified copies of original records, progress notes, screening logs and recorded data from automated instruments, will be maintained. The pseudonymised clinical data captured on paper-based Case Report Forms will be entered at the sites into a database using the web-based Clinical Data Management System of OpenClinica (OpenClinica LLC, Waltham, Massachusetts). The study-specific database has been built, maintained and hosted by the LMU Klinikum on a centralised secure server. Data modifications and necessary corrections performed in the database also within the context of double data entry will be documented and tracked in audit trails. Data quality and plausibility are assured by a series of preprogrammed edit and range checks in OpenClinica. Further validation checks are programmed in Stata (Statacorp, College Station, Texas) with extracts of the database and electronically received data, for example, spirometry, dCXR and laboratory, will be integrated into analyses of datasets.

### Monitoring

Assigned study monitors will visit the sites at regular intervals physically and/or virtually in addition to frequent day-to-day communication. Close follow-up on all study-related aspects will be performed to ascertain compliance with standards of Good Clinical Practice, the Declaration of Helsinki and other local and national regulatory guidelines inclusive of guidelines for infection prevention and control of airborne-transmitted diseases, for example, social distancing in well-ventilated spaces, and wearing of personal protective equipment. In particular, monitors that support designated study personnel are responsible to verify (1) adequacy of the study personnels’ qualifications and facilities, (2) accuracy of informed consent procedures and patient eligibility, (3) adherence to the study protocol, (4) protection of rights and well-being of participants, (5) adherence to infection prevention and control measures, (6) accuracy and completeness of study documents and other study-related records and (7) maintenance of source documents.

### Ethics and dissemination

The study protocol and informed consent/assent documents have been approved by regulatory and ethical committees of the participating institutions (Medical Research Council in Zimbabwe (MRCZ/A/2618), the National Health Research Ethics Committee in Tanzania (TMDA-WEB0021/CTR/0004/03), the National Bioethics Committee for Health in Mozambique (541/CNBS/21), and the ethical committees of London School of Hygiene & Tropical Medicine, United Kingdom (22 522–2) and the medical faculty of the Ludwig-Maximilians-Universität München, Germany (20–0771)).

Adult ICs and HHCs are asked for written informed consent prior to their participation. Underage HHCs are asked for assent in addition to obtaining the consent of their legal guardians/parents; with ages for assent depending on local guidance. In case of illiteracy, the participant is asked to give its consent by fingerprint while an adult impartial, literate witness present during the entire consent procedure signs the consent on behalf. All participants have the right to withdraw from the study at any time. Findings derived from ERASE-TB will be presented at scientific conferences and published in peer-reviewed international journals.

### Current study status

The recruitment of ICs and HHCs is in progress in Zimbabwe, Mozambique and Tanzania since March, August and September 2021, respectively. The follow-up of HHCs is anticipated to be completed in March, August and September 2023 in Zimbabwe, Mozambique and Tanzania, respectively; laboratory analyses are estimated to be performed by December 2024.

## Supplementary Material

Reviewer comments

Author's manuscript
