# Supplementary material for: Early risk assessment in paediatric and adult household contacts of confirmed tuberculosis cases by novel diagnostic tests (ERASE-TB): protocol for a prospective, non-interventional, longitudinal, multicountry cohort study
Source: BMJ Open. 2022 Jul 19;12(7):e060985. doi: 10.1136/bmjopen-2022-060985 (PMC9301805; doi:10.1136/bmjopen-2022-060985)
Supplement: Author's manuscript [file bmjopen-2022-060985.draft_revisions.pdf]

BMJ Open is committed to open peer review. As part of this commitment we make the peer review history of every article we publish publicly available.

When an article is published we post the peer reviewers' comments and the authors' responses online. We also post the versions of the paper that were used during peer review. These are the versions that the peer review comments apply to.

The versions of the paper that follow are the versions that were submitted during the peer review process. They are not the versions of record or the final published versions. They should not be cited or distributed as the published version of this manuscript.

BMJ Open is an open access journal and the full, final, typeset and author-corrected version of record of the manuscript is available on our site with no access controls, subscription charges or pay-per-view fees (http://bmjopen.bmj.com).

If you have any questions on BMJ Open's open peer review process please email info.bmjopen@bmj.com

BMJ Open

Early risk assessment in pediatric and adult household contacts of confirmed tuberculosis cases by novel diagnostic tests (ERASE-TB): protocol for a prospective, non-interventional, longitudinal, multi-country cohort study

Journal:	*BMJ Open*
Manuscript ID	bmjopen-2022-060985
Article Type:	Protocol
Date Submitted by the Author:	11-Jan-2022

Complete List of Authors:	Marambire, Edson; Biomedical Research and Training Institute,<br>Banze, Denise; Instituto Nacional de Saúde<br>Mfinanga, Alfred; Mbeya Medical Research Centre<br>Mutsvangwa, Junior; Biomedical Research and Training Institute<br>Mbunda, Theodora ; NIMR-Mbeya Medical Research Programme<br>Ntinginya, Nyanda; 6. National Institute of Medical Research-Mbeya Medical Research Centre<br>Celso, Khosa; Instituto Nacional de Saúde<br>Kallenius, Gunilla; Karolinska Institutet<br>Calderwood, Claire J.; London School of Hygiene and Tropical Medicine Faculty of Infectious and Tropical Diseases<br>Geldmacher, Christof; University Hospital, Division of Infectious Diseases and Tropical Medicine, LMU Munich ; German Center for Infection Research, Partner site Munich<br>Held, Kathrin; University Hospital, Division of Infectious and Tropical Medicine, LMU Munich ; German Center for Infection Research, Partner site Munich<br>Appalarowthu, Tejaswi; University Hospital, Division of Infectious Diseases and Tropical Medicine, LMU Munich; German Center for Infection Research, Partner site Munich<br>Rieß, Friedrich; University Hospital, Division of Infectious Diseases and Tropical Medicine, LMU Munich; German Center for Infection Research, Partner site Munich<br>Panzner, Ursula; University Hospital, Division of Infectious Diseases and Tropical Medicine, LMU Munich; German Center for Infection Research, Partner site Munich<br>Heinrich, Norbert; University Hospital, Division of Infectious Diseases and Tropical Medicine, LMU Munich; German Center for Infection Research, Partner site Munich<br>Kranzer, Katharina; London School of Hygiene and Tropical Medicine Faculty of Infectious and Tropical Diseases; Biomedical Research and Training Institute

Keywords:	Tuberculosis < INFECTIOUS DISEASES, Diagnostic microbiology < INFECTIOUS DISEASES, RESPIRATORY MEDICINE (see Thoracic Medicine)

SCHOLARONE™
Manuscripts

Early risk assessment in pediatric and adult household contacts of confirmed tuberculosis cases by novel diagnostic tests (ERASE-TB): protocol for a prospective, non-interventional, longitudinal, multi-country cohort study

Edson Tawanda Marambire[1*], Denise Banze[2$], Alfred Mfinanga[3$], Junior Mutsvangwa[1], Theodora Mbunda[3], Elias N. Nyanda[3], Khosa Celso[2], Gunilla Kallenius[4], Claire J. Calderwood[5], Christof Geldmacher[6,7], Kathrin Held[6,7], Tejaswi Appalarowthu[6,7], Friedrich Rieß[6,7], Ursula Panzner[6,7], Norbert Heinrich[6,7#], Katharina Kranzer[1,5,6#], on behalf of the ERASE-TB Consortium

[1]Biomedical Research and Training Institute, Harare, Zimbabwe

[2]Instituto Nacional de Saúde, Marracuene, Mozambique

[3]National Institute for Medical Research - Mbeya Medical Research Centre, Mbeya, Tanzania

[4]Karolinska Institute, Stockholm, Sweden

[5]Faculty of Infectious and Tropical Diseases and Tuberculosis Centre, London School of Hygiene & Tropical Medicine, London, UK

[6]Division of Infectious Diseases and Tropical Medicine, University Hospital, LMU Munich, Munich, Germany

7 German Center for Infection Research (DZIF), Partner site Munich, Munich, Germany

*Corresponding author: Edson Tawanda Marambire; 8 Rose Avenue, Belgravia, Harare, Zimbabwe; e-mail: edsonmarambire@gmail.com; phone: +263772286311

$ Contributed equally

# Contributed equally

Word count: 3,254

Keywords: *Mycobacterium tuberculosis*, diagnostics, cohort study, household contacts, WHO END TB strategy, ERASE-TB

For peer review only

ABSTRACT

Introduction

The World Health Organization (WHO) End-TB Strategy calls for the development of novel diagnostics to detect tuberculosis earlier and more accurately. Better diagnostics, together with tools to predict disease progression are critical for achieving WHO END-TB targets. The Early Risk Assessment in TB contactS by new diagnostic tEsts (ERASE-TB) study aims to evaluate novel diagnostics and testing algorithms for early tuberculosis diagnosis and accurate prediction of disease progression among household contacts exposed to confirmed index cases in Mozambique, Tanzania and Zimbabwe.

Methods and analysis

A total of 2,100 household contacts (HHCs) (aged ≥10 years) of adults with microbiologically-confirmed pulmonary tuberculosis will be recruited and followed up at 6-month intervals for 18 to 24 months. At each time-point a WHO symptom screen and digital chest-radiograph (dCXR) will be performed, and blood and urine samples collected. Individuals screening positive (WHO symptom screen or dCXR) will be requested to provide sputum for Xpert MTB/Rif Ultra. At baseline, HHCs will also be screened for HIV, diabetes (HbA1c), chronic lung disease (spirometry), hypertension and anaemia. Study outcomes will be co-prevalent tuberculosis (diagnosed at enrollment), incident tuberculosis (diagnosed during follow-up) or no tuberculosis at completion of follow up. Novel diagnostics will be validated using fresh and biobanked samples with a nested case control design. Cases are defined as HHCs diagnosed with tuberculosis (for early diagnosis) or with incident tuberculosis (for prediction of progression) and will be matched by age, sex and country to HHCs who remain healthy (controls). Statistical analyses will include assessment of diagnostic accuracy by constructing receiver operating curves and calculation of sensitivity and specificity.

Ethics and dissemination

For peer review only

ERASE-TB has been approved by regulatory and ethical committees in each African country and by each partner organisation. Consent, with additional assent for participants <18 years, is voluntary. Attestation by impartial witnesses is sought in case of illiteracy. Confidentiality of participants is being maintained throughout. Study findings will be presented at scientific conferences and published in peer-reviewed international journals.

Trial registration number

NCT04781257

For peer review only

Strengths and limitations of this study

Strengths

- Recruitment of highly infectious index cases aimed at maximising the number of tuberculosis (TB) diagnoses in the household contact (HHCs) cohort.
- Sequencing of *Mycobacterium tuberculosis* isolates from both index cases and HHCs allows confirmation of household transmission and thus determination of timing of the transmission event; resulting in more precise estimates of new test sensitivity compared to population-based cohorts with unknown timing of infection.
- Large sample size across three southern African countries with high HIV prevalence; including adolescents will ensure study findings are generalisable to the clinically relevant population at high risk of TB compared to studies focused on adults only.

Limitations

- Despite the large cohort of HHCs, the number of diagnosed TB cases will be small, limiting the power of the study and sub-group analyses such as by age and HIV status.
- Geographically limited to sub-Saharan Africa, therefore results may not be generalisable to other populations, including those with lower HIV prevalence such as in South-East Asia or the Americas.

For peer review only

INTRODUCTION

Tuberculosis (TB) remains a leading global public health problem, with an estimated 10 million new cases and 1.5 million deaths globally in 2020 [1]. In 2014, the World Health Assembly approved the World Health Organization (WHO) End-TB Strategy, aiming for a 90% reduction in TB incidence and 95% reduction in TB deaths by 2035 [2]. However, in 2019, three million TB cases ('the missing millions') remained undiagnosed and untreated globally, resulting in potentially avoidable morbidity, mortality and onward transmission. The Covid-19 pandemic has resulted in a large decrease in the number of people newly diagnosed with TB and reported. This has increased the diagnostic gap by a further 1.3 million, resulting in an estimated 4.2 million undiagnosed TB cases in 2020 [3]. Also, for the first time in a decade TB deaths have risen, from an estimated 1.4 million in 2019 to 1.5 million in 2020, as a result of reduced access to and provision of essential TB services including diagnostics during the Covid-19 pandemic.

Without an efficacious and safe vaccine, early detection and containment are the main tools to interrupt transmission and successfully control TB. Similar to SARS-CoV2, asymptomatic spreading of *M.tuberculosis* and subclinical but infectious disease states are a major concern in the control of airborne infectious diseases [4]. Early and accurate identification of persons with TB, combined with identification of those at risk of progression to TB and provision of targeted preventive treatment are critical to reducing TB-associated morbidity and mortality, and preventing onward transmission.

Currently available diagnostics such as sputum microscopy, mycobacterial culture and nucleic acid amplification tests are based on direct pathogen detection, thus requiring a high mycobacterial load; they therefore predominately target advanced TB when onward transmission and significant lung damage has occurred [5,6]. Further, for many patients with minimal or no symptoms, expectoration of high-quality sputum specimens remains

For peer review only

challenging, limiting the accuracy of sputum-based tests. The same holds true for young children and people living with HIV.

The **E**arly **R**isk **A**ssessment in TB Contacts by new diagno**S**tic t**E**sts (ERASE-TB) study aims to fill this diagnostic gap by evaluating new sputum and non-sputum-based TB diagnostics for early TB detection (before onward transmission occurs), as well as tools for more accurate prediction of TB progression to allow for targeted preventive therapy.

METHODS AND ANALYSES

Study objectives

ERASE-TB's primary objectives are (I) to determine the sensitivity and specificity of novel diagnostics to detect TB, in particular asymptomatic or minimally symptomatic TB; (II) to evaluate novel diagnostics for detection of likely TB progression; and (III) to enhance the performance of novel diagnostics by simulating testing algorithms coupled with individual risk estimates from a mathematical model. The secondary objectives are (I) to determine the TB prevalence among household contacts (HHCs) of infectious TB index cases (ICs) at baseline and during a 18-24 months follow-up; (II) to establish a biorepository of cryopreserved specimens from HHCs for future development and validation of diagnostic tests; and (III) to assess the association of selected chronic disease conditions and TB among HHCs.

Study endpoints

The study's primary endpoints are the presence or development of TB among HHCs with the following possible scenarios of (I) prevalent symptomatic TB at baseline, (II) incident TB during follow-up, and (III) remained healthy until study completion. An endpoint review committee will review the data and case classification before finalization.

Through the sequencing of *Mycobacterium tuberculosis* (*Mtb*) isolates, cases of co-prevalent or incident TB will be classified either as secondary, infected by the source case – the timepoint

For peer review only

of infection will be known; or as infected by another, unknown source of infection, with an unknown timepoint of infection.

Recruitment sites

Recruitment of ICs and HHCs at selected primary healthcare facilities and communities has commenced in Harare, Zimbabwe in March 2021, Maputo, Mozambique in August 2021, and Mbeya, Tanzania in September 2021. Partners of the ERASE-TB consortium are illustrated in Figure 1. All three countries have a high TB incidence ranging from 100 to 499/100,000 population [1] and HIV prevalence among adults aged 15 years and older of 5% to 20% [7]. The African research institutions have established collaborations with their respective National Tuberculosis Programs ensuring referral and approproate follow-up of TB patients. Figure 2 illustrates the geographic location of research institutions, healthcare facilities where recruitment is taking place, demographic characteristics of study populations, and estimates on TB incidence and HIV prevalence [8–15].

Study design

ERASE-TB is a non-interventional, longitudinal, prospective cohort study among HHCs aged ≥10 years exposed to highly infectious pulmonary TB ICs aged ≥18 years. Eligibility criteria are detailed in Figure 3 and the study design is shown in Figure 4. TB ICs are eligible if the bacterial load in their sputum is at least at the “medium” level according to Xpert MTB/RIF or Xpert MTB/RIF Ultra, and they have received less than seven daily doses of anti-TB treatment before enrollment. This maximises the likelihood of culturing and storing *Mtb* isolates. The total study duration will be 36 months. This includes 12-months enrollment of ICs and HHCs, and 18- to 24-months follow-ups of HHCs. Follow-up ends when a HHCs withdraws from the study, is lost to follow-up, dies, or is diagnosed with TB and referred for treatment. Scheduled or unscheduled unwell visits can be conducted physically and/or telephonically in case of abnormal finding e.g. by abnormal dCXR, or when a participant feels unwell inbetween scheduled follow-up visits.

For peer review only

Procedures

TB index cases

Following informed consent obtained, a questionnaire is administered to collect socio-demographic information, TB risk factors, and the medical history of TB, HIV and other diseases. Two spontaneous sputum samples are obtained, of which one is for mycobacterial culturing and one for storage for performing retrospectively Molecular Bacterial Load Assay (MBLA) to quantify viable *Mtb* by 16S rRNA [6]; an alternative means to quantify expectorated bacterial load for an estimate of infectiousness. Both liquid and solid mycobacterial cultures are performed on decontaminated sputum samples, with all *Mtb* isolates stored at -80 degrees for future DNA extraction and whole genome sequencing. A questionnaire on symptom duration and TB risk factors is also administered.

Household key informant

At baseline, a household key informant (either the TB index case or one of the household contacts) is identified and asked to answer questions of a household questionnaire that collects socio-economic elements like structure of the house or flat, income and household assets, and covariates possibly associated with risk of TB infection, e.g., windows/air exchange, presence of comorbid conditions, and risk factors like the source of cooking energy, and properties of the household kitchen.

Household contacts

Informed consent is obtained from all eligible adult HHCs. For HHC <18 years of age, the guardian is asked to provide informed consent, with assent also sought from children dependent on local guidance. At baseline a questionnaire is administered collecting information on socioeconomic and demographic characteristics, past medical history of TB, HIV and other diseases, exposure risk factors, smoking and alcohol history. The physical examination includes height, weight, mid-upper arm circumference and blood pressure

For peer review only

measurement. In addition, all HHCs are offered free HIV testing according to the National Guidelines. All people with confirmed HIV infection will have CD4 counts performed and be referred for TB preventive therapy. Those not yet on antiretroviral therapy (ART) and those who interrupted ART are referred for ART at local services.

Point of care HbA1c (A1cCare, SD Biosensor, Gyeonggi-do, Republic of Korea) and haemoglobin (Hemocue 301+, Hemocue, Angelholm, Sweden) tests and spirometry (including pre- and post-bronchodilation with inhaled salbutamol) are performed at baseline or the six-month visit. HHCs who did not take up HIV testing or other screening at baseline are offered these tests at each study visit. Any HHCs with test results requiring treatment or further investigations are referred for respective services.

HHCs are screened for TB using the WHO symptom questionnaire and a digital chest-radiograph (dCXR), reviewed by a clinical officer. dCXRs are not performed in pregnant HHCs. HHCs with a positive WHO symptom screening and abnormal dCXR are asked to provide sputum samples for TB investigations i.e., for GeneXpert and mycobacterial culture. Those with negative symptom screen and normal dCXR are asked to provide a spontaneous sputum sample for storage (with sputum induction performed if required).

At baseline, urine, serum, plasma, whole blood (native, and with RNA preservation in PAXgene® tubes [BD Biosciences, NJ, USA]) are stored. A finger-prick sample is taken and investigated using the Xpert TB Host Response RUO Prototype cartridge (Cepheid, Sunnyvale, CA, USA). T-cell Activation Marker Tuberculosis (TAM-TB) assay and Interferon Gamma Release Assay (IGRA; STANDARDTM F TB-Feron FIA (IFN-gamma; SD Biosensor, Republic of Korea), are performed on fresh venous blood. In Tanzania and Mozambique, storage of peripheral blood mononuclear cells for later characterization of the TB-specific immune response is also performed.

For peer review only

Procedures for follow-up and unwell visits are similar to those at baseline. Measurement of HIV status, haemoglobin, HbA1c, spirometry, CD4 count and IGRA testing are not performed at follow-up visits, unless not done previously. At the last scheduled visit all HHCs not known to have HIV are re-offered HIV testing and a spontaneous or induced sputum sample is stored for all participants.

Household contacts screening positive for TB symptoms and/or with a DCXR suggestive of TB

HHCs screening positive for symptoms and/or those with DCXRs suggestive of TB are asked for a sputum sample, which is investigated using Xpert MTB/RIF Ultra (Cepheid). If this sample is positive for *Mtb* (including a trace result), a minimum of two additional sputum samples are investigated, following decontamination, with Xpert MTB/RIF Ultra, solid and liquid culture. Isolates stored from these cultures will be sequenced for matching with the IC isolates in order to verify intra-household transmission. Sputum induction is performed for those unable to provide a spontaneous sputum sample. HHCs with microbiologically confirmed TB are referred for TB treatment to the National TB Programme.

Patient and public involvement

The ERASE-TB study sites have established Community Advisory Boards, which are voices of communities, people affected, and study participants, providing a strategic link between the communities and the study team. Community Advisory Boards meet regularly and provide feedback on design, procedures and conduct of the study. They will also be closely involved in the dissemination of study results. In addition to the Community Advisory Boards, each study site conducts community engagement activities focused on young people with the aim to foster interst in science and research, specififcally in the field of respiratory diseases/illness. This includes close partnership with schools and universities. Furthermore, planned qualitative research will specifically aim to understand the perceptions of HHCs with regards to TB diagnostics and screening.

For peer review only

Sample size

An estimated 800 to 900 TB-confirmed ICs are required for the subsequent enrollment of an anticipated 2,100 HHCs, i.e., 700 HHCs per country. Loss to follow-up of HHCs is estimated to be 10%. A total of 64 HHCs (3%) are estimated to be diagnosed with TB during the study period, based on previous active case finding studies among HHCs [16].

Novel test candidates

A range of novel test candidates targeted at pathogen detection or identification of host responses to *Mtb* are being applied, either in real-time (for all participants) or retrospectively (in a case-control design). Whilst a number of novel test candidates have been pre-specified, the ERASE-TB biobanking processes allow for addition of further candidate tests to be evaluated on stored samples as they become available.

DCXRs offer good sensitivity for diagnosis of pulmonary TB. However, high inter- and intra-investigator variability, and lack of trained interpreters present a barrier to implementation in many high-TB burden settings. Computer-aided interpretation systems, such as CAD4TB (Delft Imaging, Hertogenbosch, Netherlands) and qXR (Qure.ai, India) may increase image-reading capacity, with good performance, and serve, therefore, as a systematic screening tool to identify individuals in need of confirmatory TB tests [17,18].

Xpert MTB/RIF Ultra is a nucleic acid amplification test for *Mtb* with a lower limit of detection compared to the previous Xpert MTB/RIF generation, and, therefore, conferring higher sensitivity in paucibacillary specimens. This, however, comes at the expense of specificity, particularly in high TB incidence settings, resulting in ‘false positives’ [19]. WHO guidelines recommend Xpert MTB/RIF Ultra for TB diagnosis among adults and children acknowledging that further evaluation, particularly of the role of Xpert MTB/RIF Ultra for TB screening, is needed [20,21].

For peer review only

FLOW-TB is an advanced enzyme-linked immunosorbent assay for the detection of *Mtb* lipoarabinomannan (a mycobacterial cell wall component) in urinary specimens with results available within 65 minutes [22].

The T-cell activation marker-TB assay (TAM-TB) detects Mtb-specific CD4 T-cells through *in-vitro* antigen stimulation with *Mtb*-derived peptides, i.e., from ESAT-6 and CFP-10, followed by flow cytometry. TAM-TB discriminated latent *Mtb* infection from TB in freshly collected blood with 83% sensitivity and 96-98% specificity in previous studies. Further, TAM-TB may detect early TB disease progression up to 9 months prior to the identification of *Mtb* in sputum [23–25].

Multiple transcriptomic signatures, capturing the host response to TB, have been described as promising candidate tests for earlier TB diagnosis (up to two years before microbiological diagnosis). An individual patient data meta-analysis suggested equivalent performance of eight signatures, with 25-40% sensitivity and 92-95% specificity 0-24 months before TB diagnosis. Diagnostic accuracy of each signature improved as the interval between testing and microbiological TB diagnosis shortened [26]. Several signatures have been developed into polymerase chain reaction (PCR)-based assays to facilitate real-time implementation: the recent CORTIS trial reported sensitivity of 48% and specificity of 75% for incipient TB for the RISK-11 signature [27]. Cepheid have developed a 3-transcript TB score into a fully automated in-cartridge PCR assay performed on finger-prick blood using the Xpert platform (Xpert TB Host Response RUO Prototype cartridge). This cartridge will be evaluated using freshly collected specimens in ERASE-TB; storage of RNA-stabilised blood samples also allows for retrospective evaluation of additional transcriptomic signatures in our cohort [28,29]

An alternative approach to capture the host response to TB is through protein-based biomarker signatures. Candidate tests in this category include a serum- or plasma-based multiplex assay

For peer review only

306 assessing 13 protein biomarkers (CRP, procalcitonin[30], sTREM-1[31,32], angiopoietin-2[33,34], interleukin-6[35], TRAIL[36] and IP-10[37]) that is being developed by the London School of Hygiene and Tropical Medicine; in additon, a seven biomarker signauture is under development as a point-of-care test for TB diagnosis, with 94% sensitivity and 73% specificity detected in previous work [38].

Statistical analyses

Baseline characteristics and analytical data will be summarized using descriptive statistics inclusive of mean, median, range, standard deviation, and absolute as well as relative frequencies depending on the nature of data. A logistic regression model will be used to identify characteristics of TB among ICs, households and HHCs that are predictive of incident TB. From the study database, we will simulate algorithms of different tests to obtain the testing combination with the best accuracy. We will couple tests with a mathematical model that quantifies the risk of infection and/or disease to enhance predictive performance. The reporting of the development of the prediction model will follow the Transparent Reporting of a multivariable prediction model for Individual Prognosis Or Diagnosis (TRIPOD) Initiative [39].

The validation of novel diagnostic tests for detecting TB will be analysed as a 1:4 matched nested case-control study with HHCs diagnosed with TB at baseline and during follow-up serving as cases, and HHCs who do not develop TB during follow-up as controls; controls will be matched for site, age, sex, HIV status and other risk factors for developing TB. Sensitivity and specificity of novel tests will be determined using pre-existing positive/negative cut-offs where these exist; and receiver operating curves (ROC) constructed with area under the ROC curve calculated. For tests aiming to identify individuals at high risk of TB in the future, only HHCs who are diagnosed with TB during follow-up will serve as cases (i.e. those diagnosed with TB at baseline will be excluded). Stored samples from all timepoints will be retrieved and diagnostic accuracy (i.e. sensitivity and specificity) of the novel test determined at different

For peer review only

time-points before TB diagnosis. The decision of assigning the “active TB” endpoints to participants will be blinded from the new test results to avoid inclusion bias.

Data management

All source data will be kept confidential in secured locations with restricted access by authorized personnel only inclusive of monitors, auditors and reviewers of ethical and regulatory committees in line with applicable data privacy regulations. Each participant is asked to consent to this handling of the data, and is assigned a pseudonymous identification number that is used throughout the study on all source data.

Accurate documentation of paper-based and electronic source data, e.g., original records and certified copies of original records, progress notes, screening logs, and recorded data from automated instruments, will be maintained. The pseudonymized clinical data captured on paper-based Case Report Forms will be entered at the sites into a database using the web-based Clinical Data Management System of OpenClinica (OpenClinica LLC, Waltham, MA, USA). The study specific database has been built, maintained and hosted by the LMU Klinikum on a centralized secure server. Data modifications and necessary corrections performed in the database also within the context of double data entry will be documented and tracked in audit trails. Data quality and plausibility are assured by a series of pre-programmed edit and range checks in OpenClinica. Further validation checks are programmed in Stata (Statacorp, College Station, TX, USA) with extracts of the database and electronically received data, e.g., spirometry, dCXR and laboratory, will be integrated into analyses of datasets.

Monitoring

Assigned study monitors will visit the sites at regular intervals physically and/or virtually in addition to frequent day-to-day communication. Close follow-up on all study-related aspects will be performed to ascertain compliance with standards of Good Clinical Practice, the Declaration of Helsinki, and other local and national regulatory guidelines inclusive of

For peer review only

guidelines for infection prevention and control of airborne-transmitted diseases, e.g., social distancing in well-ventilated spaces, and wearing of personal protective equipment. In particular, monitors that support designated study personnel are responsible to verify (I) adequacy of the study personnels' qualifications and facilities, (II) accuracy of informed consent procedures and patient eligibility, (III) adherence to the study protocol, (IV) protection of rights and well-being of participants, (V) adherence to infection prevention and control measures (VI) accuracy and completeness of study documents and other study-related records, and (VII) maintenance of source documents.

Ethics and dissemination

The study protocol and informed consent/assent documents have been approved by regulatory and ethical committees of the participating institutions. This includes the Medical Research Council in Zimbabwe, the National Health Research Ethics Committee in Tanzania, the National Bioethics for Health Committee in Mozambique, and the ethical committees of London School of Hygiene & Tropical Medicine, United Kingdom, and the medical faculty of the Ludwig-Maximilians-Universität München, Germany.

Adult ICs and HHCs are asked for written informed consent prior to their participation. Underage HHCs are asked for assent in addition to obtaining the consent of their legal guardians/parents; with ages for assent depending on local guidance. In case of illiteracy, the participant is asked to give its consent by fingerprint while an adult impartial, literate witness present during the entire consent procedure signs the consent on behalf. All participants have the right to withdraw from the study at any time. Findings derived from ERASE-TB will be presented at scientific conferences, and published in peer-reviewed international journals.

Current study status

The recruitment of ICs and HHCs is in progress in Zimbabwe, Mozambique and Tanzania since March, August and September 2021, respectively. The follow-up of HHCs is anticipated

For peer review only

to be completed in March, August and September 2023 in Zimbabwe, Mozambique and Tanzania, respectively; laboratory analyses are estimated to be performed by December 2024.

Author contributions

The study proposal and protocol were written by NH, KK with scientific input from CK, TM, CG, JM. ETM, UP, DB, AM wrote the initial manuscript with scientific input on the database and data management section from FR, TA. NH, KK critically reviewed the initial draft of the manuscript. KH, GK, CJC, ENN, TA, provided critical feedback on the manuscript. All authors have read and approved the final version of the manuscript.

Funding statement

ERASE-TB is part of the EDCTP2 programme supported by the European Union (grant number RIA2018D-2508-ERASE-TB), the German Center for Infection Research (DZIF) grant number: 02.710 and the Swedish Research Council (220-23602). CJC is funded by the Wellcome Trust (203905/Z/16/Z). Cepheid, Inc., and SD Biosensor provided test kits and analyzers at no cost to the Consortium.

Acknowledgments

We are grateful to the study personnel from the Biomedical Research and Training Institute and the Zvitambo Research Institute, Zimbabwe, the Instituto Nacional de Saúde, Mozambique, and the National Institute for Medical Research - Mbeya Medical Research Centre, Tanzania for their exceptional efforts and contributions, which made this research possible.

Consortium authorship

The following are members of the ERASE-TB consortium: Anna Shepherd[a], Hazel M Dockrell[a], Judith Bruchfeld[b], Christopher Sundling[b], Charles Sandy[c], Mishelle Mugava[c], Tsitsi Bandason[c], Martha Chipinduro[c], Kuda Mutasa[d], Sandra Rukobo[d], Lwitiho Sudi[e], Antelmo

For peer review only

Haule[e], Emmanuel Sichone[e], Paschal Qwaray[e], Bariki Mtafya[e], Harrieth Mwambola[e], Lilian Minja[e], Issa Sabi[e], Peter Edwin[e], Dogo Ngalison[e], Stella Luswema[e], Willyhelmina Olomi[e], Doreen Pamba[e], Simeon Mwanyonga[e], Celina Nhamuave[f], António Machiana[f], Carla Madeira[f], Emelva Manhiça[f], Nádia Sitoe[f], Jorge Ribeiro[f], Christof Geldmacher[g], Andrea Rachow[g], Olena Ivanova[g], Laura Olbrich[g], Elmar Saathoff[g], Michael Hoelscher[g].

[a]Faculty of Infectious and Tropical Diseases, Department of Infection Biology, London School of Hygiene & Tropical Medicine, London, UK

[b]Karolinska Institute, Stockholm, Sweden

[c]Biomedical Research and Training Institute, Harare, Zimbabwe

[d]Zvitambo Research Institute, Harare, Zimbabwe

[e]National Institute for Medical Research - Mbeya Medical Research Centre, Mbeya, Tanzania

[f]Instituto Nacional de Saúde, Marracuene, Mozambique

[g]Division of Infectious Diseases and Tropical Medicine, University Hospital, LMU Munich, Munich, Germany

Competing interests statement

None declaired.

Disclaimer

Not applicable.

For peer review only

References

1 Global tuberculosis report 2021. https://www.who.int/publications/i/item/9789240037021 (accessed 28 Oct 2021).

2 The end TB STraTegy global strategy and targets for tuberculosis prevention, care and control after 2015 a. Published Online First: 2014.https://www.who.int/tb/strategy/End_TB_Strategy.pdf (accessed 28 Oct 2021).

3 WHO. Global Tuberculosis Report. 2020.https://www.who.int/publications/i/item/9789240013131 (accessed 31 Jul 2021).

4 Snider B, Patel B, McBean E. Asymptomatic Cases, the Hidden Challenge in Predicting COVID-19 Caseload Increases. *Infect Dis Reports 2021, Vol 13, Pages 340-347* 2021;**13**:340–7. doi:10.3390/IDR13020033

5 TB Disease - Symptoms, treatment & prevention - TBFacts. https://tbfacts.org/tb-disease/ (accessed 28 Aug 2021).

6 Honeyborne I, McHugh TD, Phillips PPJ, *et al.* Molecular Bacterial Load Assay, a Culture-Free Biomarker for Rapid and Accurate Quantification of Sputum Mycobacterium tuberculosis Bacillary Load during Treatment. *J Clin Microbiol* 2011;**49**:3905. doi:10.1128/JCM.00547-11

7 UNIAIDS. UNAIDS Global AIDS Update. 2021. https://www.unaids.org/sites/default/files/media_asset/2021-global-aids-update_en.pdf (accessed 28 Oct 2021).

8 ZIMBABWE POPULATION CENSUS 2012 WOMEN AND MEN PROFILE SUMMARY REPORT. Published Online First: 2016.https://www.zimstat.co.zw/wp-content/uploads/publications/Social/Gender/Women-and-Men-Report-2012.pdf (accessed 15 Oct 2021).

9 Tanzania Census 2012 - Dashboard. http://dataforall.org/dashboard/tanzania/ (accessed 15 Oct 2021).

10 ZIMBABWE POPULATION-BASED HIV IMPACT ASSESSMENT ZIMPHIA 2020 KEY FINDINGS. https://phia.icap.columbia.edu/wp-content/uploads/2020/11/ZIMPHIA-2020-Summary-Sheet_Web.pdf (accessed 3 Sep 2021).

11 Xadreque H, Maunze A, Dade M, *et al.* IV RECENSEAMENTO GERAL DA POPULAÇÃO E HABITAÇÃO. 2017.http://www.ine.gov.mz/iv-rgph-2017/mocambique/censo-2017-brochura-dos-resultados-definitivos-do-iv-rgph-nacional.pdf (accessed 31 Jul 2021).

12 Population of Cities in Zimbabwe (2021). https://worldpopulationreview.com/countries/cities/zimbabwe (accessed 3 Aug 2021).

13 World atlas. https://knoema.com/atlas/United-Republic-of-Tanzania. (accessed 26 Jul 2021).

14 Prevalence of HIV, total (% of population ages 15-49) | Data. https://data.worldbank.org/indicator/SH.DYN.AIDS.ZS?locations=ZW (accessed 3 Aug 2021).

15 Incidence of tuberculosis (per 100,000 people) - Zimbabwe | Data. https://data.worldbank.org/indicator/SH.TBS.INCD?locations (accessed 3 Aug 2021).

16 Fox GJ, Nhung N V., Sy DN, *et al.* Household-Contact Investigation for Detection of Tuberculosis in Vietnam. *N Engl J Med* 2018;**378**:221–9. doi:10.1056/NEJMOA1700209

17 Qin ZZ, Ahmed S, Sarker MS, *et al.* Tuberculosis detection from chest x-rays for triaging in a high tuberculosis-burden setting: an evaluation of five artificial intelligence algorithms. *Lancet Digit Heal* 2021;**3**:e543–54. doi:10.1016/S2589-7500(21)00116-3

18 MacPherson P, Webb EL, Kamchedzera W, *et al.* Computer-aided X-ray screening for tuberculosis and HIV testing among adults with cough in Malawi (the PROSPECT study): A randomised trial and cost-effectiveness analysis. *PLoS Med* 2021;**18**:1–17. doi:10.1371/journal.pmed.1003752

19 Dorman S, Schumacher S, Alland D, *et al.* Xpert MTB/RIF Ultra for detection of Mycobacterium tuberculosis and rifampicin resistance: a prospective multicentre diagnostic accuracy study. *Lancet Infect Dis* 2018;**18**:76–84. doi:10.1016/S1473-

For peer review only

3099(17)30691-6

20 WHO Meeting Report of a Technical Expert Consultation: Non-inferiority analysis of Xpert MTB/RIF Ultra compared to Xpert MTB/RIF. Published Online First: 2017.http://apps.who.int/bookorders. (accessed 19 Oct 2021).

21 World Health Organization. WHO consolidated guidelines on tuberculosis: Module 2: Screening. 2021.https://www.who.int/publications/i/item/9789240022676 (accessed 9 Dec 2021).

22 Hamasur B, Bruchfeld J, van Helden P, *et al.* A sensitive urinary lipoarabinomannan test for tuberculosis. *PLoS One* 2015;**10**. doi:10.1371/JOURNAL.PONE.0123457

23 Portevin D, Moukambi F, Clowes P, *et al.* Assessment of the novel T-cell activation marker-tuberculosis assay for diagnosis of active tuberculosis in children: a prospective proof-of-concept study. *Lancet Infect Dis* 2014;**14**:931–8. doi:10.1016/S1473-3099(14)70884-9

24 Schuetz A, Haule A, Reither K, *et al.* Monitoring CD27 expression to evaluate Mycobacterium tuberculosis activity in HIV-1 infected individuals in vivo. *PLoS One* 2011;**6**. doi:10.1371/JOURNAL.PONE.0027284

25 Ahmed MIM, Ntinginya NE, Kibiki G, *et al.* Phenotypic Changes on Mycobacterium Tuberculosis-Specific CD4 T Cells as Surrogate Markers for Tuberculosis Treatment Efficacy. *Front Immunol* 2018;**9**:2247. doi:10.3389/FIMMU.2018.02247/BIBTEX

26 Gupta RK, Turner CT, Venturini C, *et al.* Concise whole blood transcriptional signatures for incipient tuberculosis: a systematic review and patient-level pooled meta-analysis. *Lancet Respir Med* 2020;**8**:395–406. doi:http://dx.doi.org/10.1016/S2213-2600%2819%2930282-6

27 Scriba TJ, Fiore-Gartland A, Penn-Nicholson A, *et al.* Biomarker-guided tuberculosis preventive therapy (CORTIS): a randomised controlled trial. *Lancet Infect Dis* 2021;**21**:354–65. doi:10.1016/S1473-3099(20)30914-2/ATTACHMENT/EB270186-669A-4F82-8A80-FB38FA1FB796/MMC1.PDF

28 Warsinske H, Rao A, Moreira F, *et al.* Assessment of Validity of a Blood-Based 3-Gene Signature Score for Progression and Diagnosis of Tuberculosis, Disease Severity, and Treatment Response. *JAMA Netw open* 2018;**1**:e183779. doi:10.1001/JAMANETWORKOPEN.2018.3779

29 Sweeney TE, Braviak L, Tato CM, *et al.* Genome-wide expression for diagnosis of pulmonary tuberculosis: a multicohort analysis. *Lancet Respir Med* 2016;**4**:213–24. doi:10.1016/S2213-2600(16)00048-5

30 Mendelson F, Griesel R, Tiffin N, *et al.* C-reactive protein and procalcitonin to discriminate between tuberculosis, Pneumocystis jirovecii pneumonia, and bacterial pneumonia in HIV-infected inpatients meeting WHO criteria for seriously ill: a prospective cohort study. *BMC Infect Dis* 2018;**18**. doi:10.1186/S12879-018-3303-6

31 Huang C, Lee L, Ho C, *et al.* High serum levels of procalcitonin and soluble TREM-1 correlated with poor prognosis in pulmonary tuberculosis. *J Infect* 2014;**68**:440–7. doi:10.1016/J.JINF.2013.12.012

32 Feng J, Su W, Pan S, *et al.* Role of TREM-1 in pulmonary tuberculosis patients- analysis of serum soluble TREM-1 levels. *Sci Rep* 2018;**8**. doi:10.1038/S41598-018-26478-2

33 Kumar NP, Velayutham B, Nair D, *et al.* Angiopoietins as biomarkers of disease severity and bacterial burden in pulmonary tuberculosis. *Int J Tuberc Lung Dis* 2017;**21**:93. doi:10.5588/IJTLD.16.0565

34 Kathamuthu G, Moideen K, Baskaran D, *et al.* Tuberculous lymphadenitis is associated with altered levels of circulating angiogenic factors. *Int J Tuberc Lung Dis* 2018;**22**:557–66. doi:10.5588/IJTLD.17.0609

35 Sivro A, McKinnon L, Yende-Zuma N, *et al.* Plasma Cytokine Predictors of Tuberculosis Recurrence in Antiretroviral-Treated Human Immunodeficiency Virus-infected Individuals from Durban, South Africa. *Clin Infect Dis* 2017;**65**:819–26. doi:10.1093/CID/CIX357

36 La Manna M, Orlando V, Li Donni P, *et al.* Identification of plasma biomarkers for discrimination between tuberculosis infection/disease and pulmonary non tuberculosis

For peer review only

disease. *PLoS One* 2018;**13**. doi:10.1371/JOURNAL.PONE.0192664
37 Sivro A, McKinnon L, Yende-Zuma N, *et al.* Plasma Cytokine Predictors of Tuberculosis Recurrence in Antiretroviral-Treated Human Immunodeficiency Virus-infected Individuals from Durban, South Africa. *Clin Infect Dis* 2017;**65**:819–26. doi:10.1093/CID/CIX357
38 Chegou NN, Sutherland JS, Malherbe S, *et al.* Diagnostic performance of a seven-marker serum protein biosignature for the diagnosis of active TB disease in African primary healthcare clinic attendees with signs and symptoms suggestive of TB. *Thorax* 2016;**71**:785–94. doi:10.1136/thoraxjnl-2015-207999
39 Collins GS, Reitsma JB, Altman DG, *et al.* Transparent reporting of a multivariable prediction model for individual prognosis or diagnosis (TRIPOD): The TRIPOD Statement. *BMC Med* 2015;**13**:1–10. doi:10.1186/S12916-014-0241-Z/TABLES/1
40 Agency ZS. QUALITY CONTROL MANUAL Provincial Report Harare Central Census Office. https://www.zimstat.co.zw/wp-content/uploads/publications/Population/population/Harare.pdf (accessed 3 Aug 2021).
41 Mozambique Population 2021 (Demographics, Maps, Graphs). https://worldpopulationreview.com/countries/mozambique-population (accessed 27 Jul 2021).
42 ZIMBABWE NATIONAL HIV/AIDS AND TUBERCULOSIS CONTROL PROGRAMMES. Republic of Zimbabwe NATIONAL HIV/AIDS AND TUBERCULOSIS CONTROL PROGRAMMES. https://www.who.int/hiv/pub/guidelines/zimbabwe.pdf (accessed 1 Sep 2021).

For peer review only

Figure legend

Figure 1. The ERASE-TB consortium

Abbreviations: ERASE-TB=Early Risk Assessment in TB contactS by new diagnostic tEsts

Figure 2. Location and characteristics of ERASE-TB study sites

Notes: The location of each study site is indicated by a red asterix. Source data used within this figure are taken from the references [7,8,41,42,9–15,40].

Abbreviations: ERASE=Early Risk Assessment in TB contactS by new diagnostic tEsts; TB=tuberculosis; HIV=human immunodeficiency virus

Figure 3. Eligibility criteria and schedules of events for index cases and household contacts

Notes: **A**=depending on the time point of study enrollment and consequently on the duration available for follow-up, i.e. 18 or 24 months, the follow-up visit at 24 months ±30 days may be conditional; **B**=the follow-up visit by phone may be conducted after the last scheduled follow-up visit at 18 months ±30 days or 24 months ±30 days to assess whether symptoms suggestive of TB have occurred, TB diagnosis has been made or anti-TB treatment has been initiated; **C**=unwell visits by phone or on-site may be conducted between scheduled follow-up visits if a participant presents at a recruitment healthcare facility with signs and symptoms suggestive of TB; **D**=coached spontaneous or induced sputum collection for storage at scheduled follow-up visit at 18 months ±30 days or 24 months ±30 days, and for repetition of HIV testing if tested negative at baseline; **E**=coached spontaneous or induced sputum collection upon the decision of the investigating team for testing by Xpert MTB/RIF Ultra if participant presents with signs and symptoms suggestive of TB; **F**=coached spontaneous or induced sputum collection in case of Xpert MTB/RIF Ultra positivity or strong clinical suspicion of TB for repetition of the Xpert MTB/RIF Ultra; **G**=in case of HIV positivity to be followed by the assessment of CD4 counts; **H**=CXR to be conducted at an unscheduled on-site unwell visit upon the decision of the investigating team depending on the nature of symptoms reported, and the time elapsed since the last CXR including its findings; **I**=not to be conducted among pregnant women;

For peer review only

J=stored venous blood includes 6mL EDTA blood for whole blood and plasma, 4mL serum and 2.5mL PAXgene blood, all samples will be deep frozen for retrospective testing using new diagnostics as described in text; **K**=in case the evaluation of symptoms of a participant unable to present at a recruitment healthcare facility is required an unscheduled on-site or home visit will be arranged by phone, the resolution of symptoms can alternatively be addressed by phone; **L**=collection of PBMC at baseline and follow-up visit at 6 months ±30 days is optional, thus will not be performed at each participating site and for each participant; **M**=in case the evaluation of symptoms of a participant unable to present at a recruitment healthcare facility is required or doubtful if required an unscheduled unwell visit by phone will be arranged, the resolution of symptoms can alternatively be addressed by phone; **N**=spirometry and/or diabetes (HbA1c) will be performed at scheduled follow-up visits at 6 months ±30 days, 12 months ±30 days and 18 or 24 months ±30 days if required or not performed at baseline, anaemia (Hb) will be performed at baseline and scheduled follow-up visits at 6 months ±30 days, 12 months ±30 days and 18 or 24 months ±30 days if possible; **O**=blood pressure measurement will be performed at baseline and scheduled follow-up visits at 6 months ±30 days, 12 months ±30 days and 18 or 24 months ±30 days; **P**=WGS to be performed once *Mtb* infection is confirmed and an isolate could be recovered.

Abbreviations: TB=tuberculosis; WHO=World Health Organization; HIV=human immunodeficiency virus; WGS=whole genome sequencing; MBLA=molecular bacterial load assay; CXR=chest radiograph; IGRA=interferon gamma release assay; PBMC=peripheral blood mononuclear cell; TAM-TB=T- cell activation marker tuberculosis; MTB=*Mycobacterium tuberculosis*; RIF=rifampicin, Hb=haemoglobin; HbA1c=glycated hemoglobin; LAM=lioparabinomannan; CD4=cluster of differentiation 4

Figure 4. Study design

Abbreviations: HHC=household contact; TB=tuberculosis; IC=index case; WHO=World Health Organization; CXR= chest radiograph; SS=symptom score; MTB=*Mycobacterium tuberculosis*; RIF=rifampicin; pos=positive; CT=cpmputer tomography; FU=follow-up

For peer review only

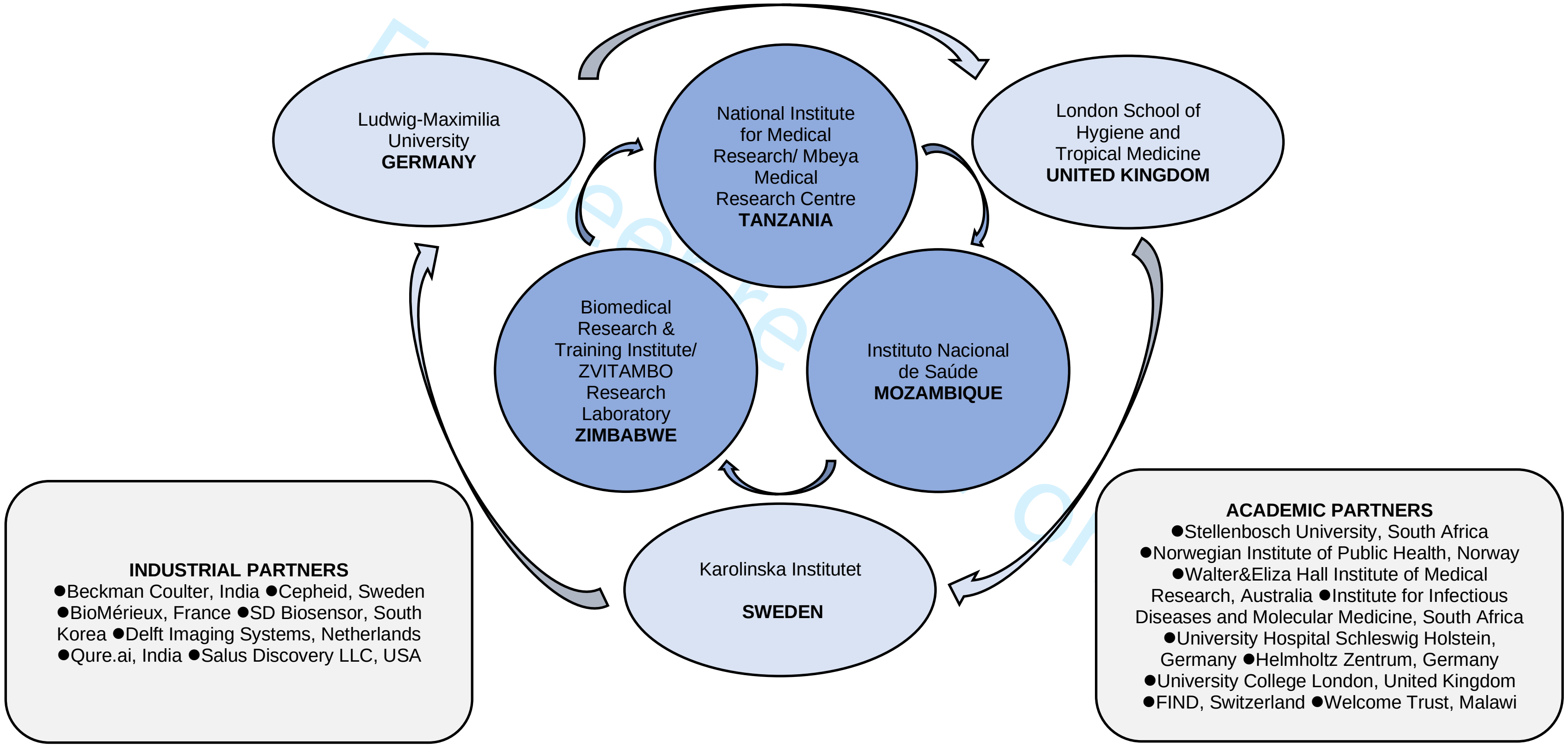

Figure 1. The ERASE-TB consortium

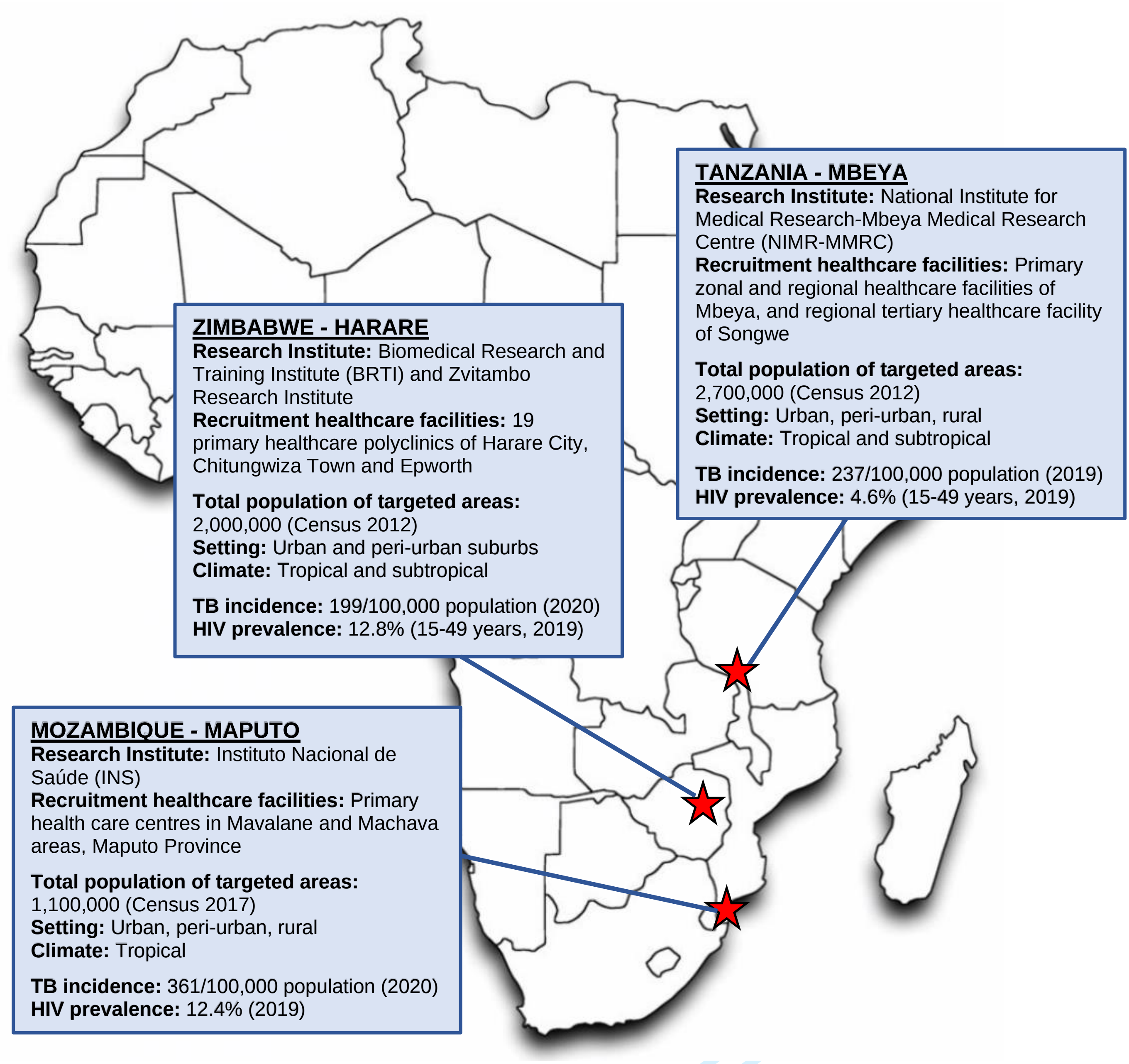

Figure 2. Location and characteristics of ERASE-TB study sites

INDEX CASE

INCLUSION CRITERIA
- Aged ≥18 years
- Living with at least another person aged ≥10 years in the same household with a firm unchanged address for 6 months
- Pulmonary TB diagnosis within 4 weeks prior to enrolment
- Spontaneous sputum production
- Positive sputum smear, i.e. >+2 positivity by Ziehl-Neelsen stain or Auramine-O stain OR ≥+1 positivity on WHO symptom scale OR ≥medium positivity by GeneXpert (if no microscopy performed)
- <7 daily doses of anti-TB treatment since diagnosis
- Voluntary written informed consent obtained

IN

EVENTS AT BASELINE/SCREENING VISIT
- Administration of socio-demographic questionnaire
- Assessment of vital signs, medical history
- Digital CXR (if available)[I]

Specimen investigations
- Sputum: culture, storage, WGS, MBLA

NO FOLLOW-UP

HOUSEHOLD CONTACT

EXCLUSION CRITERIA
- Treatment for TB within the past 30 days OR on current TB treatment OR on current preventive TB therapy (if HIV negative) at the tim of enrolment

OUT

INCLUSION CRITERIA
- Aged ≥10 years
- Substantial exposure, i.e. spending ≥3 nights/week over the past 4 weeks in the same household as the index case,
- Voluntary written informed consent or parent/guardian consent & assent in case of minors obtained

IN

EVENTS AT BASELINE/ ±14days of SCREENING VISIT
- Administration of socio-demographic questionnaire
- Assessment vital signs, medical history
- Screening for diabetes (HbA1c)[N], high blood pressure[O], anaemia (Hb)[N], WHO TB symptoms
- Digital CXR[I], spirometry[N]

Specimen investigations
- Sputum: Xpert MTB/RIF Ultra[E], culture[F], storage, WGS[P]
- Blood: HIV/CD4 counts[G], Cepheid host response, TAM-TB, IGRA SD Biosensor, PBMC[L], storage[J]
- Urine: dipstick/LAM, storage

FOLLOW-UP

OUT

OUT TB+

EVENTS AT UNSCHEDULED UNWELL VISIT(S) (phone/on-site)[C,K, M]
- Assessment of vital signs, medical history
- Screening for high blood pressure, WHO TB symptoms,
- Digital CRX[H,I]

Specimen investigations
- Sputum: Xpert MTB/RIF Ultra, culture, storage, WGS[P]
- Blood: Cepheid host response, TAM-TB, storage[J]
- Urine: dipstick/LAM, storage

EVENTS AT UN-SCHEDULED FOLLOW-UP VISIT(S)[B] (phone)
- Assessment of medical history
- Screening for WHO TB symptoms

Specimen investigations
- None

EVENTS AT 6months ±30days/ 12months ±30days/ 18months ±30days/ 24months ±30days[A] FOLLOW-UP VISITS
- Assessment of vital signs, medical history
- Screening for high blood pressure, WHO TB symptoms
- Digital CXR[I], spirometry[N]

Specimen investigations
- Sputum: Xpert MTB/RIF Ultra[E], culture[F], storage[D], WGS[P]
- Blood: Cepheid host response, TAM-TB, PBMC[L], storage
- Urine: dipstick/LAM, storage[J]

Optional **Optional**

For peer review only - http://bmjopen.bmj.com/site/about/guidelines.xhtml

Figure 3. Eligibility criteria and schedules of events for index cases and household contacts

For peer review only

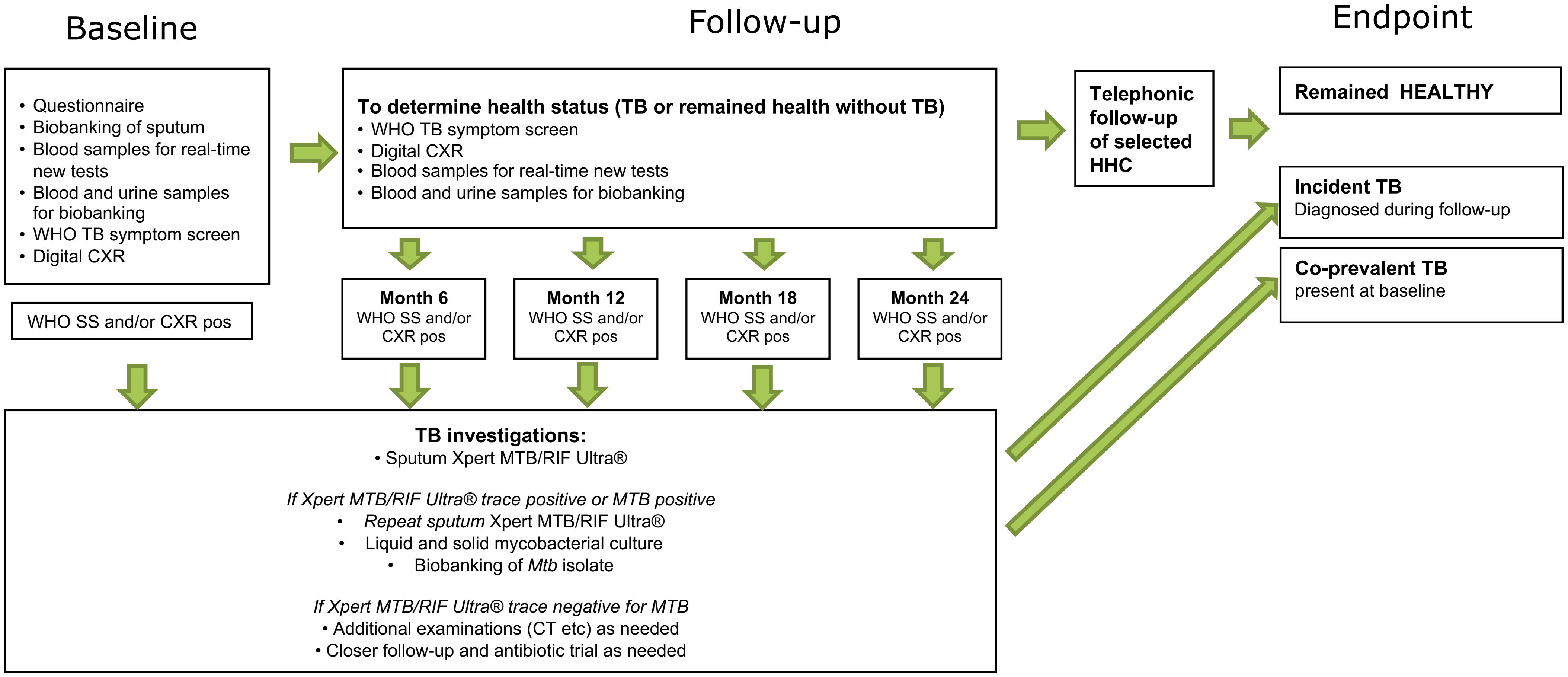

Figure 4. Study design.

BMJ Open

Early risk assessment in pediatric and adult household contacts of confirmed tuberculosis cases by novel diagnostic tests (ERASE-TB): protocol for a prospective, non-interventional, longitudinal, multi-country cohort study

Journal:	*BMJ Open*
Manuscript ID	bmjopen-2022-060985.R1
Article Type:	Protocol
Date Submitted by the Author:	31-May-2022
Complete List of Authors:	Marambire, Edson; Biomedical Research and Training Institute,<br>Banze, Denise; Instituto Nacional de Saúde<br>Mfinanga, Alfred; Mbeya Medical Research Centre<br>Mutsvangwa, Junior; Biomedical Research and Training Institute<br>Mbunda, Theodora ; NIMR-Mbeya Medical Research Programme<br>Ntinginya, Nyanda; 6. National Institute of Medical Research-Mbeya Medical Research Centre<br>Celso, Khosa; Instituto Nacional de Saúde<br>Kallenius, Gunilla; Karolinska Institutet<br>Calderwood, Claire J.; London School of Hygiene and Tropical Medicine Faculty of Infectious and Tropical Diseases<br>Geldmacher, Christof; University Hospital, Division of Infectious Diseases and Tropical Medicine, LMU Munich ; German Center for Infection Research, Partner site Munich<br>Held, Kathrin; University Hospital, Division of Infectious and Tropical Medicine, LMU Munich ; German Center for Infection Research, Partner site Munich<br>Appalarowthu, Tejaswi; University Hospital, Division of Infectious Diseases and Tropical Medicine, LMU Munich; German Center for Infection Research, Partner site Munich<br>Rieß, Friedrich; University Hospital, Division of Infectious Diseases and Tropical Medicine, LMU Munich; German Center for Infection Research, Partner site Munich<br>Panzner, Ursula; University Hospital, Division of Infectious Diseases and Tropical Medicine, LMU Munich; German Center for Infection Research, Partner site Munich<br>Heinrich, Norbert; University Hospital, Division of Infectious Diseases and Tropical Medicine, LMU Munich; German Center for Infection Research, Partner site Munich<br>Kranzer, Katharina; London School of Hygiene and Tropical Medicine Faculty of Infectious and Tropical Diseases; Biomedical Research and Training Institute
**Primary Subject Heading**:	Respiratory medicine
Secondary Subject Heading:	Diagnostics, Infectious diseases

Keywords:	Tuberculosis < INFECTIOUS DISEASES, Diagnostic microbiology < INFECTIOUS DISEASES, RESPIRATORY MEDICINE (see Thoracic Medicine)

SCHOLARONE™
Manuscripts

Early risk assessment in pediatric and adult household contacts of confirmed tuberculosis cases by novel diagnostic tests (ERASE-TB): protocol for a prospective, non-interventional, longitudinal, multi-country cohort study

Edson Tawanda Marambire[1*], Denise Banze[2$], Alfred Mfinanga[3$], Junior Mutsvangwa[1], Theodora Mbunda[3], Elias N. Nyanda[3], Khosa Celso[2], Gunilla Kallenius[4], Claire J. Calderwood[5], Christof Geldmacher[6,7], Kathrin Held[6,7], Tejaswi Appalarowthu[6,7], Friedrich Rieß[6,7], Ursula Panzner[6,7], Norbert Heinrich[6,7#], Katharina Kranzer[1,5,6#], on behalf of the ERASE-TB Consortium

[1]Biomedical Research and Training Institute, Harare, Zimbabwe

[2]Instituto Nacional de Saúde, Marracuene, Mozambique

[3]National Institute for Medical Research - Mbeya Medical Research Centre, Mbeya, Tanzania

[4]Karolinska Institute, Stockholm, Sweden

[5]Faculty of Infectious and Tropical Diseases and Tuberculosis Centre, London School of Hygiene & Tropical Medicine, London, UK

[6]Division of Infectious Diseases and Tropical Medicine, University Hospital, LMU Munich, Munich, Germany

[7]German Center for Infection Research (DZIF), Partner site Munich, Munich, Germany

*Corresponding author: Edson Tawanda Marambire; 8 Rose Avenue, Belgravia, Harare, Zimbabwe; e-mail: edsonmarambire@gmail.com; phone: +263772286311

$ Contributed equally

# Contributed equally

Word count: 3,668

Keywords: *Mycobacterium tuberculosis*, diagnostics, cohort study, household contacts, WHO END TB strategy, ERASE-TB

For peer review only

ABSTRACT

Introduction

The World Health Organization (WHO) End-TB Strategy calls for the development of novel diagnostics to detect tuberculosis earlier and more accurately. Better diagnostics, together with tools to predict disease progression are critical for achieving WHO END-TB targets. The Early Risk Assessment in TB contactS by new diagnostic tEsts (ERASE-TB) study aims to evaluate novel diagnostics and testing algorithms for early tuberculosis diagnosis and accurate prediction of disease progression among household contacts exposed to confirmed index cases in Mozambique, Tanzania and Zimbabwe.

Methods and analysis

A total of 2,100 household contacts (HHCs) (aged ≥10 years) of adults with microbiologically-confirmed pulmonary tuberculosis will be recruited and followed up at 6-month intervals for 18 to 24 months. At each time-point a WHO symptom screen and digital chest-radiograph (dCXR) will be performed, and blood and urine samples collected. Individuals screening positive (WHO symptom screen or dCXR) will be requested to provide sputum for Xpert MTB/Rif Ultra. At baseline, HHCs will also be screened for HIV, diabetes (HbA1c), chronic lung disease (spirometry), hypertension and anaemia. Study outcomes will be co-prevalent tuberculosis (diagnosed at enrollment), incident tuberculosis (diagnosed during follow-up) or no tuberculosis at completion of follow up. Novel diagnostics will be validated using fresh and biobanked samples with a nested case control design. Cases are defined as HHCs diagnosed with tuberculosis (for early diagnosis) or with incident tuberculosis (for prediction of progression) and will be matched by age, sex and country to HHCs who remain healthy (controls). Statistical analyses will include assessment of diagnostic accuracy by constructing receiver operating curves and calculation of sensitivity and specificity.

Ethics and dissemination

For peer review only

ERASE-TB has been approved by regulatory and ethical committees in each African country and by each partner organisation. Consent, with additional assent for participants <18 years, is voluntary. Attestation by impartial witnesses is sought in case of illiteracy. Confidentiality of participants is being maintained throughout. Study findings will be presented at scientific conferences and published in peer-reviewed international journals.

Study registration number

NCT04781257

For peer review only

Strengths

- Recruitment of highly infectious index cases aimed at maximising the number of tuberculosis (TB) diagnoses in the household contact (HHCs) cohort.
- Sequencing of *Mycobacterium tuberculosis* isolates from both index cases and HHCs allows confirmation of household transmission and thus determination of timing of the transmission event; resulting in more precise estimates of new test sensitivity compared to population-based cohorts with unknown timing of infection.
- Large sample size across three southern African countries with high HIV prevalence; including adolescents will ensure study findings are generalisable to the clinically relevant population at high risk of TB compared to studies focused on adults only.

Limitations

- Despite the large cohort of HHCs, the number of diagnosed TB cases will be small, limiting the power of the study and sub-group analyses such as by age and HIV status.
- Geographically limited to sub-Saharan Africa, therefore results may not be generalisable to other populations, including those with lower HIV prevalence such as in South-East Asia or the Americas.

For peer review only

INTRODUCTION

Tuberculosis (TB) remains a leading global public health problem, with an estimated 10 million new cases and 1.5 million deaths globally in 2020 [1]. In 2014, the World Health Assembly approved the World Health Organization (WHO) End-TB Strategy, aiming for a 90% reduction in TB incidence and 95% reduction in TB deaths by 2035 [2]. However, in 2019, three million TB cases ('the missing millions') remained undiagnosed and untreated globally, resulting in potentially avoidable morbidity, mortality and onward transmission. The Covid-19 pandemic has resulted in a large decrease in the number of people newly diagnosed with TB and reported. This has increased the diagnostic gap by a further 1.3 million, resulting in an estimated 4.2 million undiagnosed TB cases in 2020 [3]. Also, for the first time in a decade TB deaths have risen, from an estimated 1.4 million in 2019 to 1.5 million in 2020, as a result of reduced access to and provision of essential TB services including diagnostics during the Covid-19 pandemic.

Without an efficacious and safe vaccine, early detection and containment are the main tools to interrupt transmission and successfully control TB. Similar to SARS-CoV2, asymptomatic spreading of *M.tuberculosis* and subclinical but infectious disease states are a major concern in the control of airborne infectious diseases [4]. Early and accurate identification of persons with TB, combined with identification of those at risk of progression to TB and provision of targeted preventive treatment are critical to reducing TB-associated morbidity and mortality, and preventing onward transmission.

Currently available diagnostics such as sputum microscopy, mycobacterial culture and nucleic acid amplification tests are based on direct pathogen detection, thus requiring a high mycobacterial load; they therefore predominately target advanced TB when onward transmission and significant lung damage has occurred [5,6]. Further, for many patients with minimal or no symptoms, expectoration of high-quality sputum specimens remains

For peer review only

challenging, limiting the accuracy of sputum-based tests. The same holds true for young children and people living with HIV.

The **E**arly **R**isk **A**ssessment in TB Contacts by new diagno**S**tic t**E**sts (ERASE-TB) study aims to fill this diagnostic gap by evaluating new sputum and non-sputum-based TB diagnostics for early TB detection (before onward transmission occurs), as well as tools for more accurate prediction of TB progression to allow for targeted preventive therapy.

METHODS AND ANALYSES

Study objectives

ERASE-TB's primary objectives are (I) to determine the sensitivity and specificity of novel diagnostics to detect TB, in particular asymptomatic or minimally symptomatic TB; (II) to evaluate novel diagnostics for detection of likely TB progression; and (III) to enhance the performance of novel diagnostics by simulating testing algorithms coupled with individual risk estimates from a mathematical model. The secondary objectives are (I) to determine the TB prevalence among household contacts (HHCs) of infectious TB index cases (ICs) at baseline and during a 18-24 months follow-up; (II) to establish a biorepository of cryopreserved specimens from HHCs for future development and validation of diagnostic tests; and (III) to assess the association of selected chronic disease conditions and TB among HHCs.

Study endpoints

The study's primary endpoints are the presence or development of TB among HHCs with the following possible scenarios of (I) prevalent symptomatic TB at baseline, (II) incident TB during follow-up, and (III) remained healthy until study completion. An endpoint review committee will review the data and case classification before finalization.

Through the sequencing of *Mycobacterium tuberculosis* (*Mtb*) isolates, cases of co-prevalent or incident TB will be classified either as secondary, infected by the source case – the timepoint

For peer review only

of infection will be known; or as infected by another, unknown source of infection, with an unknown timepoint of infection.

Recruitment sites

Recruitment of ICs and HHCs at selected primary healthcare facilities and communities has commenced in Harare, Zimbabwe in March 2021, Maputo, Mozambique in August 2021, and Mbeya, Tanzania in September 2021. Partners of the ERASE-TB consortium are illustrated in Figure 1. All three countries have a high TB incidence ranging from 100 to 499/100,000 population [1] and HIV prevalence among adults aged 15 years and older of 5% to 20% [7]. The African research institutions have established collaborations with their respective National Tuberculosis Programs ensuring referral and approproate follow-up of TB patients. Figure 2 illustrates the geographic location of research institutions, healthcare facilities where recruitment is taking place, demographic characteristics of study populations, and estimates on TB incidence and HIV prevalence [8–15].

Study design

ERASE-TB is a non-interventional, longitudinal, prospective cohort study among HHCs aged ≥10 years exposed to highly infectious pulmonary TB ICs aged ≥18 years. Eligibility criteria are detailed in Figure 3 and the study design is shown in Figure 4. TB ICs are eligible if the bacterial load in their sputum is at least at the "medium" level according to Xpert MTB/RIF or Xpert MTB/RIF Ultra, and they have received less than seven daily doses of anti-TB treatment before enrollment. This maximises the likelihood of culturing and storing *Mtb* isolates. The total study duration will be 36 months. This includes 12-months enrollment of ICs and HHCs, and 18- to 24-months follow-ups of HHCs. Follow-up ends when a HHCs withdraws from the study, is lost to follow-up, dies, or is diagnosed with TB and referred for treatment. Scheduled or unscheduled unwell visits can be conducted physically and/or telephonically in case of abnormal finding e.g. by abnormal dCXR, or when a participant feels unwell inbetween scheduled follow-up visits.

For peer review only

Procedures

TB index cases

Following informed consent obtained, a questionnaire is administered to collect socio-demographic information, TB risk factors, and the medical history of TB, HIV and other diseases. Two spontaneous sputum samples are obtained, of which one is for mycobacterial culturing and one for storage for performing retrospectively Molecular Bacterial Load Assay (MBLA) to quantify viable *Mtb* by 16S rRNA [6]; an alternative means to quantify expectorated bacterial load for an estimate of infectiousness. Both liquid and solid mycobacterial cultures are performed on decontaminated sputum samples, with all *Mtb* isolates stored at -80 degrees for future DNA extraction and whole genome sequencing. A questionnaire on symptom duration and TB risk factors is also administered.

Household key informant

At baseline, a household key informant (either the TB index case or one of the household contacts) is identified and asked to answer questions of a household questionnaire that collects socio-economic elements like structure of the house or flat, income and household assets, and covariates possibly associated with risk of TB infection, e.g., windows/air exchange, presence of comorbid conditions, and risk factors like the source of cooking energy, and properties of the household kitchen.

Household contacts

Informed consent is obtained from all eligible adult HHCs. For HHC <18 years of age, the guardian is asked to provide informed consent, with assent also sought from children dependent on local guidance. At baseline a questionnaire is administered collecting information on socioeconomic and demographic characteristics, past medical history of TB, HIV and other diseases, exposure risk factors, smoking and alcohol history. The physical examination includes height, weight, mid-upper arm circumference and blood pressure

For peer review only

measurement. In addition, all HHCs are offered free HIV testing according to the National Guidelines. All people with confirmed HIV infection will have CD4 counts performed and be referred for TB preventive therapy. Those not yet on antiretroviral therapy (ART) and those who interrupted ART are referred for ART at local services.

Point of care HbA1c (A1cCare, SD Biosensor, Gyeonggi-do, Republic of Korea) and haemoglobin (Hemocue 301+, Hemocue, Angelholm, Sweden) tests and spirometry (including pre- and post-bronchodilation with inhaled salbutamol) are performed at baseline or the six-month visit. HHCs who did not take up HIV testing or other screening at baseline are offered these tests at each study visit. Any HHCs with test results requiring treatment or further investigations are referred for respective services.

HHCs are screened for TB using the WHO symptom questionnaire and a digital chest-radiograph (dCXR), reviewed by a clinical officer. dCXRs are not performed in pregnant HHCs. HHCs with a positive WHO symptom screening and abnormal dCXR are asked to provide sputum samples for TB investigations i.e., for GeneXpert and mycobacterial culture. Those with negative symptom screen and normal dCXR are asked to provide a spontaneous sputum sample for storage (with sputum induction performed if required).

At baseline, urine, serum, plasma, whole blood (native, and with RNA preservation in PAXgene® tubes [BD Biosciences, NJ, USA]) are stored. A finger-prick sample is taken and investigated using the Xpert TB Host Response RUO Prototype cartridge (Cepheid, Sunnyvale, CA, USA). T-cell Activation Marker Tuberculosis (TAM-TB) assay and Interferon Gamma Release Assay (IGRA; STANDARDTM F TB-Feron FIA (IFN-gamma; SD Biosensor, Republic of Korea), are performed on fresh venous blood. In Tanzania and Mozambique, storage of peripheral blood mononuclear cells for later characterization of the TB-specific immune response is also performed.

For peer review only

Procedures for follow-up and unwell visits are similar to those at baseline. Measurement of HIV status, haemoglobin, HbA1c, spirometry, CD4 count and IGRA testing are not performed at follow-up visits, unless not done previously. At the last scheduled visit all HHCs not known to have HIV are re-offered HIV testing and a spontaneous or induced sputum sample is stored for all participants.

Household contacts screening positive for TB symptoms and/or with a DCXR suggestive of TB

HHCs screening positive for symptoms and/or those with DCXRs suggestive of TB are asked for a sputum sample, which is investigated using Xpert MTB/RIF Ultra (Cepheid). If this sample is positive for *Mtb* (including a trace result), a minimum of two additional sputum samples are investigated, following decontamination, with Xpert MTB/RIF Ultra, solid and liquid culture. Isolates stored from these cultures will be sequenced for matching with the IC isolates in order to verify intra-household transmission. Sputum induction is performed for those unable to provide a spontaneous sputum sample. HHCs with microbiologically confirmed TB are referred for TB treatment to the National TB Programme.

Patient and public involvement

The ERASE-TB study sites have established Community Advisory Boards, which are voices of communities, people affected, and study participants, providing a strategic link between the communities and the study team. Community Advisory Boards meet regularly and provide feedback on design, procedures and conduct of the study. They will also be closely involved in the dissemination of study results. In addition to the Community Advisory Boards, each study site conducts community engagement activities focused on young people with the aim to foster interst in science and research, specififcally in the field of respiratory diseases/illness. This includes close partnership with schools and universities. Furthermore, planned qualitative research will specifically aim to understand the perceptions of HHCs with regards to TB diagnostics and screening.

For peer review only

Sample size

An estimated 800 to 900 TB-confirmed ICs are required for the subsequent enrollment of an anticipated 2,100 HHCs, i.e., 700 HHCs per country. Loss to follow-up of HHCs is estimated to be 10%. A total of 64 HHCs (3%) are estimated to be diagnosed with TB during the study period, based on previous active case finding studies among HHCs [16]. Validation for subclinical and early TB will include incident (n=49) and co-prevalent TB cases (n=15). Validation for detection of incipient *M.tb* infection will include samples of participants with incident TB (n=49) matched 1:4 to samples of participants without TB (n=196). For tests diagnosing incipient *M.tb* infection sensitivities of 73% and 82% would be detected with a precision of 59-85% and 68-91% respectively. For specifcities of 92% and 94% the confidence intervals would be 87-95% and 90-97%.

Novel test candidates

A range of novel test candidates targeted at pathogen detection or identification of host responses to *Mtb* are being applied, either in real-time (for all participants) or retrospectively (in a case-control design). Whilst a number of novel test candidates have been pre-specified, the ERASE-TB biobanking processes allow for addition of further candidate tests to be evaluated on stored samples as they become available.

DCXRs offer good sensitivity for diagnosis of pulmonary TB. However, high inter- and intra-investigator variability, and lack of trained interpreters present a barrier to implementation in many high-TB burden settings. Computer-aided interpretation systems, such as CAD4TB (Delft Imaging, Hertogenbosch, Netherlands) and qXR (Qure.ai, India) may increase image-reading capacity, with good performance, and serve, therefore, as a systematic screening tool to identify individuals in need of confirmatory TB tests [17,18].

For peer review only

Xpert MTB/RIF Ultra is a nucleic acid amplification test for *Mtb* with a lower limit of detection compared to the previous Xpert MTB/RIF generation, and, therefore, conferring higher sensitivity in paucibacillary specimens. This, however, comes at the expense of specificity, particularly in high TB incidence settings, resulting in 'false positives' [19]. WHO guidelines recommend Xpert MTB/RIF Ultra for TB diagnosis among adults and children acknowledging that further evaluation, particularly of the role of Xpert MTB/RIF Ultra for TB screening, is needed [20,21].

FLOW-TB is an advanced enzyme-linked immunosorbent assay for the detection of *Mtb* lipoarabinomannan (a mycobacterial cell wall component) in urinary specimens with results available within 65 minutes [22].

The T-cell activation marker-TB assay (TAM-TB) detects Mtb-specific CD4 T-cells through *in-vitro* antigen stimulation with *Mtb*-derived peptides, i.e., from ESAT-6 and CFP-10, followed by flow cytometry. TAM-TB discriminated latent *Mtb* infection from TB in freshly collected blood with 83% sensitivity and 96-98% specificity in previous studies. Further, TAM-TB may detect early TB disease progression up to 9 months prior to the identification of *Mtb* in sputum [23–25].

Multiple transcriptomic signatures, capturing the host response to TB, have been described as promising candidate tests for earlier TB diagnosis (up to two years before microbiological diagnosis). An individual patient data meta-analysis suggested equivalent performance of eight signatures, with 25-40% sensitivity and 92-95% specificity 0-24 months before TB diagnosis. Diagnostic accuracy of each signature improved as the interval between testing and microbiological TB diagnosis shortened [26]. Several signatures have been developed into polymerase chain reaction (PCR)-based assays to facilitate real-time implementation: the recent CORTIS trial reported sensitivity of 48% and specificity of 75% for incipient TB for the RISK-11 signature [27]. Cepheid have developed a 3-transcript TB score into a fully automated

For peer review only

in-cartridge PCR assay performed on finger-prick blood using the Xpert platform (Xpert TB Host Response RUO Prototype cartridge). This cartridge will be evaluated using freshly collected specimens in ERASE-TB; storage of RNA-stabilised blood samples also allows for retrospective evaluation of additional transcriptomic signatures in our cohort [28,29]

An alternative approach to capture the host response to TB is through protein-based biomarker signatures. Candidate tests in this category include a serum- or plasma-based multiplex assay assessing 13 protein biomarkers (CRP, procalcitonin[30], sTREM-1[31,32], angiopoietin-2[33,34], interleukin-6[35], TRAIL[36] and IP-10[37]) that is being developed by the London School of Hygiene and Tropical Medicine; in additon, a seven biomarker signauture is under development as a point-of-care test for TB diagnosis, with 94% sensitivity and 73% specificity detected in previous work [38].

Statistical analyses

Baseline characteristics and analytical data will be summarized using descriptive statistics inclusive of mean, median, range, standard deviation, and absolute as well as relative frequencies depending on the nature of data. A logistic regression model will be used to identify characteristics of TB among ICs, households and HHCs that are predictive of incident TB. From the study database, we will simulate algorithms of different tests to obtain the testing combination with the best accuracy. We will couple tests with a mathematical model that quantifies the risk of infection and/or disease to enhance predictive performance. The reporting of the development of the prediction model will follow the Transparent Reporting of a multivariable prediction model for Individual Prognosis Or Diagnosis (TRIPOD) Initiative [39].

The validation of novel diagnostic tests for detecting TB will be analysed as a 1:4 matched nested case-control study with HHCs diagnosed with TB at baseline and during follow-up serving as cases, and HHCs who do not develop TB during follow-up as controls; controls will be matched for site, age, sex, HIV status and other risk factors for developing TB. Sensitivity

For peer review only

and specificity of novel tests will be determined using pre-existing positive/negative cut-offs where these exist [40]; and receiver operating curves (ROC) constructed with area under the ROC curve calculated. For tests aiming to identify individuals at high risk of TB in the future, only HHCs who are diagnosed with TB during follow-up will serve as cases (i.e. those diagnosed with TB at baseline will be excluded). Stored samples from all timepoints will be retrieved and diagnostic accuracy (i.e. sensitivity and specificity) of the novel test determined at different time-points before TB diagnosis. The decision of assigning the “active TB” endpoints to participants will be blinded from the new test results to avoid inclusion bias.

Data management

All source data will be kept confidential in secured locations with restricted access by authorized personnel only inclusive of monitors, auditors and reviewers of ethical and regulatory committees in line with applicable data privacy regulations. Each participant is asked to consent to this handling of the data, and is assigned a pseudonymous identification number that is used throughout the study on all source data.

Accurate documentation of paper-based and electronic source data, e.g., original records and certified copies of original records, progress notes, screening logs, and recorded data from automated instruments, will be maintained. The pseudonymized clinical data captured on paper-based Case Report Forms will be entered at the sites into a database using the web-based Clinical Data Management System of OpenClinica (OpenClinica LLC, Waltham, MA, USA). The study specific database has been built, maintained and hosted by the LMU Klinikum on a centralized secure server. Data modifications and necessary corrections performed in the database also within the context of double data entry will be documented and tracked in audit trails. Data quality and plausibility are assured by a series of pre-programmed edit and range checks in OpenClinica. Further validation checks are programmed in Stata (Statacorp, College Station, TX, USA) with extracts of the database and electronically received data, e.g., spirometry, dCXR and laboratory, will be integrated into analyses of datasets.

For peer review only

Monitoring

Assigned study monitors will visit the sites at regular intervals physically and/or virtually in addition to frequent day-to-day communication. Close follow-up on all study-related aspects will be performed to ascertain compliance with standards of Good Clinical Practice, the Declaration of Helsinki, and other local and national regulatory guidelines inclusive of guidelines for infection prevention and control of airborne-transmitted diseases, e.g., social distancing in well-ventilated spaces, and wearing of personal protective equipment. In particular, monitors that support designated study personnel are responsible to verify (I) adequacy of the study personnels' qualifications and facilities, (II) accuracy of informed consent procedures and patient eligibility, (III) adherence to the study protocol, (IV) protection of rights and well-being of participants, (V) adherence to infection prevention and control measures (VI) accuracy and completeness of study documents and other study-related records, and (VII) maintenance of source documents.

Ethics and dissemination

The study protocol and informed consent/assent documents have been approved by regulatory and ethical committees of the participating institutions [Medical Research Council in Zimbabwe (MRCZ/A/2618), the National Health Research Ethics Committee in Tanzania (TMDA-WEB0021/CTR/0004/03), the National Bioethics Committee for Health in Mozambique (541/CNBS/21), and the ethical committees of London School of Hygiene & Tropical Medicine, United Kingdom (22522-2), and the medical faculty of the Ludwig-Maximilians-Universität München, Germany (20-0771)].

Adult ICs and HHCs are asked for written informed consent prior to their participation. Underage HHCs are asked for assent in addition to obtaining the consent of their legal guardians/parents; with ages for assent depending on local guidance. In case of illiteracy, the participant is asked to give its consent by fingerprint while an adult impartial, literate witness

For peer review only

present during the entire consent procedure signs the consent on behalf. All participants have the right to withdraw from the study at any time. Findings derived from ERASE-TB will be presented at scientific conferences, and published in peer-reviewed international journals.

Current study status

The recruitment of ICs and HHCs is in progress in Zimbabwe, Mozambique and Tanzania since March, August and September 2021, respectively. The follow-up of HHCs is anticipated to be completed in March, August and September 2023 in Zimbabwe, Mozambique and Tanzania, respectively; laboratory analyses are estimated to be performed by December 2024.

Author contributions

The study proposal and protocol were written by NH, KK with scientific input from CK, TM, CG, JM. ETM, UP, DB, AM wrote the initial manuscript with scientific input on the database and data management section from FR, TA. NH, KK critically reviewed the initial draft of the manuscript. KH, GK, CJC, ENN, TA, provided critical feedback on the manuscript. All authors have read and approved the final version of the manuscript.

Funding statement

ERASE-TB is part of the EDCTP2 programme supported by the European Union (grant number RIA2018D-2508-ERASE-TB), the German Center for Infection Research (DZIF) grant number: 02.710 and the Swedish Research Council (220-23602). CJC is funded by the Wellcome Trust (203905/Z/16/Z). Cepheid, Inc., and SD Biosensor provided test kits and analyzers at no cost to the Consortium.

Acknowledgments

We are grateful to the study personnel from the Biomedical Research and Training Institute and the Zvitambo Research Institute, Zimbabwe, the Instituto Nacional de Saúde, Mozambique, and the National Institute for Medical Research - Mbeya Medical Research

For peer review only

Centre, Tanzania for their exceptional efforts and contributions, which made this research possible.

Consortium authorship

The following are members of the ERASE-TB consortium: Anna Shepherd[a], Hazel M Dockrell[a], Judith Bruchfeld[b], Christopher Sundling[b], Charles Sandy[c], Mishelle Mugava[c], Tsitsi Bandason[c], Martha Chipinduro[c], Kuda Mutasa[d], Sandra Rukobo[d], Lwitiho Sudi[e], Antelmo Haule[e], Emmanuel Sichone[e], Paschal Qwaray[e], Bariki Mtafya[e], Harrieth Mwambola[e], Lilian Minja[e], Issa Sabi[e], Peter Edwin[e], Dogo Ngalison[e], Stella Luswema[e], Willyhelmina Olomi[e], Doreen Pamba[e], Simeon Mwanyonga[e], Celina Nhamuave[f], António Machiana[f], Carla Madeira[f], Emelva Manhiça[f], Nádia Sitoe[f], Jorge Ribeiro[f], Christof Geldmacher[g], Andrea Rachow[g], Olena Ivanova[g], Laura Olbrich[g], Elmar Saathoff[g], Michael Hoelscher[g].

[a]Faculty of Infectious and Tropical Diseases, Department of Infection Biology, London School of Hygiene & Tropical Medicine, London, UK

[b]Karolinska Institute, Stockholm, Sweden

[c]Biomedical Research and Training Institute, Harare, Zimbabwe

[d]Zvitambo Research Institute, Harare, Zimbabwe

[e]National Institute for Medical Research - Mbeya Medical Research Centre, Mbeya, Tanzania

[f]Instituto Nacional de Saúde, Marracuene, Mozambique

[g]Division of Infectious Diseases and Tropical Medicine, University Hospital, LMU Munich, Munich, Germany

Competing interests statement

None declaired.

Disclaimer

Not applicable.

For peer review only

References

1 Global tuberculosis report 2021. https://www.who.int/publications/i/item/9789240037021 (accessed 28 Oct 2021).

2 The end TB STraTegy global strategy and targets for tuberculosis prevention, care and control after 2015 a. Published Online First: 2014.https://www.who.int/tb/strategy/End_TB_Strategy.pdf (accessed 28 Oct 2021).

3 WHO. Global Tuberculosis Report. 2020.https://www.who.int/publications/i/item/9789240013131 (accessed 31 Jul 2021).

4 Snider B, Patel B, McBean E. Asymptomatic Cases, the Hidden Challenge in Predicting COVID-19 Caseload Increases. *Infect Dis Reports 2021, Vol 13, Pages 340-347* 2021;**13**:340–7. doi:10.3390/IDR13020033

5 TB Disease - Symptoms, treatment & prevention - TBFacts. https://tbfacts.org/tb-disease/ (accessed 28 Aug 2021).

6 Honeyborne I, McHugh TD, Phillips PPJ, *et al.* Molecular Bacterial Load Assay, a Culture-Free Biomarker for Rapid and Accurate Quantification of Sputum Mycobacterium tuberculosis Bacillary Load during Treatment. *J Clin Microbiol* 2011;**49**:3905. doi:10.1128/JCM.00547-11

7 UNIAIDS. UNAIDS Global AIDS Update. 2021. https://www.unaids.org/sites/default/files/media_asset/2021-global-aids-update_en.pdf (accessed 28 Oct 2021).

8 ZIMBABWE POPULATION CENSUS 2012 WOMEN AND MEN PROFILE SUMMARY REPORT. Published Online First: 2016.https://www.zimstat.co.zw/wp-content/uploads/publications/Social/Gender/Women-and-Men-Report-2012.pdf (accessed 15 Oct 2021).

9 Tanzania Census 2012 - Dashboard. http://dataforall.org/dashboard/tanzania/ (accessed 15 Oct 2021).

10 ZIMBABWE POPULATION-BASED HIV IMPACT ASSESSMENT ZIMPHIA 2020 KEY FINDINGS. https://phia.icap.columbia.edu/wp-content/uploads/2020/11/ZIMPHIA-2020-Summary-Sheet_Web.pdf (accessed 3 Sep 2021).

11 Xadreque H, Maunze A, Dade M, *et al.* IV RECENSEAMENTO GERAL DA POPULAÇÃO E HABITAÇÃO. 2017.http://www.ine.gov.mz/iv-rgph-2017/mocambique/censo-2017-brochura-dos-resultados-definitivos-do-iv-rgph-nacional.pdf (accessed 31 Jul 2021).

12 Population of Cities in Zimbabwe (2021). https://worldpopulationreview.com/countries/cities/zimbabwe (accessed 3 Aug 2021).

13 World atlas. https://knoema.com/atlas/United-Republic-of-Tanzania. (accessed 26 Jul 2021).

14 Prevalence of HIV, total (% of population ages 15-49) | Data. https://data.worldbank.org/indicator/SH.DYN.AIDS.ZS?locations=ZW (accessed 3 Aug 2021).

15 Incidence of tuberculosis (per 100,000 people) - Zimbabwe | Data. https://data.worldbank.org/indicator/SH.TBS.INCD?locations (accessed 3 Aug 2021).

16 Fox GJ, Nhung N V., Sy DN, *et al.* Household-Contact Investigation for Detection of Tuberculosis in Vietnam. *N Engl J Med* 2018;**378**:221–9. doi:10.1056/NEJMOA1700209

17 Qin ZZ, Ahmed S, Sarker MS, *et al.* Tuberculosis detection from chest x-rays for triaging in a high tuberculosis-burden setting: an evaluation of five artificial intelligence algorithms. *Lancet Digit Heal* 2021;**3**:e543–54. doi:10.1016/S2589-7500(21)00116-3

18 MacPherson P, Webb EL, Kamchedzera W, *et al.* Computer-aided X-ray screening for tuberculosis and HIV testing among adults with cough in Malawi (the PROSPECT study): A randomised trial and cost-effectiveness analysis. *PLoS Med* 2021;**18**:1–17. doi:10.1371/journal.pmed.1003752

19 Dorman S, Schumacher S, Alland D, *et al.* Xpert MTB/RIF Ultra for detection of Mycobacterium tuberculosis and rifampicin resistance: a prospective multicentre diagnostic accuracy study. *Lancet Infect Dis* 2018;**18**:76–84. doi:10.1016/S1473-

3099(17)30691-6

20 WHO Meeting Report of a Technical Expert Consultation: Non-inferiority analysis of Xpert MTB/RIF Ultra compared to Xpert MTB/RIF. Published Online First: 2017.http://apps.who.int/bookorders. (accessed 19 Oct 2021).

21 World Health Organization. WHO consolidated guidelines on tuberculosis: Module 2: Screening. 2021.https://www.who.int/publications/i/item/9789240022676 (accessed 9 Dec 2021).

22 Hamasur B, Bruchfeld J, van Helden P, *et al.* A sensitive urinary lipoarabinomannan test for tuberculosis. *PLoS One* 2015;**10**. doi:10.1371/JOURNAL.PONE.0123457

23 Portevin D, Moukambi F, Clowes P, *et al.* Assessment of the novel T-cell activation marker-tuberculosis assay for diagnosis of active tuberculosis in children: a prospective proof-of-concept study. *Lancet Infect Dis* 2014;**14**:931–8. doi:10.1016/S1473-3099(14)70884-9

24 Schuetz A, Haule A, Reither K, *et al.* Monitoring CD27 expression to evaluate Mycobacterium tuberculosis activity in HIV-1 infected individuals in vivo. *PLoS One* 2011;**6**. doi:10.1371/JOURNAL.PONE.0027284

25 Ahmed MIM, Ntinginya NE, Kibiki G, *et al.* Phenotypic Changes on Mycobacterium Tuberculosis-Specific CD4 T Cells as Surrogate Markers for Tuberculosis Treatment Efficacy. *Front Immunol* 2018;**9**:2247. doi:10.3389/FIMMU.2018.02247/BIBTEX

26 Gupta RK, Turner CT, Venturini C, *et al.* Concise whole blood transcriptional signatures for incipient tuberculosis: a systematic review and patient-level pooled meta-analysis. *Lancet Respir Med* 2020;**8**:395–406. doi:http://dx.doi.org/10.1016/S2213-2600%2819%2930282-6

27 Scriba TJ, Fiore-Gartland A, Penn-Nicholson A, *et al.* Biomarker-guided tuberculosis preventive therapy (CORTIS): a randomised controlled trial. *Lancet Infect Dis* 2021;**21**:354–65. doi:10.1016/S1473-3099(20)30914-2/ATTACHMENT/EB270186-669A-4F82-8A80-FB38FA1FB796/MMC1.PDF

28 Warsinske H, Rao A, Moreira F, *et al.* Assessment of Validity of a Blood-Based 3-Gene Signature Score for Progression and Diagnosis of Tuberculosis, Disease Severity, and Treatment Response. *JAMA Netw open* 2018;**1**:e183779. doi:10.1001/JAMANETWORKOPEN.2018.3779

29 Sweeney TE, Braviak L, Tato CM, *et al.* Genome-wide expression for diagnosis of pulmonary tuberculosis: a multicohort analysis. *Lancet Respir Med* 2016;**4**:213–24. doi:10.1016/S2213-2600(16)00048-5

30 Mendelson F, Griesel R, Tiffin N, *et al.* C-reactive protein and procalcitonin to discriminate between tuberculosis, Pneumocystis jirovecii pneumonia, and bacterial pneumonia in HIV-infected inpatients meeting WHO criteria for seriously ill: a prospective cohort study. *BMC Infect Dis* 2018;**18**. doi:10.1186/S12879-018-3303-6

31 Huang C, Lee L, Ho C, *et al.* High serum levels of procalcitonin and soluble TREM-1 correlated with poor prognosis in pulmonary tuberculosis. *J Infect* 2014;**68**:440–7. doi:10.1016/J.JINF.2013.12.012

32 Feng J, Su W, Pan S, *et al.* Role of TREM-1 in pulmonary tuberculosis patients-analysis of serum soluble TREM-1 levels. *Sci Rep* 2018;**8**. doi:10.1038/S41598-018-26478-2

33 Kumar NP, Velayutham B, Nair D, *et al.* Angiopoietins as biomarkers of disease severity and bacterial burden in pulmonary tuberculosis. *Int J Tuberc Lung Dis* 2017;**21**:93. doi:10.5588/IJTLD.16.0565

34 Kathamuthu G, Moideen K, Baskaran D, *et al.* Tuberculous lymphadenitis is associated with altered levels of circulating angiogenic factors. *Int J Tuberc Lung Dis* 2018;**22**:557–66. doi:10.5588/IJTLD.17.0609

35 Sivro A, McKinnon L, Yende-Zuma N, *et al.* Plasma Cytokine Predictors of Tuberculosis Recurrence in Antiretroviral-Treated Human Immunodeficiency Virus-infected Individuals from Durban, South Africa. *Clin Infect Dis* 2017;**65**:819–26. doi:10.1093/CID/CIX357

36 La Manna M, Orlando V, Li Donni P, *et al.* Identification of plasma biomarkers for discrimination between tuberculosis infection/disease and pulmonary non tuberculosis

For peer review only

disease. *PLoS One* 2018;**13**. doi:10.1371/JOURNAL.PONE.0192664

37 Sivro A, McKinnon L, Yende-Zuma N, *et al.* Plasma Cytokine Predictors of Tuberculosis Recurrence in Antiretroviral-Treated Human Immunodeficiency Virus-infected Individuals from Durban, South Africa. *Clin Infect Dis* 2017;**65**:819–26. doi:10.1093/CID/CIX357

38 Chegou NN, Sutherland JS, Malherbe S, *et al.* Diagnostic performance of a seven-marker serum protein biosignature for the diagnosis of active TB disease in African primary healthcare clinic attendees with signs and symptoms suggestive of TB. *Thorax* 2016;**71**:785–94. doi:10.1136/thoraxjnl-2015-207999

39 Collins GS, Reitsma JB, Altman DG, *et al.* Transparent reporting of a multivariable prediction model for individual prognosis or diagnosis (TRIPOD): The TRIPOD Statement. *BMC Med* 2015;**13**:1–10. doi:10.1186/S12916-014-0241-Z/TABLES/1

40 Trevethan R. Sensitivity, Specificity, and Predictive Values: Foundations, Pliabilities, and Pitfalls in Research and Practice. *Front public Heal* 2017;**5**. doi:10.3389/FPUBH.2017.00307

41 Agency ZS. QUALITY CONTROL MANUAL Provincial Report Harare Central Census Office. https://www.zimstat.co.zw/wp-content/uploads/publications/Population/population/Harare.pdf (accessed 3 Aug 2021).

42 Mozambique Population 2021 (Demographics, Maps, Graphs). https://worldpopulationreview.com/countries/mozambique-population (accessed 27 Jul 2021).

43 ZIMBABWE NATIONAL HIV/AIDS AND TUBERCULOSIS CONTROL PROGRAMMES. Republic of Zimbabwe NATIONAL HIV/AIDS AND TUBERCULOSIS CONTROL PROGRAMMES. https://www.who.int/hiv/pub/guidelines/zimbabwe.pdf (accessed 1 Sep 2021).

For peer review only

584 **Figure legend**

585 **Figure 1.** The ERASE-TB consortium

586 Abbreviations: ERASE-TB=Early Risk Assessment in TB contactS by new diagnostic tEsts

587

588 **Figure 2.** Location and characteristics of ERASE-TB study sites

589 Notes: The location of each study site is indicated by a red asterix. Source data used within

590 this figure are taken from the references [7,8,42,43,9–15,41].

591 Abbreviations: ERASE=Early Risk Assessment in TB contactS by new diagnostic tEsts;

592 TB=tuberculosis; HIV=human immunodeficiency virus

593

594 **Figure 3.** Eligibility criteria and schedules of events for index cases and household contacts

595 Notes: **A**=depending on the time point of study enrollment and consequently on the duration

596 available for follow-up, i.e. 18 or 24 months, the follow-up visit at 24 months ±30 days may be

597 conditional; **B**=the follow-up visit by phone may be conducted after the last scheduled follow-

598 up visit at 18 months ±30 days or 24 months ±30 days to assess whether symptoms suggestive

599 of TB have occurred, TB diagnosis has been made or anti-TB treatment has been initiated;

600 **C**=unwell visits by phone or on-site may be conducted between scheduled follow-up visits if a

601 participant presents at a recruitment healthcare facility with signs and symptoms suggestive of

602 TB; **D**=coached spontaneous or induced sputum collection for storage at scheduled follow-up

603 visit at 18 months ±30 days or 24 months ±30 days, and for repetition of HIV testing if tested

604 negative at baseline; **E**=coached spontaneous or induced sputum collection upon the decision

605 of the investigating team for testing by Xpert MTB/RIF Ultra if participant presents with signs

606 and symptoms suggestive of TB; **F**=coached spontaneous or induced sputum collection in

607 case of Xpert MTB/RIF Ultra positivity or strong clinical suspicion of TB for repetition of the

608 Xpert MTB/RIF Ultra; **G**=in case of HIV positivity to be followed by the assessment of CD4

609 counts; **H**=CXR to be conducted at an unscheduled on-site unwell visit upon the decision of

610 the investigating team depending on the nature of symptoms reported, and the time elapsed

611 since the last CXR including its findings; **I**=not to be conducted among pregnant women;

For peer review only

J=stored venous blood includes 6mL EDTA blood for whole blood and plasma, 4mL serum and 2.5mL PAXgene blood, all samples will be deep frozen for retrospective testing using new diagnostics as described in text; **K**=in case the evaluation of symptoms of a participant unable to present at a recruitment healthcare facility is required an unscheduled on-site or home visit will be arranged by phone, the resolution of symptoms can alternatively be addressed by phone; **L**=collection of PBMC at baseline and follow-up visit at 6 months ±30 days is optional, thus will not be performed at each participating site and for each participant; **M**=in case the evaluation of symptoms of a participant unable to present at a recruitment healthcare facility is required or doubtful if required an unscheduled unwell visit by phone will be arranged, the resolution of symptoms can alternatively be addressed by phone; **N**=spirometry and/or diabetes (HbA1c) will be performed at scheduled follow-up visits at 6 months ±30 days, 12 months ±30 days and 18 or 24 months ±30 days if required or not performed at baseline, anaemia (Hb) will be performed at baseline and scheduled follow-up visits at 6 months ±30 days, 12 months ±30 days and 18 or 24 months ±30 days if possible; **O**=blood pressure measurement will be performed at baseline and scheduled follow-up visits at 6 months ±30 days, 12 months ±30 days and 18 or 24 months ±30 days; **P**=WGS to be performed once *Mtb* infection is confirmed and an isolate could be recovered.

Abbreviations: TB=tuberculosis; WHO=World Health Organization; HIV=human immunodeficiency virus; WGS=whole genome sequencing; MBLA=molecular bacterial load assay; CXR=chest radiograph; IGRA=interferon gamma release assay; PBMC=peripheral blood mononuclear cell; TAM-TB=T- cell activation marker tuberculosis; MTB=*Mycobacterium tuberculosis*; RIF=rifampicin, Hb=haemoglobin; HbA1c=glycated hemoglobin; LAM=lioparabinomannan; CD4=cluster of differentiation 4

Figure 4. Study design

Abbreviations: HHC=household contact; TB=tuberculosis; IC=index case; WHO=World Health Organization; CXR= chest radiograph; SS=symptom score; MTB=*Mycobacterium tuberculosis*; RIF=rifampicin; pos=positive; CT=cpmputer tomography; FU=follow-up

For peer review only

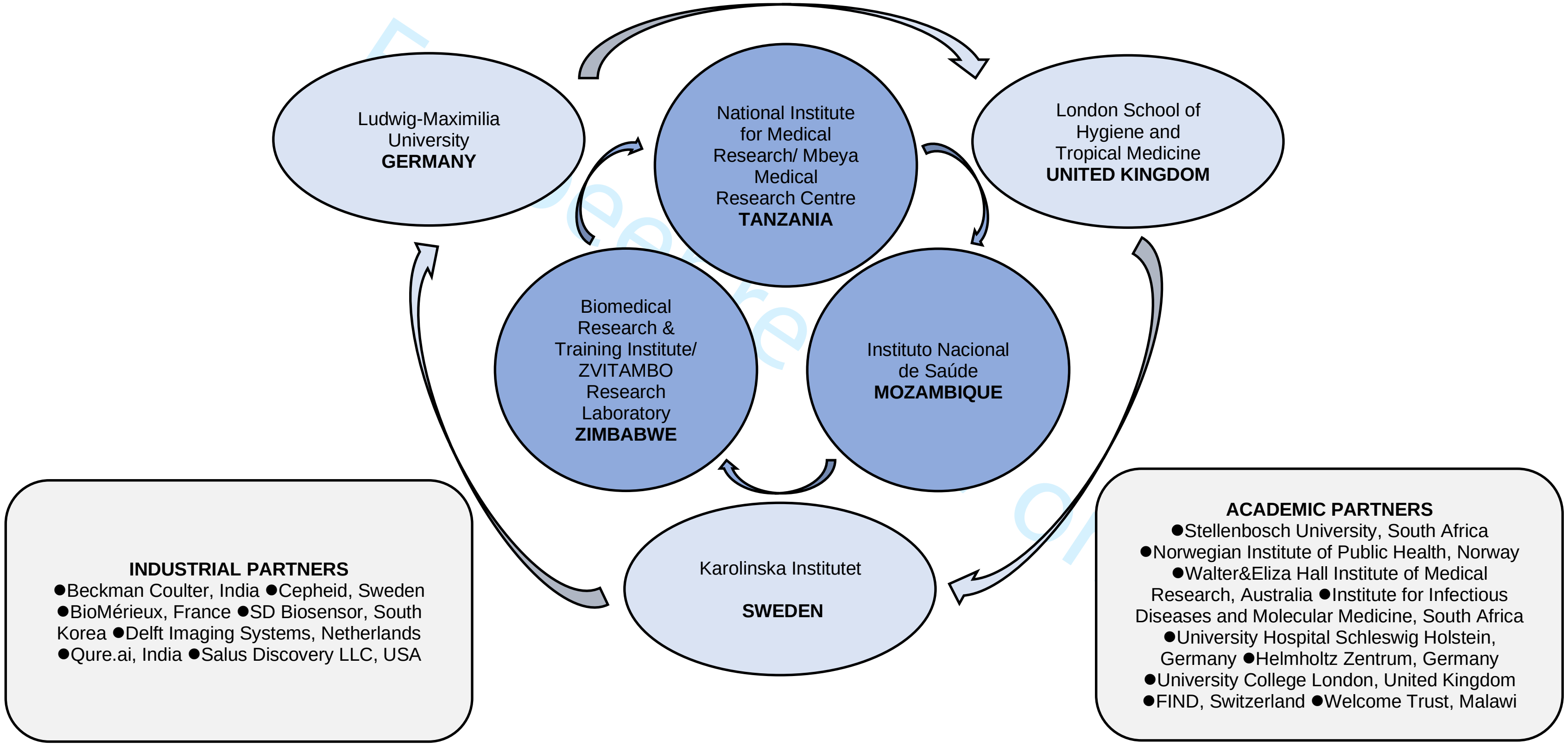

Figure 1. The ERASE-TB consortium

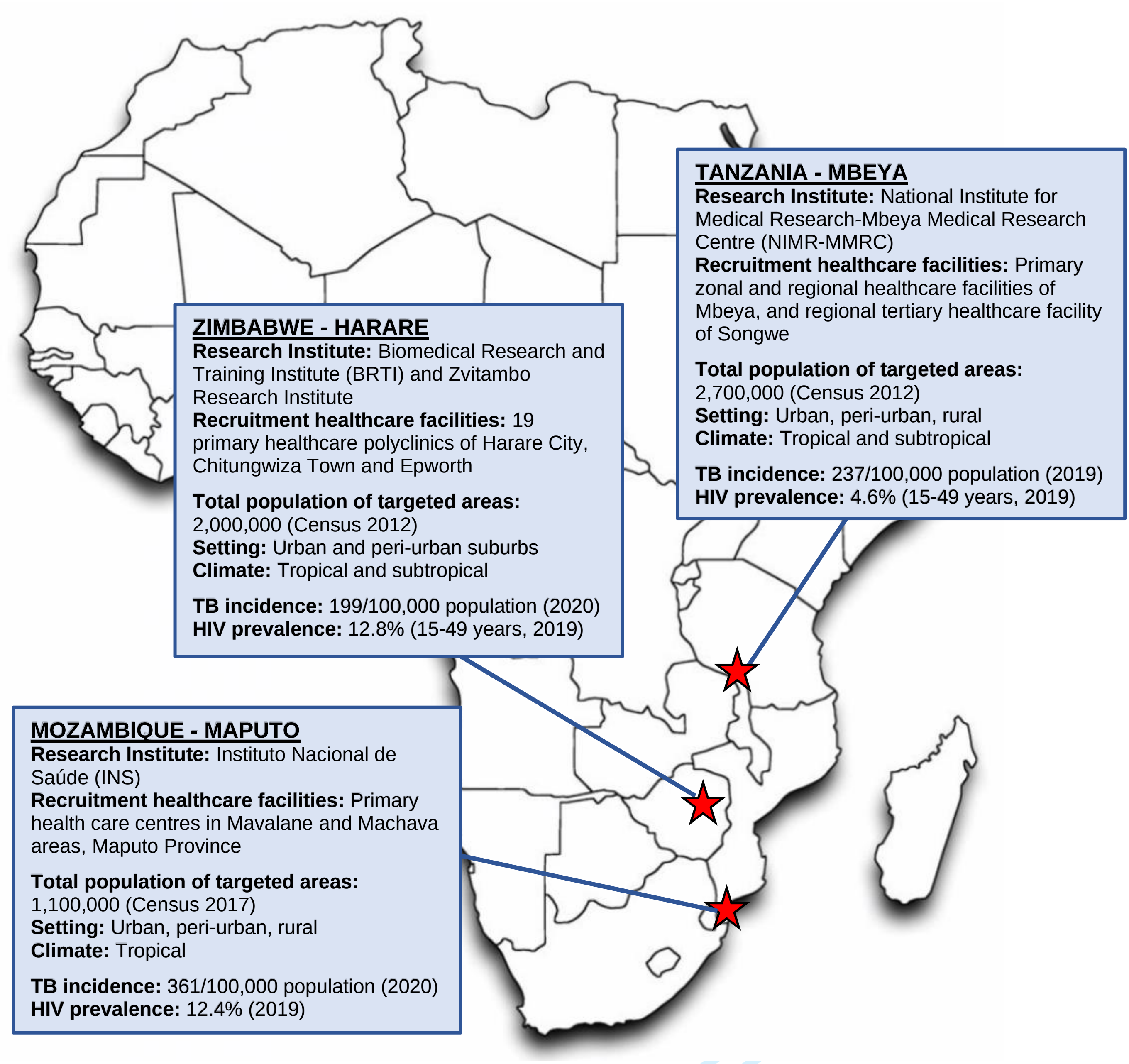

Figure 2. Location and characteristics of ERASE-TB study sites

INDEX CASE

INCLUSION CRITERIA
- Aged ≥18 years
- Living with at least another person aged ≥10 years in the same household with a firm unchanged address for 6 months
- Pulmonary TB diagnosis within 4 weeks prior to enrolment
- Spontaneous sputum production
- Positive sputum smear, i.e. >+2 positivity by Ziehl-Neelsen stain or Auramine-O stain OR ≥+1 positivity on WHO symptom scale OR ≥medium positivity by GeneXpert (if no microscopy performed)
- <7 daily doses of anti-TB treatment since diagnosis
- Voluntary written informed consent obtained

IN

EVENTS AT BASELINE/SCREENING VISIT
- Administration of socio-demographic questionnaire
- Assessment of vital signs, medical history
- Digital CXR (if available)[I]

Specimen investigations
- Sputum: culture, storage, WGS, MBLA

NO FOLLOW-UP

HOUSEHOLD CONTACT

EXCLUSION CRITERIA
- Treatment for TB within the past 30 days OR on current TB treatment OR on current preventive TB therapy (if HIV negative) at the tim of enrolment

OUT

INCLUSION CRITERIA
- Aged ≥10 years
- Substantial exposure, i.e. spending ≥3 nights/week over the past 4 weeks in the same household as the index case,
- Voluntary written informed consent or parent/guardian consent & assent in case of minors obtained

IN

EVENTS AT BASELINE/ ±14days of SCREENING VISIT
- Administration of socio-demographic questionnaire
- Assessment vital signs, medical history
- Screening for diabetes (HbA1c)[N], high blood pressure[O], anaemia (Hb)[N], WHO TB symptoms
- Digital CXR[I], spirometry[N]

Specimen investigations
- Sputum: Xpert MTB/RIF Ultra[E], culture[F], storage, WGS[P]
- Blood: HIV/CD4 counts[G], Cepheid host response, TAM-TB, IGRA SD Biosensor, PBMC[L], storage[J]
- Urine: dipstick/LAM, storage

FOLLOW-UP

EVENTS AT UNSCHEDULED UNWELL VISIT(S) (phone/on-site)[C,K, M]
- Assessment of vital signs, medical history
- Screening for high blood pressure, WHO TB symptoms,
- Digital CRX[H,I]

Specimen investigations
- Sputum: Xpert MTB/RIF Ultra, culture, storage, WGS[P]
- Blood: Cepheid host response, TAM-TB, storage[J]
- Urine: dipstick/LAM, storage

EVENTS AT UN-SCHEDULED FOLLOW-UP VISIT(S)[B] (phone)
- Assessment of medical history
- Screening for WHO TB symptoms

Specimen investigations
- None

EVENTS AT 6months ±30days/ 12months ±30days/ 18months ±30days/ 24months ±30days[A] FOLLOW-UP VISITS
- Assessment of vital signs, medical history
- Screening for high blood pressure, WHO TB symptoms
- Digital CXR[I], spirometry[N]

Specimen investigations
- Sputum: Xpert MTB/RIF Ultra[E], culture[F], storage[D], WGS[P]
- Blood: Cepheid host response, TAM-TB, PBMC[L], storage
- Urine: dipstick/LAM, storage[J]

OUT

OUT TB+

Optional

Optional

For peer review only - http://bmjopen.bmj.com/site/about/guidelines.xhtml

Figure 3. Eligibility criteria and schedules of events for index cases and household contacts

For peer review only

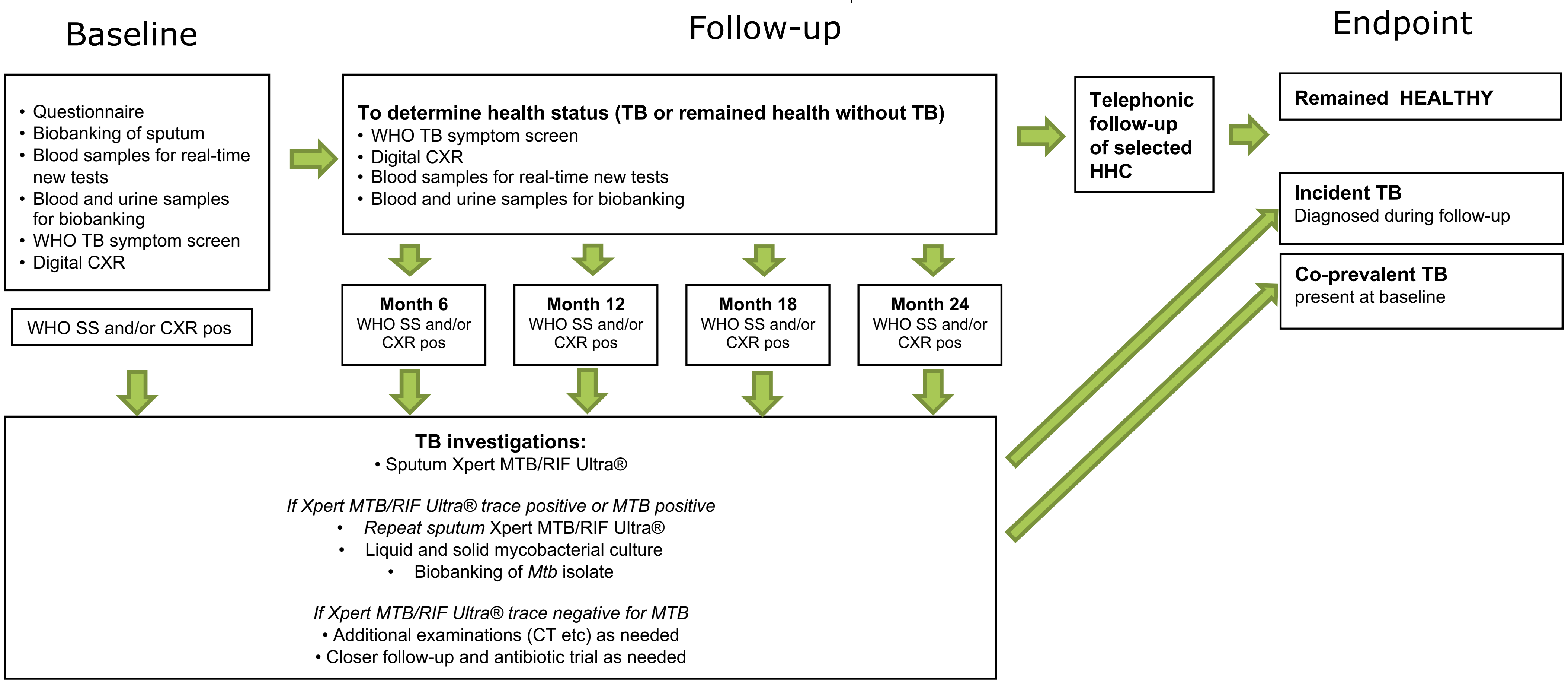

Figure 4. Study design.

Section & Topic	No	Item	Reported on page #
**TITLE OR ABSTRACT**			
	**1**	Identification as a study of diagnostic accuracy using at least one measure of accuracy (such as sensitivity, specificity, predictive values, or AUC)	2
**ABSTRACT**			
	**2**	Structured summary of study design, methods, results, and conclusions (for specific guidance, see STARD for Abstracts)	2-3
**INTRODUCTION**			
	**3**	Scientific and clinical background, including the intended use and clinical role of the index test	5-6
	**4**	Study objectives and hypotheses	6
**METHODS**			
Study design	**5**	Whether data collection was planned before the index test and reference standard were performed (prospective study) or after (retrospective study)	6-7
Participants	**6**	Eligibility criteria	7 (Figure 3)
	**7**	On what basis potentially eligible participants were identified (such as symptoms, results from previous tests, inclusion in registry)	7 (Figure 3)
	**8**	Where and when potentially eligible participants were identified (setting, location and dates)	7
	**9**	Whether participants formed a consecutive, random or convenience series	7, 11
Test methods	**10a**	Index test, in sufficient detail to allow replication	8-10, 11-13
	**10b**	Reference standard, in sufficient detail to allow replication	8-10, 11-13
	**11**	Rationale for choosing the reference standard (if alternatives exist)	8-10, 11-13
	**12a**	Definition of and rationale for test positivity cut-offs or result categories of the index test, distinguishing pre-specified from exploratory	13-14
	**12b**	Definition of and rationale for test positivity cut-offs or result categories of the reference standard, distinguishing pre-specified from exploratory	13-14
	**13a**	Whether clinical information and reference standard results were available to the performers/readers of the index test	13-14
	**13b**	Whether clinical information and index test results were available to the assessors of the reference standard	13-14
Analysis	**14**	Methods for estimating or comparing measures of diagnostic accuracy	13-14
	**15**	How indeterminate index test or reference standard results were handled	13-14
	**16**	How missing data on the index test and reference standard were handled	14
	**17**	Any analyses of variability in diagnostic accuracy, distinguishing pre-specified from exploratory	13-14
	**18**	Intended sample size and how it was determined	11
**RESULTS**			
Participants	**19**	Flow of participants, using a diagram	8-10 (Figure 3)
	**20**	Baseline demographic and clinical characteristics of participants	8-10 (Figure 3)
	**21a**	Distribution of severity of disease in those with the target condition	
	**21b**	Distribution of alternative diagnoses in those without the target condition	
	**22**	Time interval and any clinical interventions between index test and reference standard	
Test results	**23**	Cross-tabulation of the index test results (or their distribution) by the results of the reference standard	
	**24**	Estimates of diagnostic accuracy and their precision (such as 95% confidence intervals)	13-14
	**25**	Any adverse events from performing the index test or the reference standard	
**DISCUSSION**			
	**26**	Study limitations, including sources of potential bias, statistical uncertainty, and generalisability	3
	**27**	Implications for practice, including the intended use and clinical role of the index test	5-6
**OTHER INFORMATION**			
	**28**	Registration number and name of registry	2
	**29**	Where the full study protocol can be accessed	
	**30**	Sources of funding and other support; role of funders	16

For peer review only - http://bmjopen.bmj.com/site/about/guidelines.xhtml

STARD 2015

AIM

STARD stands for "Standards for Reporting Diagnostic accuracy studies". This list of items was developed to contribute to the completeness and transparency of reporting of diagnostic accuracy studies. Authors can use the list to write informative study reports. Editors and peer-reviewers can use it to evaluate whether the information has been included in manuscripts submitted for publication.

EXPLANATION

A **diagnostic accuracy study** evaluates the ability of one or more medical tests to correctly classify study participants as having a **target condition.** This can be a disease, a disease stage, response or benefit from therapy, or an event or condition in the future. A medical test can be an imaging procedure, a laboratory test, elements from history and physical examination, a combination of these, or any other method for collecting information about the current health status of a patient.

The test whose accuracy is evaluated is called **index test.** A study can evaluate the accuracy of one or more index tests. Evaluating the ability of a medical test to correctly classify patients is typically done by comparing the distribution of the index test results with those of the **reference standard**. The reference standard is the best available method for establishing the presence or absence of the target condition. An accuracy study can rely on one or more reference standards.

If test results are categorized as either positive or negative, the cross-tabulation of the index test results against those of the reference standard can be used to estimate the **sensitivity** of the index test (the proportion of participants *with* the target condition who have a positive index test), and its **specificity** (the proportion *without* the target condition who have a negative index test). From this cross-tabulation (sometimes referred to as the contingency or "2x2" table), several other accuracy statistics can be estimated, such as the positive and negative **predictive values** of the test. Confidence intervals around estimates of accuracy can then be calculated to quantify the statistical **precision** of the measurements.

If the index test results can take more than two values, categorization of test results as positive or negative requires a **test positivity cut-off**. When multiple such cut-offs can be defined, authors can report a receiver operating characteristic (ROC) curve which graphically represents the combination of sensitivity and specificity for each possible test positivity cut-off. The **area under the ROC curve** informs in a single numerical value about the overall diagnostic accuracy of the index test.

The **intended use** of a medical test can be diagnosis, screening, staging, monitoring, surveillance, prediction or prognosis. The **clinical role** of a test explains its position relative to existing tests in the clinical pathway. A replacement test, for example, replaces an existing test. A triage test is used before an existing test; an add-on test is used after an existing test.

Besides diagnostic accuracy, several other outcomes and statistics may be relevant in the evaluation of medical tests. Medical tests can also be used to classify patients for purposes other than diagnosis, such as staging or prognosis. The STARD list was not explicitly developed for these other outcomes, statistics, and study types, although most STARD items would still apply.

DEVELOPMENT

This STARD list was released in 2015. The 30 items were identified by an international expert group of methodologists, researchers, and editors. The guiding principle in the development of STARD was to select items that, when reported, would help readers to judge the potential for bias in the study, to appraise the applicability of the study findings and the validity of conclusions and recommendations. The list represents an update of the first version, which was published in 2003.

More information can be found on http://www.equator-network.org/reporting-guidelines/stard.

For peer review only

CC BY SA

BMJ Open

Early risk assessment in pediatric and adult household contacts of confirmed tuberculosis cases by novel diagnostic tests (ERASE-TB): protocol for a prospective, non-interventional, longitudinal, multi-country cohort study

Journal:	*BMJ Open*
Manuscript ID	bmjopen-2022-060985.R2
Article Type:	Protocol
Date Submitted by the Author:	23-Jun-2022
Complete List of Authors:	Marambire, Edson; Biomedical Research and Training Institute,<br>Banze, Denise; Instituto Nacional de Saúde<br>Mfinanga, Alfred; Mbeya Medical Research Centre<br>Mutsvangwa, Junior; Biomedical Research and Training Institute<br>Mbunda, Theodora ; NIMR-Mbeya Medical Research Programme<br>Ntinginya, Nyanda; 6. National Institute of Medical Research-Mbeya Medical Research Centre<br>Celso, Khosa; Instituto Nacional de Saúde<br>Kallenius, Gunilla; Karolinska Institutet<br>Calderwood, Claire J.; London School of Hygiene and Tropical Medicine Faculty of Infectious and Tropical Diseases<br>Geldmacher, Christof; University Hospital, Division of Infectious Diseases and Tropical Medicine, LMU Munich ; German Center for Infection Research, Partner site Munich<br>Held, Kathrin; University Hospital, Division of Infectious and Tropical Medicine, LMU Munich ; German Center for Infection Research, Partner site Munich<br>Appalarowthu, Tejaswi; University Hospital, Division of Infectious Diseases and Tropical Medicine, LMU Munich; German Center for Infection Research, Partner site Munich<br>Rieß, Friedrich; University Hospital, Division of Infectious Diseases and Tropical Medicine, LMU Munich; German Center for Infection Research, Partner site Munich<br>Panzner, Ursula; University Hospital, Division of Infectious Diseases and Tropical Medicine, LMU Munich; German Center for Infection Research, Partner site Munich<br>Heinrich, Norbert; University Hospital, Division of Infectious Diseases and Tropical Medicine, LMU Munich; German Center for Infection Research, Partner site Munich<br>Kranzer, Katharina; London School of Hygiene and Tropical Medicine Faculty of Infectious and Tropical Diseases; Biomedical Research and Training Institute
**Primary Subject Heading**:	Respiratory medicine
Secondary Subject Heading:	Diagnostics, Infectious diseases

Keywords:	Tuberculosis < INFECTIOUS DISEASES, Diagnostic microbiology < INFECTIOUS DISEASES, RESPIRATORY MEDICINE (see Thoracic Medicine)

SCHOLARONE™
Manuscripts

Early risk assessment in pediatric and adult household contacts of confirmed tuberculosis cases by novel diagnostic tests (ERASE-TB): protocol for a prospective, non-interventional, longitudinal, multi-country cohort study

Edson Tawanda Marambire[1*], Denise Banze[2$], Alfred Mfinanga[3$], Junior Mutsvangwa[1], Theodora Mbunda[3], Elias N. Nyanda[3], Khosa Celso[2], Gunilla Kallenius[4], Claire J. Calderwood[5], Christof Geldmacher[6,7], Kathrin Held[6,7], Tejaswi Appalarowthu[6,7], Friedrich Rieß[6,7], Ursula Panzner[6,7], Norbert Heinrich[6,7#], Katharina Kranzer[1,5,6#], on behalf of the ERASE-TB Consortium

[1]Biomedical Research and Training Institute, Harare, Zimbabwe

[2]Instituto Nacional de Saúde, Marracuene, Mozambique

[3]National Institute for Medical Research - Mbeya Medical Research Centre, Mbeya, Tanzania

[4]Karolinska Institute, Stockholm, Sweden

[5]Faculty of Infectious and Tropical Diseases and Tuberculosis Centre, London School of Hygiene & Tropical Medicine, London, UK

[6]Division of Infectious Diseases and Tropical Medicine, University Hospital, LMU Munich, Munich, Germany

[7]German Center for Infection Research (DZIF), Partner site Munich, Munich, Germany

*Corresponding author: Edson Tawanda Marambire; 8 Rose Avenue, Belgravia, Harare, Zimbabwe; e-mail: edsonmarambire@gmail.com; phone: +263772286311

$ Contributed equally

# Contributed equally

Word count: 3,668

Keywords: *Mycobacterium tuberculosis*, diagnostics, cohort study, household contacts, WHO END TB strategy, ERASE-TB

For peer review only

ABSTRACT

Introduction

The World Health Organization (WHO) End-TB Strategy calls for the development of novel diagnostics to detect tuberculosis earlier and more accurately. Better diagnostics, together with tools to predict disease progression are critical for achieving WHO END-TB targets. The Early Risk Assessment in TB contactS by new diagnostic tEsts (ERASE-TB) study aims to evaluate novel diagnostics and testing algorithms for early tuberculosis diagnosis and accurate prediction of disease progression among household contacts exposed to confirmed index cases in Mozambique, Tanzania and Zimbabwe.

Methods and analysis

A total of 2,100 household contacts (HHCs) (aged ≥10 years) of adults with microbiologically-confirmed pulmonary tuberculosis will be recruited and followed up at 6-month intervals for 18 to 24 months. At each time-point a WHO symptom screen and digital chest-radiograph (dCXR) will be performed, and blood and urine samples collected. Individuals screening positive (WHO symptom screen or dCXR) will be requested to provide sputum for Xpert MTB/Rif Ultra. At baseline, HHCs will also be screened for HIV, diabetes (HbA1c), chronic lung disease (spirometry), hypertension and anaemia. Study outcomes will be co-prevalent tuberculosis (diagnosed at enrollment), incident tuberculosis (diagnosed during follow-up) or no tuberculosis at completion of follow up. Novel diagnostics will be validated using fresh and biobanked samples with a nested case control design. Cases are defined as HHCs diagnosed with tuberculosis (for early diagnosis) or with incident tuberculosis (for prediction of progression) and will be matched by age, sex and country to HHCs who remain healthy (controls). Statistical analyses will include assessment of diagnostic accuracy by constructing receiver operating curves and calculation of sensitivity and specificity.

Ethics and dissemination

For peer review only

ERASE-TB has been approved by regulatory and ethical committees in each African country and by each partner organisation. Consent, with additional assent for participants <18 years, is voluntary. Attestation by impartial witnesses is sought in case of illiteracy. Confidentiality of participants is being maintained throughout. Study findings will be presented at scientific conferences and published in peer-reviewed international journals.

Study registration number

NCT04781257

For peer review only

Strengths and limitations of this study

- Recruitment of highly infectious index cases aimed at maximising the number of tuberculosis (TB) diagnoses in the household contact (HHCs) cohort.
- Sequencing of *Mycobacterium tuberculosis* isolates from both index cases and HHCs allows confirmation of household transmission and thus determination of timing of the transmission event; resulting in more precise estimates of new test sensitivity compared to population-based cohorts with unknown timing of infection.
- Large sample size across three southern African countries with high HIV prevalence; including adolescents will ensure study findings are generalisable to the clinically relevant population at high risk of TB compared to studies focused on adults only.
- Despite the large cohort of HHCs, the number of diagnosed TB cases will be small, limiting the power of the study and sub-group analyses such as by age and HIV status.
- Geographically limited to sub-Saharan Africa, therefore results may not be generalisable to other populations, including those with lower HIV prevalence such as in South-East Asia or the Americas.

For peer review only

INTRODUCTION

Tuberculosis (TB) remains a leading global public health problem, with an estimated 10 million new cases and 1.5 million deaths globally in 2020 [1]. In 2014, the World Health Assembly approved the World Health Organization (WHO) End-TB Strategy, aiming for a 90% reduction in TB incidence and 95% reduction in TB deaths by 2035 [2]. However, in 2019, three million TB cases ('the missing millions') remained undiagnosed and untreated globally, resulting in potentially avoidable morbidity, mortality and onward transmission. The Covid-19 pandemic has resulted in a large decrease in the number of people newly diagnosed with TB and reported. This has increased the diagnostic gap by a further 1.3 million, resulting in an estimated 4.2 million undiagnosed TB cases in 2020 [3]. Also, for the first time in a decade TB deaths have risen, from an estimated 1.4 million in 2019 to 1.5 million in 2020, as a result of reduced access to and provision of essential TB services including diagnostics during the Covid-19 pandemic.

Without an efficacious and safe vaccine, early detection and containment are the main tools to interrupt transmission and successfully control TB. Similar to SARS-CoV2, asymptomatic spreading of *M.tuberculosis* and subclinical but infectious disease states are a major concern in the control of airborne infectious diseases [4]. Early and accurate identification of persons with TB, combined with identification of those at risk of progression to TB and provision of targeted preventive treatment are critical to reducing TB-associated morbidity and mortality, and preventing onward transmission.

Currently available diagnostics such as sputum microscopy, mycobacterial culture and nucleic acid amplification tests are based on direct pathogen detection, thus requiring a high mycobacterial load; they therefore predominately target advanced TB when onward transmission and significant lung damage has occurred [5,6]. Further, for many patients with minimal or no symptoms, expectoration of high-quality sputum specimens remains

For peer review only

challenging, limiting the accuracy of sputum-based tests. The same holds true for young children and people living with HIV.

The **E**arly **R**isk **A**ssessment in TB Contacts by new diagno**S**tic t**E**sts (ERASE-TB) study aims to fill this diagnostic gap by evaluating new sputum and non-sputum-based TB diagnostics for early TB detection (before onward transmission occurs), as well as tools for more accurate prediction of TB progression to allow for targeted preventive therapy.

METHODS AND ANALYSES

Study objectives

ERASE-TB's primary objectives are (I) to determine the sensitivity and specificity of novel diagnostics to detect TB, in particular asymptomatic or minimally symptomatic TB; (II) to evaluate novel diagnostics for detection of likely TB progression; and (III) to enhance the performance of novel diagnostics by simulating testing algorithms coupled with individual risk estimates from a mathematical model. The secondary objectives are (I) to determine the TB prevalence among household contacts (HHCs) of infectious TB index cases (ICs) at baseline and during a 18-24 months follow-up; (II) to establish a biorepository of cryopreserved specimens from HHCs for future development and validation of diagnostic tests; and (III) to assess the association of selected chronic disease conditions and TB among HHCs.

Study endpoints

The study's primary endpoints are the presence or development of TB among HHCs with the following possible scenarios of (I) prevalent symptomatic TB at baseline, (II) incident TB during follow-up, and (III) remained healthy until study completion. An endpoint review committee will review the data and case classification before finalization.

Through the sequencing of *Mycobacterium tuberculosis* (*Mtb*) isolates, cases of co-prevalent or incident TB will be classified either as secondary, infected by the source case – the timepoint

For peer review only

of infection will be known; or as infected by another, unknown source of infection, with an unknown timepoint of infection.

Recruitment sites

Recruitment of ICs and HHCs at selected primary healthcare facilities and communities has commenced in Harare, Zimbabwe in March 2021, Maputo, Mozambique in August 2021, and Mbeya, Tanzania in September 2021. Partners of the ERASE-TB consortium are illustrated in Figure 1. All three countries have a high TB incidence ranging from 100 to 499/100,000 population [1] and HIV prevalence among adults aged 15 years and older of 5% to 20% [7]. The African research institutions have established collaborations with their respective National Tuberculosis Programs ensuring referral and approproate follow-up of TB patients. Figure 2 illustrates the geographic location of research institutions, healthcare facilities where recruitment is taking place, demographic characteristics of study populations, and estimates on TB incidence and HIV prevalence [8–15].

Study design

ERASE-TB is a non-interventional, longitudinal, prospective cohort study among HHCs aged ≥10 years exposed to highly infectious pulmonary TB ICs aged ≥18 years. Eligibility criteria are detailed in Figure 3 and the study design is shown in Figure 4. TB ICs are eligible if the bacterial load in their sputum is at least at the “medium” level according to Xpert MTB/RIF or Xpert MTB/RIF Ultra, and they have received less than seven daily doses of anti-TB treatment before enrollment. This maximises the likelihood of culturing and storing *Mtb* isolates. The total study duration will be 36 months. This includes 12-months enrollment of ICs and HHCs, and 18- to 24-months follow-ups of HHCs. Follow-up ends when a HHCs withdraws from the study, is lost to follow-up, dies, or is diagnosed with TB and referred for treatment. Scheduled or unscheduled unwell visits can be conducted physically and/or telephonically in case of abnormal finding e.g. by abnormal dCXR, or when a participant feels unwell inbetween scheduled follow-up visits.

For peer review only

Procedures

TB index cases

Following informed consent obtained, a questionnaire is administered to collect socio-demographic information, TB risk factors, and the medical history of TB, HIV and other diseases. Two spontaneous sputum samples are obtained, of which one is for mycobacterial culturing and one for storage for performing retrospectively Molecular Bacterial Load Assay (MBLA) to quantify viable *Mtb* by 16S rRNA [6]; an alternative means to quantify expectorated bacterial load for an estimate of infectiousness. Both liquid and solid mycobacterial cultures are performed on decontaminated sputum samples, with all *Mtb* isolates stored at -80 degrees for future DNA extraction and whole genome sequencing. A questionnaire on symptom duration and TB risk factors is also administered.

Household key informant

At baseline, a household key informant (either the TB index case or one of the household contacts) is identified and asked to answer questions of a household questionnaire that collects socio-economic elements like structure of the house or flat, income and household assets, and covariates possibly associated with risk of TB infection, e.g., windows/air exchange, presence of comorbid conditions, and risk factors like the source of cooking energy, and properties of the household kitchen.

Household contacts

Informed consent is obtained from all eligible adult HHCs. For HHC <18 years of age, the guardian is asked to provide informed consent, with assent also sought from children dependent on local guidance. At baseline a questionnaire is administered collecting information on socioeconomic and demographic characteristics, past medical history of TB, HIV and other diseases, exposure risk factors, smoking and alcohol history. The physical examination includes height, weight, mid-upper arm circumference and blood pressure

For peer review only

measurement. In addition, all HHCs are offered free HIV testing according to the National Guidelines. All people with confirmed HIV infection will have CD4 counts performed and be referred for TB preventive therapy. Those not yet on antiretroviral therapy (ART) and those who interrupted ART are referred for ART at local services.

Point of care HbA1c (A1cCare, SD Biosensor, Gyeonggi-do, Republic of Korea) and haemoglobin (Hemocue 301+, Hemocue, Angelholm, Sweden) tests and spirometry (including pre- and post-bronchodilation with inhaled salbutamol) are performed at baseline or the six-month visit. HHCs who did not take up HIV testing or other screening at baseline are offered these tests at each study visit. Any HHCs with test results requiring treatment or further investigations are referred for respective services.

HHCs are screened for TB using the WHO symptom questionnaire and a digital chest-radiograph (dCXR), reviewed by a clinical officer. dCXRs are not performed in pregnant HHCs. HHCs with a positive WHO symptom screening and abnormal dCXR are asked to provide sputum samples for TB investigations i.e., for GeneXpert and mycobacterial culture. Those with negative symptom screen and normal dCXR are asked to provide a spontaneous sputum sample for storage (with sputum induction performed if required).

At baseline, urine, serum, plasma, whole blood (native, and with RNA preservation in PAXgene® tubes [BD Biosciences, NJ, USA]) are stored. A finger-prick sample is taken and investigated using the Xpert TB Host Response RUO Prototype cartridge (Cepheid, Sunnyvale, CA, USA). T-cell Activation Marker Tuberculosis (TAM-TB) assay and Interferon Gamma Release Assay (IGRA; STANDARDTM F TB-Feron FIA (IFN-gamma; SD Biosensor, Republic of Korea), are performed on fresh venous blood. In Tanzania and Mozambique, storage of peripheral blood mononuclear cells for later characterization of the TB-specific immune response is also performed.

For peer review only

Procedures for follow-up and unwell visits are similar to those at baseline. Measurement of HIV status, haemoglobin, HbA1c, spirometry, CD4 count and IGRA testing are not performed at follow-up visits, unless not done previously. At the last scheduled visit all HHCs not known to have HIV are re-offered HIV testing and a spontaneous or induced sputum sample is stored for all participants.

Household contacts screening positive for TB symptoms and/or with a DCXR suggestive of TB

HHCs screening positive for symptoms and/or those with DCXRs suggestive of TB are asked for a sputum sample, which is investigated using Xpert MTB/RIF Ultra (Cepheid). If this sample is positive for *Mtb* (including a trace result), a minimum of two additional sputum samples are investigated, following decontamination, with Xpert MTB/RIF Ultra, solid and liquid culture. Isolates stored from these cultures will be sequenced for matching with the IC isolates in order to verify intra-household transmission. Sputum induction is performed for those unable to provide a spontaneous sputum sample. HHCs with microbiologically confirmed TB are referred for TB treatment to the National TB Programme.

Patient and public involvement

The ERASE-TB study sites have established Community Advisory Boards, which are voices of communities, people affected, and study participants, providing a strategic link between the communities and the study team. Community Advisory Boards meet regularly and provide feedback on design, procedures and conduct of the study. They will also be closely involved in the dissemination of study results. In addition to the Community Advisory Boards, each study site conducts community engagement activities focused on young people with the aim to foster interst in science and research, specififcally in the field of respiratory diseases/illness. This includes close partnership with schools and universities. Furthermore, planned qualitative research will specifically aim to understand the perceptions of HHCs with regards to TB diagnostics and screening.

For peer review only

Sample size

An estimated 800 to 900 TB-confirmed ICs are required for the subsequent enrollment of an anticipated 2,100 HHCs, i.e., 700 HHCs per country. Loss to follow-up of HHCs is estimated to be 10%. A total of 64 HHCs (3%) are estimated to be diagnosed with TB during the study period, based on previous active case finding studies among HHCs [16]. Validation for subclinical and early TB will include incident (n=49) and co-prevalent TB cases (n=15). Validation for detection of incipient *M.tb* infection will include samples of participants with incident TB (n=49) matched 1:4 to samples of participants without TB (n=196). For tests diagnosing incipient *M.tb* infection sensitivities of 73% and 82% would be detected with a precision of 59-85% and 68-91% respectively. For specifcities of 92% and 94% the confidence intervals would be 87-95% and 90-97%.

Novel test candidates

A range of novel test candidates targeted at pathogen detection or identification of host responses to *Mtb* are being applied, either in real-time (for all participants) or retrospectively (in a case-control design). Whilst a number of novel test candidates have been pre-specified, the ERASE-TB biobanking processes allow for addition of further candidate tests to be evaluated on stored samples as they become available.

DCXRs offer good sensitivity for diagnosis of pulmonary TB. However, high inter- and intra-investigator variability, and lack of trained interpreters present a barrier to implementation in many high-TB burden settings. Computer-aided interpretation systems, such as CAD4TB (Delft Imaging, Hertogenbosch, Netherlands) and qXR (Qure.ai, India) may increase image-reading capacity, with good performance, and serve, therefore, as a systematic screening tool to identify individuals in need of confirmatory TB tests [17,18].

For peer review only

Xpert MTB/RIF Ultra is a nucleic acid amplification test for *Mtb* with a lower limit of detection compared to the previous Xpert MTB/RIF generation, and, therefore, conferring higher sensitivity in paucibacillary specimens. This, however, comes at the expense of specificity, particularly in high TB incidence settings, resulting in 'false positives' [19]. WHO guidelines recommend Xpert MTB/RIF Ultra for TB diagnosis among adults and children acknowledging that further evaluation, particularly of the role of Xpert MTB/RIF Ultra for TB screening, is needed [20,21].

FLOW-TB is an advanced enzyme-linked immunosorbent assay for the detection of *Mtb* lipoarabinomannan (a mycobacterial cell wall component) in urinary specimens with results available within 65 minutes [22].

The T-cell activation marker-TB assay (TAM-TB) detects Mtb-specific CD4 T-cells through *in-vitro* antigen stimulation with *Mtb*-derived peptides, i.e., from ESAT-6 and CFP-10, followed by flow cytometry. TAM-TB discriminated latent *Mtb* infection from TB in freshly collected blood with 83% sensitivity and 96-98% specificity in previous studies. Further, TAM-TB may detect early TB disease progression up to 9 months prior to the identification of *Mtb* in sputum [23–25].

Multiple transcriptomic signatures, capturing the host response to TB, have been described as promising candidate tests for earlier TB diagnosis (up to two years before microbiological diagnosis). An individual patient data meta-analysis suggested equivalent performance of eight signatures, with 25-40% sensitivity and 92-95% specificity 0-24 months before TB diagnosis. Diagnostic accuracy of each signature improved as the interval between testing and microbiological TB diagnosis shortened [26]. Several signatures have been developed into polymerase chain reaction (PCR)-based assays to facilitate real-time implementation: the recent CORTIS trial reported sensitivity of 48% and specificity of 75% for incipient TB for the RISK-11 signature [27]. Cepheid have developed a 3-transcript TB score into a fully automated

For peer review only

in-cartridge PCR assay performed on finger-prick blood using the Xpert platform (Xpert TB Host Response RUO Prototype cartridge). This cartridge will be evaluated using freshly collected specimens in ERASE-TB; storage of RNA-stabilised blood samples also allows for retrospective evaluation of additional transcriptomic signatures in our cohort [28,29]

An alternative approach to capture the host response to TB is through protein-based biomarker signatures. Candidate tests in this category include a serum- or plasma-based multiplex assay assessing 13 protein biomarkers (CRP, procalcitonin [30], sTREM-1 [31,32], angiopoietin-2 [33,34], interleukin-6 [35], TRAIL [36] and IP-10 [37]) that is being developed by the London School of Hygiene and Tropical Medicine; in additon, a seven biomarker signauture is under development as a point-of-care test for TB diagnosis, with 94% sensitivity and 73% specificity detected in previous work [38].

Statistical analyses

Baseline characteristics and analytical data will be summarized using descriptive statistics inclusive of mean, median, range, standard deviation, and absolute as well as relative frequencies depending on the nature of data. A logistic regression model will be used to identify characteristics of TB among ICs, households and HHCs that are predictive of incident TB. From the study database, we will simulate algorithms of different tests to obtain the testing combination with the best accuracy. We will couple tests with a mathematical model that quantifies the risk of infection and/or disease to enhance predictive performance. The reporting of the development of the prediction model will follow the Transparent Reporting of a multivariable prediction model for Individual Prognosis Or Diagnosis (TRIPOD) Initiative [39].

The validation of novel diagnostic tests for detecting TB will be analysed as a 1:4 matched nested case-control study with HHCs diagnosed with TB at baseline and during follow-up serving as cases, and HHCs who do not develop TB during follow-up as controls; controls will be matched for site, age, sex, HIV status and other risk factors for developing TB. Sensitivity

For peer review only

and specificity of novel tests will be determined using pre-existing positive/negative cut-offs where these exist [40]; and receiver operating curves (ROC) constructed with area under the ROC curve calculated. For tests aiming to identify individuals at high risk of TB in the future, only HHCs who are diagnosed with TB during follow-up will serve as cases (i.e. those diagnosed with TB at baseline will be excluded). Stored samples from all timepoints will be retrieved and diagnostic accuracy (i.e. sensitivity and specificity) of the novel test determined at different time-points before TB diagnosis. The decision of assigning the “active TB” endpoints to participants will be blinded from the new test results to avoid inclusion bias.

Data management

All source data will be kept confidential in secured locations with restricted access by authorized personnel only inclusive of monitors, auditors and reviewers of ethical and regulatory committees in line with applicable data privacy regulations. Each participant is asked to consent to this handling of the data, and is assigned a pseudonymous identification number that is used throughout the study on all source data.

Accurate documentation of paper-based and electronic source data, e.g., original records and certified copies of original records, progress notes, screening logs, and recorded data from automated instruments, will be maintained. The pseudonymized clinical data captured on paper-based Case Report Forms will be entered at the sites into a database using the web-based Clinical Data Management System of OpenClinica (OpenClinica LLC, Waltham, MA, USA). The study specific database has been built, maintained and hosted by the LMU Klinikum on a centralized secure server. Data modifications and necessary corrections performed in the database also within the context of double data entry will be documented and tracked in audit trails. Data quality and plausibility are assured by a series of pre-programmed edit and range checks in OpenClinica. Further validation checks are programmed in Stata (Statacorp, College Station, TX, USA) with extracts of the database and electronically received data, e.g., spirometry, dCXR and laboratory, will be integrated into analyses of datasets.

For peer review only

Monitoring

Assigned study monitors will visit the sites at regular intervals physically and/or virtually in addition to frequent day-to-day communication. Close follow-up on all study-related aspects will be performed to ascertain compliance with standards of Good Clinical Practice, the Declaration of Helsinki, and other local and national regulatory guidelines inclusive of guidelines for infection prevention and control of airborne-transmitted diseases, e.g., social distancing in well-ventilated spaces, and wearing of personal protective equipment. In particular, monitors that support designated study personnel are responsible to verify (I) adequacy of the study personnels' qualifications and facilities, (II) accuracy of informed consent procedures and patient eligibility, (III) adherence to the study protocol, (IV) protection of rights and well-being of participants, (V) adherence to infection prevention and control measures (VI) accuracy and completeness of study documents and other study-related records, and (VII) maintenance of source documents.

Ethics and dissemination

The study protocol and informed consent/assent documents have been approved by regulatory and ethical committees of the participating institutions [Medical Research Council in Zimbabwe (MRCZ/A/2618), the National Health Research Ethics Committee in Tanzania (TMDA-WEB0021/CTR/0004/03), the National Bioethics Committee for Health in Mozambique (541/CNBS/21), and the ethical committees of London School of Hygiene & Tropical Medicine, United Kingdom (22522-2), and the medical faculty of the Ludwig-Maximilians-Universität München, Germany (20-0771)].

Adult ICs and HHCs are asked for written informed consent prior to their participation. Underage HHCs are asked for assent in addition to obtaining the consent of their legal guardians/parents; with ages for assent depending on local guidance. In case of illiteracy, the participant is asked to give its consent by fingerprint while an adult impartial, literate witness

For peer review only

present during the entire consent procedure signs the consent on behalf. All participants have the right to withdraw from the study at any time. Findings derived from ERASE-TB will be presented at scientific conferences, and published in peer-reviewed international journals.

Current study status

The recruitment of ICs and HHCs is in progress in Zimbabwe, Mozambique and Tanzania since March, August and September 2021, respectively. The follow-up of HHCs is anticipated to be completed in March, August and September 2023 in Zimbabwe, Mozambique and Tanzania, respectively; laboratory analyses are estimated to be performed by December 2024.

Author contributions

The study proposal and protocol were written by NH, KK with scientific input from CK, TM, CG, JM. ETM, UP, DB, AM wrote the initial manuscript with scientific input on the database and data management section from FR, TA. NH, KK critically reviewed the initial draft of the manuscript. KH, GK, CJC, ENN, TA, provided critical feedback on the manuscript. All authors have read and approved the final version of the manuscript.

Funding statement

ERASE-TB is part of the EDCTP2 programme supported by the European Union (grant number RIA2018D-2508-ERASE-TB), the German Center for Infection Research (DZIF) grant number: 02.710 and the Swedish Research Council (220-23602). CJC is funded by the Wellcome Trust (203905/Z/16/Z). Cepheid, Inc., and SD Biosensor provided test kits and analyzers at no cost to the Consortium.

Acknowledgments

We are grateful to the study personnel from the Biomedical Research and Training Institute and the Zvitambo Research Institute, Zimbabwe, the Instituto Nacional de Saúde, Mozambique, and the National Institute for Medical Research - Mbeya Medical Research

For peer review only

Centre, Tanzania for their exceptional efforts and contributions, which made this research possible.

Consortium authorship

The following are members of the ERASE-TB consortium: Anna Shepherd[a], Hazel M Dockrell[a], Judith Bruchfeld[b], Christopher Sundling[b], Charles Sandy[c], Mishelle Mugava[c], Tsitsi Bandason[c], Martha Chipinduro[c], Kuda Mutasa[d], Sandra Rukobo[d], Lwitiho Sudi[e], Antelmo Haule[e], Emmanuel Sichone[e], Paschal Qwaray[e], Bariki Mtafya[e], Harrieth Mwambola[e], Lilian Minja[e], Issa Sabi[e], Peter Edwin[e], Dogo Ngalison[e], Stella Luswema[e], Willyhelmina Olomi[e], Doreen Pamba[e], Simeon Mwanyonga[e], Celina Nhamuave[f], António Machiana[f], Carla Madeira[f], Emelva Manhiça[f], Nádia Sitoe[f], Jorge Ribeiro[f], Christof Geldmacher[g], Andrea Rachow[g], Olena Ivanova[g], Laura Olbrich[g], Elmar Saathoff[g], Michael Hoelscher[g].

[a]Faculty of Infectious and Tropical Diseases, Department of Infection Biology, London School of Hygiene & Tropical Medicine, London, UK

[b]Karolinska Institute, Stockholm, Sweden

[c]Biomedical Research and Training Institute, Harare, Zimbabwe

[d]Zvitambo Research Institute, Harare, Zimbabwe

[e]National Institute for Medical Research - Mbeya Medical Research Centre, Mbeya, Tanzania

[f]Instituto Nacional de Saúde, Marracuene, Mozambique

[g]Division of Infectious Diseases and Tropical Medicine, University Hospital, LMU Munich, Munich, Germany

Competing interests statement

None declaired.

Disclaimer

Not applicable.

For peer review only

References

1 Global tuberculosis report 2021. https://www.who.int/publications/i/item/9789240037021 (accessed 28 Oct 2021).
2 The end TB STraTegy global strategy and targets for tuberculosis prevention, care and control after 2015 a. Published Online First: 2014.https://www.who.int/tb/strategy/End_TB_Strategy.pdf (accessed 28 Oct 2021).
3 WHO. Global Tuberculosis Report. 2020.https://www.who.int/publications/i/item/9789240013131 (accessed 31 Jul 2021).
4 Snider B, Patel B, McBean E. Asymptomatic Cases, the Hidden Challenge in Predicting COVID-19 Caseload Increases. *Infect Dis Reports 2021, Vol 13, Pages 340-347* 2021;**13**:340–7. doi:10.3390/IDR13020033
5 TB Disease - Symptoms, treatment & prevention - TBFacts. https://tbfacts.org/tb-disease/ (accessed 28 Aug 2021).
6 Honeyborne I, McHugh TD, Phillips PPJ, *et al.* Molecular Bacterial Load Assay, a Culture-Free Biomarker for Rapid and Accurate Quantification of Sputum Mycobacterium tuberculosis Bacillary Load during Treatment. *J Clin Microbiol* 2011;**49**:3905. doi:10.1128/JCM.00547-11
7 UNIAIDS. UNAIDS Global AIDS Update. 2021. https://www.unaids.org/sites/default/files/media_asset/2021-global-aids-update_en.pdf (accessed 28 Oct 2021).
8 ZIMBABWE POPULATION CENSUS 2012 WOMEN AND MEN PROFILE SUMMARY REPORT. Published Online First: 2016.https://www.zimstat.co.zw/wp-content/uploads/publications/Social/Gender/Women-and-Men-Report-2012.pdf (accessed 15 Oct 2021).
9 Tanzania Census 2012 - Dashboard. http://dataforall.org/dashboard/tanzania/ (accessed 15 Oct 2021).
10 ZIMBABWE POPULATION-BASED HIV IMPACT ASSESSMENT ZIMPHIA 2020 KEY FINDINGS. https://phia.icap.columbia.edu/wp-content/uploads/2020/11/ZIMPHIA-2020-Summary-Sheet_Web.pdf (accessed 3 Sep 2021).
11 Xadreque H, Maunze A, Dade M, *et al.* IV RECENSEAMENTO GERAL DA POPULAÇÃO E HABITAÇÃO. 2017.http://www.ine.gov.mz/iv-rgph-2017/mocambique/censo-2017-brochura-dos-resultados-definitivos-do-iv-rgph-nacional.pdf (accessed 31 Jul 2021).
12 Population of Cities in Zimbabwe (2021). https://worldpopulationreview.com/countries/cities/zimbabwe (accessed 3 Aug 2021).
13 World atlas. https://knoema.com/atlas/United-Republic-of-Tanzania. (accessed 26 Jul 2021).
14 Prevalence of HIV, total (% of population ages 15-49) | Data. https://data.worldbank.org/indicator/SH.DYN.AIDS.ZS?locations=ZW (accessed 3 Aug 2021).
15 Incidence of tuberculosis (per 100,000 people) - Zimbabwe | Data. https://data.worldbank.org/indicator/SH.TBS.INCD?locations (accessed 3 Aug 2021).
16 Fox GJ, Nhung N V., Sy DN, *et al.* Household-Contact Investigation for Detection of Tuberculosis in Vietnam. *N Engl J Med* 2018;**378**:221–9. doi:10.1056/NEJMOA1700209
17 Qin ZZ, Ahmed S, Sarker MS, *et al.* Tuberculosis detection from chest x-rays for triaging in a high tuberculosis-burden setting: an evaluation of five artificial intelligence algorithms. *Lancet Digit Heal* 2021;**3**:e543–54. doi:10.1016/S2589-7500(21)00116-3
18 MacPherson P, Webb EL, Kamchedzera W, *et al.* Computer-aided X-ray screening for tuberculosis and HIV testing among adults with cough in Malawi (the PROSPECT study): A randomised trial and cost-effectiveness analysis. *PLoS Med* 2021;**18**:1–17. doi:10.1371/journal.pmed.1003752
19 Dorman S, Schumacher S, Alland D, *et al.* Xpert MTB/RIF Ultra for detection of Mycobacterium tuberculosis and rifampicin resistance: a prospective multicentre diagnostic accuracy study. *Lancet Infect Dis* 2018;**18**:76–84. doi:10.1016/S1473-

For peer review only

3099(17)30691-6

20 WHO Meeting Report of a Technical Expert Consultation: Non-inferiority analysis of Xpert MTB/RIF Ultra compared to Xpert MTB/RIF. Published Online First: 2017.http://apps.who.int/bookorders. (accessed 19 Oct 2021).

21 World Health Organization. WHO consolidated guidelines on tuberculosis: Module 2: Screening. 2021.https://www.who.int/publications/i/item/9789240022676 (accessed 9 Dec 2021).

22 Hamasur B, Bruchfeld J, van Helden P, *et al.* A sensitive urinary lipoarabinomannan test for tuberculosis. *PLoS One* 2015;**10**. doi:10.1371/JOURNAL.PONE.0123457

23 Portevin D, Moukambi F, Clowes P, *et al.* Assessment of the novel T-cell activation marker-tuberculosis assay for diagnosis of active tuberculosis in children: a prospective proof-of-concept study. *Lancet Infect Dis* 2014;**14**:931–8. doi:10.1016/S1473-3099(14)70884-9

24 Schuetz A, Haule A, Reither K, *et al.* Monitoring CD27 expression to evaluate Mycobacterium tuberculosis activity in HIV-1 infected individuals in vivo. *PLoS One* 2011;**6**. doi:10.1371/JOURNAL.PONE.0027284

25 Ahmed MIM, Ntinginya NE, Kibiki G, *et al.* Phenotypic Changes on Mycobacterium Tuberculosis-Specific CD4 T Cells as Surrogate Markers for Tuberculosis Treatment Efficacy. *Front Immunol* 2018;**9**:2247. doi:10.3389/FIMMU.2018.02247/BIBTEX

26 Gupta RK, Turner CT, Venturini C, *et al.* Concise whole blood transcriptional signatures for incipient tuberculosis: a systematic review and patient-level pooled meta-analysis. *Lancet Respir Med* 2020;**8**:395–406. doi:http://dx.doi.org/10.1016/S2213-2600%2819%2930282-6

27 Scriba TJ, Fiore-Gartland A, Penn-Nicholson A, *et al.* Biomarker-guided tuberculosis preventive therapy (CORTIS): a randomised controlled trial. *Lancet Infect Dis* 2021;**21**:354–65. doi:10.1016/S1473-3099(20)30914-2/ATTACHMENT/EB270186-669A-4F82-8A80-FB38FA1FB796/MMC1.PDF

28 Warsinske H, Rao A, Moreira F, *et al.* Assessment of Validity of a Blood-Based 3-Gene Signature Score for Progression and Diagnosis of Tuberculosis, Disease Severity, and Treatment Response. *JAMA Netw open* 2018;**1**:e183779. doi:10.1001/JAMANETWORKOPEN.2018.3779

29 Sweeney TE, Braviak L, Tato CM, *et al.* Genome-wide expression for diagnosis of pulmonary tuberculosis: a multicohort analysis. *Lancet Respir Med* 2016;**4**:213–24. doi:10.1016/S2213-2600(16)00048-5

30 Mendelson F, Griesel R, Tiffin N, *et al.* C-reactive protein and procalcitonin to discriminate between tuberculosis, Pneumocystis jirovecii pneumonia, and bacterial pneumonia in HIV-infected inpatients meeting WHO criteria for seriously ill: a prospective cohort study. *BMC Infect Dis* 2018;**18**. doi:10.1186/S12879-018-3303-6

31 Huang C, Lee L, Ho C, *et al.* High serum levels of procalcitonin and soluble TREM-1 correlated with poor prognosis in pulmonary tuberculosis. *J Infect* 2014;**68**:440–7. doi:10.1016/J.JINF.2013.12.012

32 Feng J, Su W, Pan S, *et al.* Role of TREM-1 in pulmonary tuberculosis patients-analysis of serum soluble TREM-1 levels. *Sci Rep* 2018;**8**. doi:10.1038/S41598-018-26478-2

33 Kumar NP, Velayutham B, Nair D, *et al.* Angiopoietins as biomarkers of disease severity and bacterial burden in pulmonary tuberculosis. *Int J Tuberc Lung Dis* 2017;**21**:93. doi:10.5588/IJTLD.16.0565

34 Kathamuthu G, Moideen K, Baskaran D, *et al.* Tuberculous lymphadenitis is associated with altered levels of circulating angiogenic factors. *Int J Tuberc Lung Dis* 2018;**22**:557–66. doi:10.5588/IJTLD.17.0609

35 Sivro A, McKinnon L, Yende-Zuma N, *et al.* Plasma Cytokine Predictors of Tuberculosis Recurrence in Antiretroviral-Treated Human Immunodeficiency Virus-infected Individuals from Durban, South Africa. *Clin Infect Dis* 2017;**65**:819–26. doi:10.1093/CID/CIX357

36 La Manna M, Orlando V, Li Donni P, *et al.* Identification of plasma biomarkers for discrimination between tuberculosis infection/disease and pulmonary non tuberculosis

For peer review only

disease. *PLoS One* 2018;**13**. doi:10.1371/JOURNAL.PONE.0192664

37 Sivro A, McKinnon L, Yende-Zuma N, *et al.* Plasma Cytokine Predictors of Tuberculosis Recurrence in Antiretroviral-Treated Human Immunodeficiency Virus-infected Individuals from Durban, South Africa. *Clin Infect Dis* 2017;**65**:819–26. doi:10.1093/CID/CIX357

38 Chegou NN, Sutherland JS, Malherbe S, *et al.* Diagnostic performance of a seven-marker serum protein biosignature for the diagnosis of active TB disease in African primary healthcare clinic attendees with signs and symptoms suggestive of TB. *Thorax* 2016;**71**:785–94. doi:10.1136/thoraxjnl-2015-207999

39 Collins GS, Reitsma JB, Altman DG, *et al.* Transparent reporting of a multivariable prediction model for individual prognosis or diagnosis (TRIPOD): The TRIPOD Statement. *BMC Med* 2015;**13**:1–10. doi:10.1186/S12916-014-0241-Z/TABLES/1

40 Trevethan R. Sensitivity, Specificity, and Predictive Values: Foundations, Pliabilities, and Pitfalls in Research and Practice. *Front public Heal* 2017;**5**. doi:10.3389/FPUBH.2017.00307

41 Agency ZS. QUALITY CONTROL MANUAL Provincial Report Harare Central Census Office. https://www.zimstat.co.zw/wp-content/uploads/publications/Population/population/Harare.pdf (accessed 3 Aug 2021).

42 Mozambique Population 2021 (Demographics, Maps, Graphs). https://worldpopulationreview.com/countries/mozambique-population (accessed 27 Jul 2021).

43 ZIMBABWE NATIONAL HIV/AIDS AND TUBERCULOSIS CONTROL PROGRAMMES. Republic of Zimbabwe NATIONAL HIV/AIDS AND TUBERCULOSIS CONTROL PROGRAMMES. https://www.who.int/hiv/pub/guidelines/zimbabwe.pdf (accessed 1 Sep 2021).

For peer review only

Figure legend

Figure 1. The ERASE-TB consortium

Abbreviations: ERASE-TB=Early Risk Assessment in TB contactS by new diagnostic tEsts

Figure 2. Location and characteristics of ERASE-TB study sites

Notes: The location of each study site is indicated by a red asterix. Source data used within this figure are taken from the references [7,8,41–43,9–15].

Abbreviations: ERASE=Early Risk Assessment in TB contactS by new diagnostic tEsts; TB=tuberculosis; HIV=human immunodeficiency virus

Figure 3. Eligibility criteria and schedules of events for index cases and household contacts

Notes: **A**=depending on the time point of study enrollment and consequently on the duration available for follow-up, i.e. 18 or 24 months, the follow-up visit at 24 months ±30 days may be conditional; **B**=the follow-up visit by phone may be conducted after the last scheduled follow-up visit at 18 months ±30 days or 24 months ±30 days to assess whether symptoms suggestive of TB have occurred, TB diagnosis has been made or anti-TB treatment has been initiated; **C**=unwell visits by phone or on-site may be conducted between scheduled follow-up visits if a participant presents at a recruitment healthcare facility with signs and symptoms suggestive of TB; **D**=coached spontaneous or induced sputum collection for storage at scheduled follow-up visit at 18 months ±30 days or 24 months ±30 days, and for repetition of HIV testing if tested negative at baseline; **E**=coached spontaneous or induced sputum collection upon the decision of the investigating team for testing by Xpert MTB/RIF Ultra if participant presents with signs and symptoms suggestive of TB; **F**=coached spontaneous or induced sputum collection in case of Xpert MTB/RIF Ultra positivity or strong clinical suspicion of TB for repetition of the Xpert MTB/RIF Ultra; **G**=in case of HIV positivity to be followed by the assessment of CD4 counts; **H**=CXR to be conducted at an unscheduled on-site unwell visit upon the decision of the investigating team depending on the nature of symptoms reported, and the time elapsed since the last CXR including its findings; **I**=not to be conducted among pregnant women;

For peer review only

610 **J**=stored venous blood includes 6mL EDTA blood for whole blood and plasma, 4mL serum and 2.5mL PAXgene blood, all samples will be deep frozen for retrospective testing using new diagnostics as described in text; **K**=in case the evaluation of symptoms of a participant unable to present at a recruitment healthcare facility is required an unscheduled on-site or home visit will be arranged by phone, the resolution of symptoms can alternatively be addressed by phone; **L**=collection of PBMC at baseline and follow-up visit at 6 months ±30 days is optional, thus will not be performed at each participating site and for each participant; **M**=in case the evaluation of symptoms of a participant unable to present at a recruitment healthcare facility is required or doubtful if required an unscheduled unwell visit by phone will be arranged, the resolution of symptoms can alternatively be addressed by phone; **N**=spirometry and/or diabetes (HbA1c) will be performed at scheduled follow-up visits at 6 months ±30 days, 12 months ±30 days and 18 or 24 months ±30 days if required or not performed at baseline, anaemia (Hb) will be performed at baseline and scheduled follow-up visits at 6 months ±30 days, 12 months ±30 days and 18 or 24 months ±30 days if possible; **O**=blood pressure measurement will be performed at baseline and scheduled follow-up visits at 6 months ±30 days, 12 months ±30 days and 18 or 24 months ±30 days; **P**=WGS to be performed once *Mtb* infection is confirmed and an isolate could be recovered.

Abbreviations: TB=tuberculosis; WHO=World Health Organization; HIV=human immunodeficiency virus; WGS=whole genome sequencing; MBLA=molecular bacterial load assay; CXR=chest radiograph; IGRA=interferon gamma release assay; PBMC=peripheral blood mononuclear cell; TAM-TB=T- cell activation marker tuberculosis; MTB=*Mycobacterium tuberculosis*; RIF=rifampicin, Hb=haemoglobin; HbA1c=glycated hemoglobin; LAM=lioparabinomannan; CD4=cluster of differentiation 4

Figure 4. Study design

Abbreviations: HHC=household contact; TB=tuberculosis; IC=index case; WHO=World Health Organization; CXR= chest radiograph; SS=symptom score; MTB=*Mycobacterium tuberculosis*; RIF=rifampicin; pos=positive; CT=cpmputer tomography; FU=follow-up

For peer review only

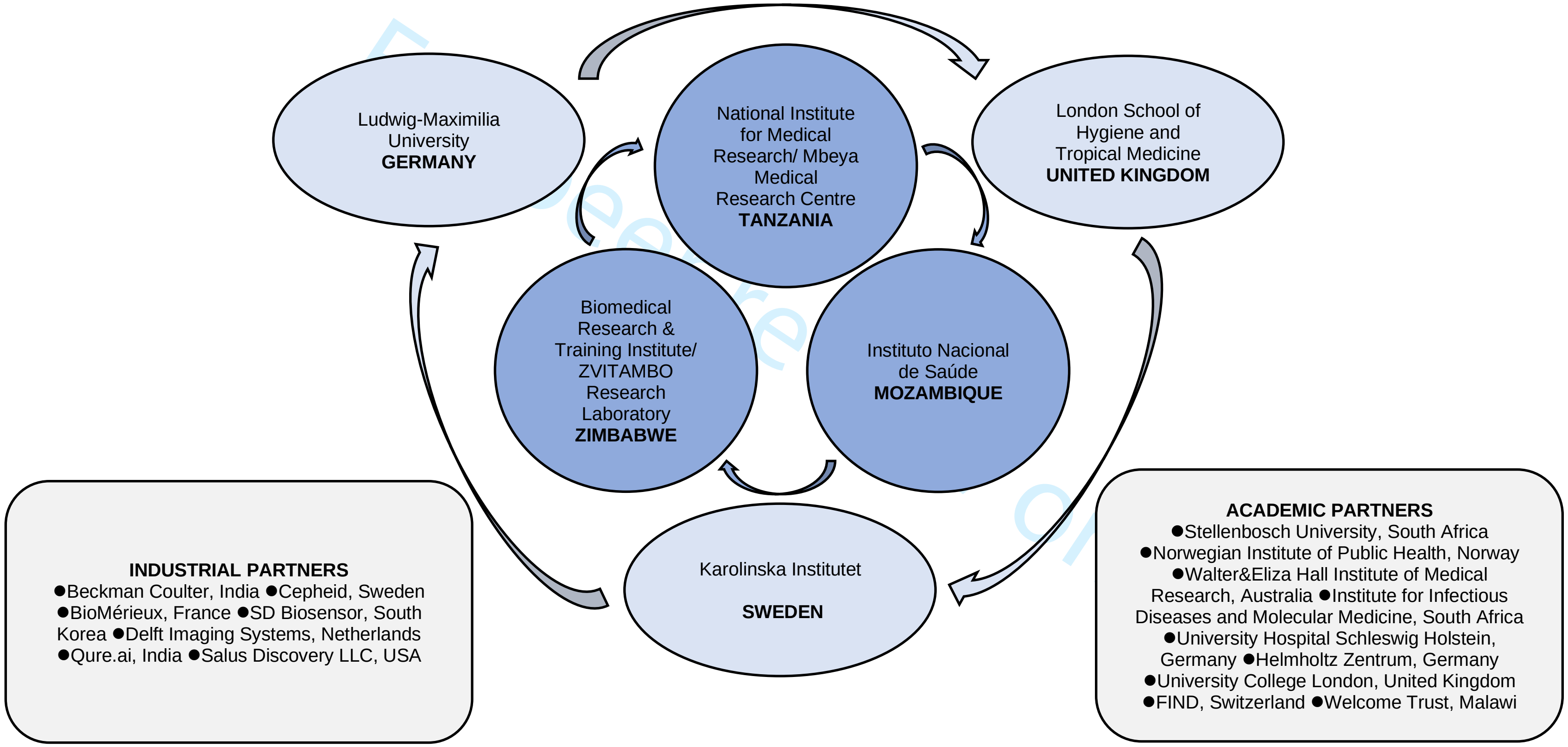

Figure 1. The ERASE-TB consortium

For peer review only - http://bmjopen.bmj.com/site/about/guidelines.xhtml

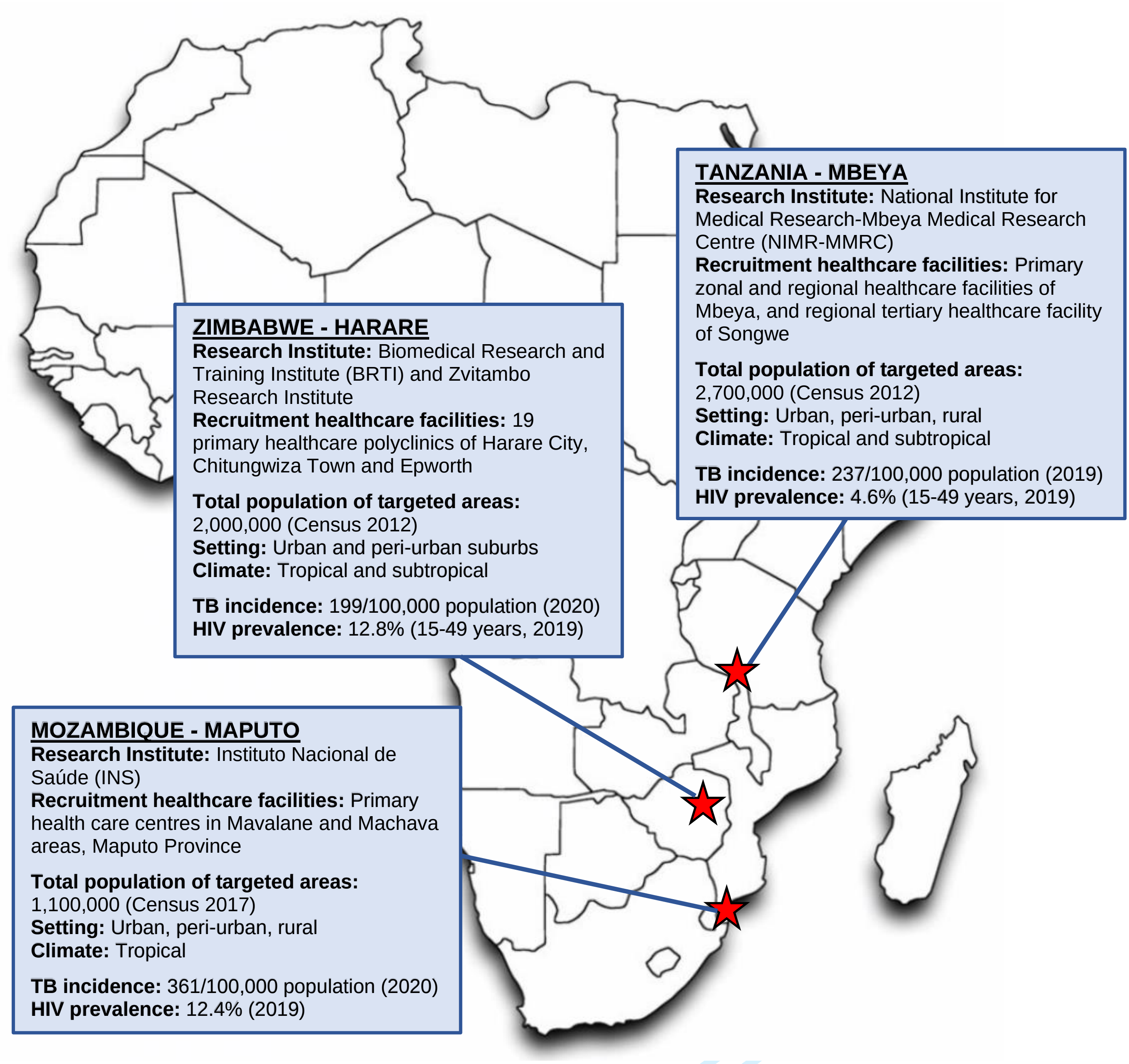

Figure 2. Location and characteristics of ERASE-TB study sites

INDEX CASE

INCLUSION CRITERIA
- Aged ≥18 years
- Living with at least another person aged ≥10 years in the same household with a firm unchanged address for 6 months
- Pulmonary TB diagnosis within 4 weeks prior to enrolment
- Spontaneous sputum production
- Positive sputum smear, i.e. >+2 positivity by Ziehl-Neelsen stain or Auramine-O stain OR ≥+1 positivity on WHO symptom scale OR ≥medium positivity by GeneXpert (if no microscopy performed)
- <7 daily doses of anti-TB treatment since diagnosis
- Voluntary written informed consent obtained

IN

EVENTS AT BASELINE/SCREENING VISIT
- Administration of socio-demographic questionnaire
- Assessment of vital signs, medical history
- Digital CXR (if available)[I]

Specimen investigations
- Sputum: culture, storage, WGS, MBLA

NO FOLLOW-UP

HOUSEHOLD CONTACT

EXCLUSION CRITERIA
- Treatment for TB within the past 30 days OR on current TB treatment OR on current preventive TB therapy (if HIV negative) at the tim of enrolment

OUT

INCLUSION CRITERIA
- Aged ≥10 years
- Substantial exposure, i.e. spending ≥3 nights/week over the past 4 weeks in the same household as the index case,
- Voluntary written informed consent or parent/guardian consent & assent in case of minors obtained

IN

EVENTS AT BASELINE/ ±14days of SCREENING VISIT
- Administration of socio-demographic questionnaire
- Assessment vital signs, medical history
- Screening for diabetes (HbA1c)[N], high blood pressure[O], anaemia (Hb)[N], WHO TB symptoms
- Digital CXR[I], spirometry[N]

Specimen investigations
- Sputum: Xpert MTB/RIF Ultra[E], culture[F], storage, WGS[P]
- Blood: HIV/CD4 counts[G], Cepheid host response, TAM-TB, IGRA SD Biosensor, PBMC[L], storage[J]
- Urine: dipstick/LAM, storage

FOLLOW-UP

EVENTS AT UNSCHEDULED UNWELL VISIT(S) (phone/on-site)[C,K, M]
- Assessment of vital signs, medical history
- Screening for high blood pressure, WHO TB symptoms,
- Digital CRX[H,I]

Specimen investigations
- Sputum: Xpert MTB/RIF Ultra, culture, storage, WGS[P]
- Blood: Cepheid host response, TAM-TB, storage[J]
- Urine: dipstick/LAM, storage

EVENTS AT UN-SCHEDULED FOLLOW-UP VISIT(S)[B] (phone)
- Assessment of medical history
- Screening for WHO TB symptoms

Specimen investigations
- None

EVENTS AT 6months ±30days/ 12months ±30days/ 18months ±30days/ 24months ±30days[A] FOLLOW-UP VISITS
- Assessment of vital signs, medical history
- Screening for high blood pressure, WHO TB symptoms
- Digital CXR[I], spirometry[N]

Specimen investigations
- Sputum: Xpert MTB/RIF Ultra[E], culture[F], storage[D], WGS[P]
- Blood: Cepheid host response, TAM-TB, PBMC[L], storage
- Urine: dipstick/LAM, storage[J]

OUT TB+

Optional

Optional

For peer review only - http://bmjopen.bmj.com/site/about/guidelines.xhtml

Figure 3. Eligibility criteria and schedules of events for index cases and household contacts

For peer review only

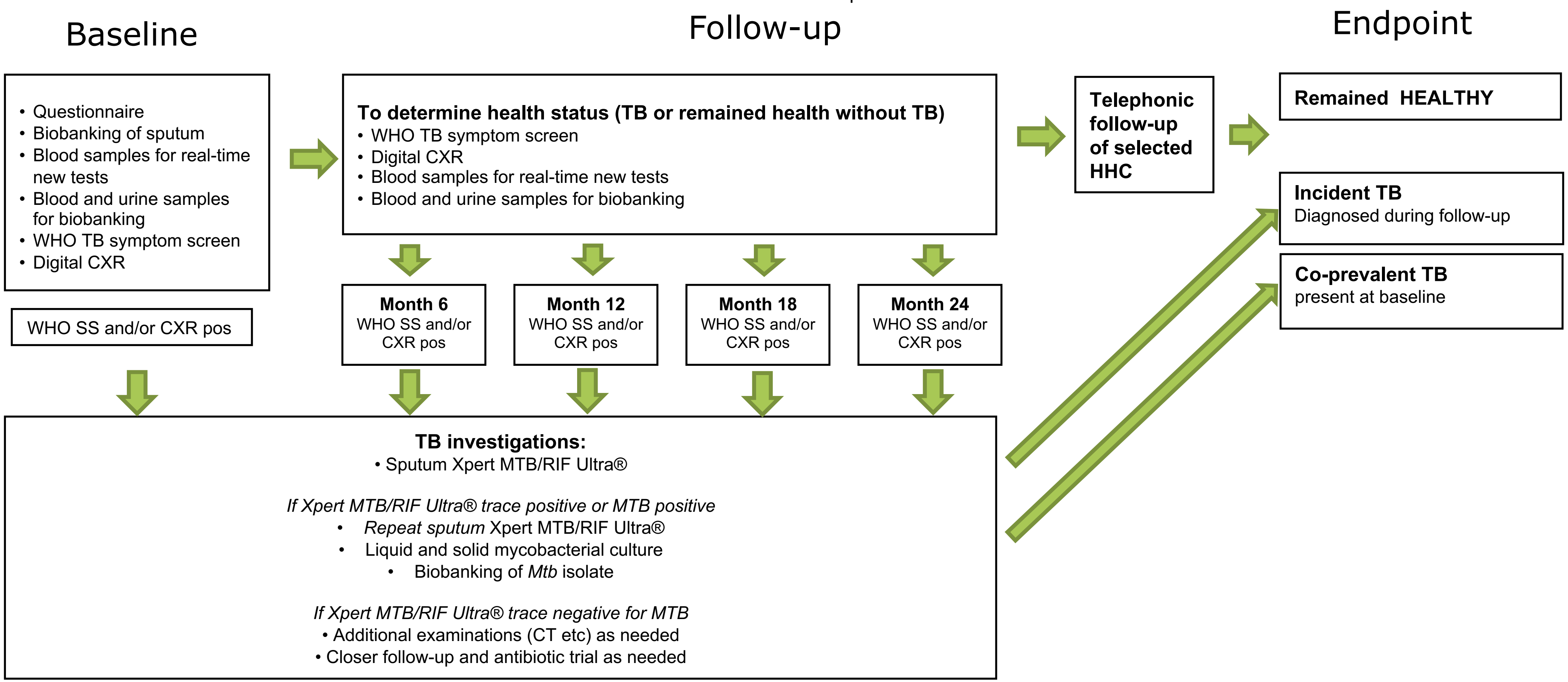

Figure 4. Study design.

Section & Topic	No	Item	Reported on page #
**TITLE OR ABSTRACT**			
	**1**	Identification as a study of diagnostic accuracy using at least one measure of accuracy (such as sensitivity, specificity, predictive values, or AUC)	2
**ABSTRACT**			
	**2**	Structured summary of study design, methods, results, and conclusions (for specific guidance, see STARD for Abstracts)	2-3
**INTRODUCTION**			
	**3**	Scientific and clinical background, including the intended use and clinical role of the index test	5-6
	**4**	Study objectives and hypotheses	6
**METHODS**			
Study design	**5**	Whether data collection was planned before the index test and reference standard were performed (prospective study) or after (retrospective study)	6-7
Participants	**6**	Eligibility criteria	7 (Figure 3)
	**7**	On what basis potentially eligible participants were identified (such as symptoms, results from previous tests, inclusion in registry)	7 (Figure 3)
	**8**	Where and when potentially eligible participants were identified (setting, location and dates)	7
	**9**	Whether participants formed a consecutive, random or convenience series	7, 11
Test methods	**10a**	Index test, in sufficient detail to allow replication	8-10, 11-13
	**10b**	Reference standard, in sufficient detail to allow replication	8-10, 11-13
	**11**	Rationale for choosing the reference standard (if alternatives exist)	8-10, 11-13
	**12a**	Definition of and rationale for test positivity cut-offs or result categories of the index test, distinguishing pre-specified from exploratory	13-14
	**12b**	Definition of and rationale for test positivity cut-offs or result categories of the reference standard, distinguishing pre-specified from exploratory	13-14
	**13a**	Whether clinical information and reference standard results were available to the performers/readers of the index test	13-14
	**13b**	Whether clinical information and index test results were available to the assessors of the reference standard	13-14
Analysis	**14**	Methods for estimating or comparing measures of diagnostic accuracy	13-14
	**15**	How indeterminate index test or reference standard results were handled	13-14
	**16**	How missing data on the index test and reference standard were handled	14
	**17**	Any analyses of variability in diagnostic accuracy, distinguishing pre-specified from exploratory	13-14
	**18**	Intended sample size and how it was determined	11
**RESULTS**			
Participants	**19**	Flow of participants, using a diagram	8-10 (Figure 3)
	**20**	Baseline demographic and clinical characteristics of participants	8-10 (Figure 3)
	**21a**	Distribution of severity of disease in those with the target condition	
	**21b**	Distribution of alternative diagnoses in those without the target condition	
	**22**	Time interval and any clinical interventions between index test and reference standard	
Test results	**23**	Cross-tabulation of the index test results (or their distribution) by the results of the reference standard	
	**24**	Estimates of diagnostic accuracy and their precision (such as 95% confidence intervals)	13-14
	**25**	Any adverse events from performing the index test or the reference standard	
**DISCUSSION**			
	**26**	Study limitations, including sources of potential bias, statistical uncertainty, and generalisability	3
	**27**	Implications for practice, including the intended use and clinical role of the index test	5-6
**OTHER INFORMATION**			
	**28**	Registration number and name of registry	2
	**29**	Where the full study protocol can be accessed	
	**30**	Sources of funding and other support; role of funders	16

For peer review only - http://bmjopen.bmj.com/site/about/guidelines.xhtml

cc BY SA

STARD 2015

AIM

STARD stands for “Standards for Reporting Diagnostic accuracy studies”. This list of items was developed to contribute to the completeness and transparency of reporting of diagnostic accuracy studies. Authors can use the list to write informative study reports. Editors and peer-reviewers can use it to evaluate whether the information has been included in manuscripts submitted for publication.

EXPLANATION

A **diagnostic accuracy study** evaluates the ability of one or more medical tests to correctly classify study participants as having a **target condition.** This can be a disease, a disease stage, response or benefit from therapy, or an event or condition in the future. A medical test can be an imaging procedure, a laboratory test, elements from history and physical examination, a combination of these, or any other method for collecting information about the current health status of a patient.

The test whose accuracy is evaluated is called **index test.** A study can evaluate the accuracy of one or more index tests. Evaluating the ability of a medical test to correctly classify patients is typically done by comparing the distribution of the index test results with those of the **reference standard**. The reference standard is the best available method for establishing the presence or absence of the target condition. An accuracy study can rely on one or more reference standards.

If test results are categorized as either positive or negative, the cross-tabulation of the index test results against those of the reference standard can be used to estimate the **sensitivity** of the index test (the proportion of participants *with* the target condition who have a positive index test), and its **specificity** (the proportion *without* the target condition who have a negative index test). From this cross-tabulation (sometimes referred to as the contingency or “2x2” table), several other accuracy statistics can be estimated, such as the positive and negative **predictive values** of the test. Confidence intervals around estimates of accuracy can then be calculated to quantify the statistical **precision** of the measurements.

If the index test results can take more than two values, categorization of test results as positive or negative requires a **test positivity cut-off**. When multiple such cut-offs can be defined, authors can report a receiver operating characteristic (ROC) curve which graphically represents the combination of sensitivity and specificity for each possible test positivity cut-off. The **area under the ROC curve** informs in a single numerical value about the overall diagnostic accuracy of the index test.

The **intended use** of a medical test can be diagnosis, screening, staging, monitoring, surveillance, prediction or prognosis. The **clinical role** of a test explains its position relative to existing tests in the clinical pathway. A replacement test, for example, replaces an existing test. A triage test is used before an existing test; an add-on test is used after an existing test.

Besides diagnostic accuracy, several other outcomes and statistics may be relevant in the evaluation of medical tests. Medical tests can also be used to classify patients for purposes other than diagnosis, such as staging or prognosis. The STARD list was not explicitly developed for these other outcomes, statistics, and study types, although most STARD items would still apply.

DEVELOPMENT

This STARD list was released in 2015. The 30 items were identified by an international expert group of methodologists, researchers, and editors. The guiding principle in the development of STARD was to select items that, when reported, would help readers to judge the potential for bias in the study, to appraise the applicability of the study findings and the validity of conclusions and recommendations. The list represents an update of the first version, which was published in 2003.

More information can be found on http://www.equator-network.org/reporting-guidelines/stard.

For peer review only
