# Supplementary material for: Early risk assessment in paediatric and adult household contacts of confirmed tuberculosis cases by novel diagnostic tests (ERASE-TB): protocol for a prospective, non-interventional, longitudinal, multicountry cohort study
Source: BMJ Open. 2022 Jul 19;12(7):e060985. doi: 10.1136/bmjopen-2022-060985 (PMC9301805; doi:10.1136/bmjopen-2022-060985)
Supplement: Reviewer comments [file bmjopen-2022-060985.reviewer_comments.pdf]

PEER REVIEW HISTORY

BMJ Open publishes all reviews undertaken for accepted manuscripts. Reviewers are asked to complete a checklist review form (**http://bmjopen.bmj.com/site/about/resources/checklist.pdf**) and are provided with free text boxes to elaborate on their assessment. These free text comments are reproduced below.

ARTICLE DETAILS

**TITLE (PROVISIONAL)**	Early risk assessment in pediatric and adult household contacts of confirmed tuberculosis cases by novel diagnostic tests (ERASE-TB): protocol for a prospective, non-interventional, longitudinal, multi-country cohort study
**AUTHORS**	Marambire, Edson; Banze, Denise; Mfinanga, Alfred; Mutsvangwa, Junior; Mbunda, Theodora; Ntinginya, Nyanda; Celso, Khosa; Kallenius, Gunilla; Calderwood, Claire J.; Geldmacher, Christof; Held, Kathrin; Appalarowthu, Tejaswi; Rieß, Friedrich; Panzner, Ursula; Heinrich, Norbert; Kranzer, Katharina

VERSION 1 – REVIEW

**REVIEWER**	Seas, Carlos<br>Universidad Peruana Cayetano Heredia
**REVIEW RETURNED**	19-Feb-2022

**GENERAL COMMENTS**	Congratulations for a very well designed study. I have few questions to you:<br>1. Given that the sample size will not allow you to make subgroup analysis, have you considered increasing it by recruiting more study sites or prolonging follow-up?<br>2. What is the gold standard for calculating sensitivity and specificity of the novel tests?<br>3. Can you elaborate more on the diagnostic utility of TAM-TB?. sensitivity seems suboptimal to use it as a predictor of TB progression<br>4. The tests that detect transcriptomic signatures lack sensitivity. Is the new Xpert test that you are going to use associated with higher sensitivity?<br>5. Are you going to test protein biomarker signatures in your study? if so, which one in particular?

**REVIEWER**	Davis, J. Lucian<br>Yale University, Epidemiology of Microbial Diseases<br><br>I have NIH grant funding for a trial to evaluate behavioral innovations for household TB contact investigation in Uganda, different from the protocol under review.<br>I declare that I have no other competing interests.
**REVIEW RETURNED**	24-Mar-2022

**GENERAL COMMENTS**	General Comments<br>Marambire et al present a study protocol for a prospective, observational, longitudinal cohort study of the performance of novel diagnostic and prognostic tests for incipient, preclinical, and active

	tuberculosis among household TB contacts in three countries in Southern Africa. The expected impact of the study will be to determine the accuracy of novel diagnostics and testing algorithms for early tuberculosis diagnosis and prediction of progression to active TB disease among household contacts.<br><br>Major Comments<br>The protocol is well-written and the study is very well-conceived overall. I do have a few important questions and comments outlined below:<br><br>Line 67 – The authors cite their plans to recruit highlight infectious cases as a strength, on grounds that it will maximize the number of TB diagnoses in the household cohort. However, recent systematic reviews (i.e., Velen et al ERJ 2021) suggest that the prevalence of TB among household contacts does not differ by index TB patient mycobacterial load. While this is a minor point, this limitation should be considered, and the strengths and limitations of the study design potentially revised.<br><br>Line 71 – The authors state that this study will provide more precise estimates of the sensitivity of novel diagnostic/prognostic tests compared to population-based cohorts with unknown timing of infection, and emphasize this as a strength of the study. I did not see information about how this timing will be determined in the study protocol; could the authors elaborate on how this will be done? How might it inform the analysis in practice, since the timing of infection is usually unknown in routine practice? One other concern is that, given the small number of co-prevalent or incident TB cases with genotypically confirmed household transmission, there may not be sufficient power for stratification of test results by timing of infection.<br><br>Line 115 – The authors state that this study will provide estimates of TB diagnostics for “early TB detection”, which they define as “before onward transmission occurs.” How can this be confirmed? It seems that this study design may not be able to exclude possible transmission to non-household contacts, since genotyping on these individuals will not be available. If so, the definition of “early TB detection” should be revised.<br><br>Line 251 – The study sample size is powered based on household contacts, with an assumed 3% diagnostic yield. While this is a reasonable estimate of the TB diagnostic yield using routine clinical or public health definitions, studies of novel diagnostic studies usually require more rigorously defined definitions of cases and controls, to avoid outcome misclassification and underestimates of sensitivity and specificity. With a rigorous gold standard, there may be high rates of indeterminate TB/non-TB outcomes due to various factors including culture contamination, false positive Xpert Ultra results (especially common in the household context), and difficulty adjudicating microbiologically-negative but clinically diagnosed cases of TB. In addition, if the goal is to provide reasonably precise estimates of the sensitivity of novel diagnostics the sample size of 64 TB cases will likely be inadequate, except in the case of extremely sensitive diagnostic assays. Will the authors be able to avoid an underpowered study if there are a lower-than-expected number of confirmed TB cases?<br><br>Lines 279-310, 323-326 – Patients will be enrolled over a two-year period, and some specimens stored for later testing. It should be

	specified which assays will be performed on stored specimens (information about the several assays that will be performed on fresh specimens has already been provided). For assays performed on bio-banked samples, could the authors state if the stability of the target analyte is known over the relevant storage period and conditions, and, if there are concerns about decay in signal over time, the authors might consider including additional TB-negative controls specifically matched on time in storage.<br><br>Lines 326-327 – A definition of the reference standard used to calculate sensitivity and specificity should be provided.<br><br>Line 333 – The authors mention plans for blinded interpretation of new test results which is a strength; however, other best practices for rigor and reporting of diagnostic studies (e.g. blinding to clinical information, choice and rationale for the reference standard etc.) that are outlined in the STARD reporting guidelines (BMJ. Z015;351:h5527) but not currently described in the study protocol. I suggest that these details be added and a completed STARD checklist submitted as supplementary material.

VERSION 1 – AUTHOR RESPONSE

Reviewer #1

Comment: Given that the sample size will not allow you to make subgroup analysis, have you considered increasing it by recruiting more study sites or prolonging follow-up?

Response: Thank you. We recognise that a larger sample size and longer follow up would result in more TB cases being detected, improving study power and facilitating sub-group analyses. However, the funding available for the study does not allow for this at the moment. We have applied for additional funding which, if successful, will increase the sample size and facilitate longer follow-up. This manuscript describes the study protocol, as is currently feasible.

Comment: What is the gold standard for calculating sensitivity and specificity of the novel tests?

Response: We prefer to use the term “reference standard” rather than gold standard as TB diagnosis is often challenging especially when trying to establish diagnosis early (at a time when bacterial load is relatively low). The reference standard will be TB diagnoses as categorised by an endline review committee, which will take into account the clinical presentation, chest X-ray findings, results of molecular tests and TB culture results. Multiple samples for molecular tests and culture will be obtained before a participant is referred for TB treatment.

Comment: Can you elaborate more on the diagnostic utility of TAM-TB?. sensitivity seems suboptimal to use it as a predictor of TB progression

Response: Thank you for this comment. The sensitivity of TAM-TB to predict future TB has not yet been established. In a study conducted among children with symptoms suggestive of TB, the sensitivity for TB diagnosis at time of presentation was 83% (https://pubmed.ncbi.nlm.nih.gov/25185458/).
FIND (the Foundation of Innovative Diagnostics) in collaboration with WHO has supported the development of target product profile (TPP) for tests predicting TB to identify those in need for preventive therapy (https://www.finddx.org/wp-content/uploads/2019/03/WHO-TPP-predicting-TB-

progression-2017.pdf). The target product profile recommends a positive predictive value (PPV) of >6%. While the PPV might seem low and unambitious, such a test would halve the number needed to treat with preventive treatment compared to the currently used standard of care (tuberculin skin tests and interferon-gamma release assays, IGRAS). The current PPV to predict future TB of IGRAs and TST (2%) which are used to guide preventive therapy fall short of this target product. PPV is driven by prevalence of disease (which is generally very low for TB progression) and specificity. Thus, rather than sensitivity optimising the specificity would be a real step-change. The specificity of TAM-TB in a study conducted among children presenting with symptoms of TB was 97% (https://pubmed.ncbi.nlm.nih.gov/25185458/). Specificity data for TB prediction are currently lacking.

Comment: The tests that detect transcriptomic signatures lack sensitivity. Is the new Xpert test that you are going to use associated with higher sensitivity?

Response: We agree that the tests that rely on characterisations of transcriptomic signatures may lack sensitivity for medium-term prediction of TB (for example as observed in the CORTIS trial). However, performance is better when considered over a shorter interval (47-81% sensitivity over 3 months, https://www.thelancet.com/journals/lanres/article/PIIS2213-2600(19)30282-6/fulltext) and several transcriptomic signatures, including that adapted to the Xpert HR cartridge, have shown good sensitivity and specificity to diagnose TB among people presenting with symptoms (https://www.thelancet.com/journals/lanres/article/PIIS2213-2600(19)30469-2/fulltext) . The sensitivity and specificity of transcriptomic signature-based tests for diagnosis of TB disease in the context of systematic screening is unknown. This is the rationale for the inclusion of these tests in this study.

Comment: Are you going to test protein biomarker signatures in your study? if so, which one in particular?

Response: We will test the stored serum samples for a range of biomarkers (see TBSCREE Chegou 2016, Thorax) including CRP, transthyretin, interferon-gamma, IP-10, apolipoprotein-A1, and serum amyloid A. This is reported in lines 305-307 of the manuscript.

Reviewer #2

• General Comments
Marambire et al present a study protocol for a prospective, observational, longitudinal cohort study of the performance of novel diagnostic and prognostic tests for incipient, preclinical, and active tuberculosis among household TB contacts in three countries in Southern Africa. The expected impact of the study will be to determine the accuracy of novel diagnostics and testing algorithms for early tuberculosis diagnosis and prediction of progression to active TB disease among household contacts

Major Comments

The protocol is well-written and the study is very well-conceived overall. I do have a few important questions and comments outlined below:

Comment: Thank you. Line 67 – The authors cite their plans to recruit highlight infectious cases as a strength, on grounds that it will maximize the number of TB diagnoses in the household cohort. However, recent systematic reviews (i.e., Velen et al ERJ 2021) suggest that the prevalence of TB among household contacts does not differ by index TB patient mycobacterial load. While this is a minor point, this limitation should be considered, and the strengths and limitations of the study design potentially revised.

Response: Thank you for this comment. We disagree with this statement. It is well established that likelihood of infection and secondary TB is higher among contacts of smear positive TB index cases compared to smear negative TB index cases. In fact a recent systematic review showed pool yields for all active TB in household contacts of smear positive TB index cases of 8.30% (3.88-12.73) compared to 2.87% (2.61-3.14) in household contacts of unselected TB index cases (https://bmcinfectdis.biomedcentral.com/articles/10.1186/s12879-021-06609-3).
Smear status is associated with mycobacterial load. People with smear positive TB have higher bacterial loads in their sputum. CT values of the GeneXpert test have been compared to smear status and the same holds true for CT values of molecular tests, lower CT values or semi-quantitative Xpert MTB/RIF grade are associated with higher mycobacterial load and correspond with smear positivity (https://pubmed.ncbi.nlm.nih.gov/28399963/).

Comment: Line 71 – The authors state that this study will provide more precise estimates of the sensitivity of novel diagnostic/prognostic tests compared to population-based cohorts with unknown timing of infection and emphasize this as a strength of the study. I did not see information about how this timing will be determined in the study protocol; could the authors elaborate on how this will be done? How might it inform the analysis in practice, since the timing of infection is usually unknown in routine practice? One other concern is that, given the small number of co-prevalent or incident TB cases with genotypically confirmed household transmission, there may not be sufficient power for stratification of test results by timing of infection.

Response: In population-based cohorts the timing of infection is unknown unless participants have serial tests for M.tb infection. In household contact studies the timing of infection is assumed to be the last infectious contact. However, in countries with high levels of Mtb transmission a person developing TB in a TB affected household might have been infected by a source outside of the household with the timing of infectious contact being unknown. By genotyping both the TB isolate of the index case and the household contact we will be able to distinguish between secondary cases resulting from household transmission (where the timing can be estimated with relative certainty) and outside the household where timing of transmission is unknow. This will reduce the likelihood of misclassification of recent infection.

Comment: Line 115 – The authors state that this study will provide estimates of TB diagnostics for "early TB detection", which they define as "before onward transmission occurs." How can this be confirmed? It seems that this study design may not be able to exclude possible transmission to non-household contacts, since genotyping on these individuals will not be available. If so, the definition of "early TB detection" should be revised.

Response: While the ultimate aim of the study is to validate tests aimed at early TB detection before an individual excretes infectious Mtb bacilli (i.e. before Mtb can be detected microbiologically in a sputum sample), prevention of Mtb transmission is not an outcome of this study. Once tests for earlier TB detection have been validated and their performance is considered good enough to inform TB treatment initiation, trials investigating the effect of these earlier diagnostics on transmission and morbidity are required. This is beyond the scope of this study.
Tests will be validated by investigating serial samples before incident TB has been diagnosed. Investigating stored sputum samples of individuals diagnosed with TB during follow-up will allow to us determine whether or not Mtb was detectable in any sample prior to the TB diagnosis, and describe timing of positive novel tests in relation to sputum positivity.

Comment: Line 251 – The study sample size is powered based on household contacts, with an assumed 3% diagnostic yield. While this is a reasonable estimate of the TB diagnostic yield using

routine clinical or public health definitions, studies of novel diagnostic studies usually require more rigorously defined definitions of cases and controls, to avoid outcome misclassification and underestimates of sensitivity and specificity. With a rigorous gold standard, there may be high rates of indeterminate TB/non-TB outcomes due to various factors including culture contamination, false-positive Xpert Ultra results (especially common in the household context), and difficulty adjudicating microbiologically negative but clinically diagnosed cases of TB. In addition, if the goal is to provide reasonably precise estimates of the sensitivity of novel diagnostics the sample size of 66 TB cases will likely be inadequate, except in the case of extremely sensitive diagnostic assays. Will the authors be able to avoid an underpowered study if there are a lower-than-expected number of confirmed TB cases?

Response: We are unclear what the reviewer means by more rigorously defined definitions of cases and controls. We did not propose to use routine clinical or public health definitions.
We agree that routine clinical definitions of TB which include a diagnosis based on clinical grounds without microbiological confirmation, may result in higher numbers of TB cases compared to one which requires microbiological confirmation. In the recent systematic reviews by Velen et al (ERJ 2021) and Velleca et al (BMC ID 2021) there was a 3.2% and 2.1% prevalence of microbiologically confirmed TB cases among household contacts. Our outcome definition will be determined by an endline review committee and, whilst molecular and culture-based tests will be heavily weighted (including repeated Xpert Ultra and culture for all participants with an initial positive Ultra), other evidence including CXR and clinical presentation will also be considered. As a result, we believe the anticipated TB prevalence among HHC is reasonable. We agree that 64 TB cases may be inadequate for assays with sub-optimal sensitivity. However, the current funding does not allow for a larger sample size or prolonged follow-up. We have applied for additional funding which may facilitate this, and also aim to combine our dataset with data from other studies to improve precision of diagnostic accuracy estimates.

Comment: Lines 279-310, 323-326 – Patients will be enrolled over a two-year period, and some specimens stored for later testing. It should be specified which assays will be performed on stored specimens (information about the several assays that will be performed on fresh specimens has already been provided). For assays performed on bio-banked samples, could the authors state if the stability of the target analyte is known over the relevant storage period and conditions, and, if there are concerns about decay in signal over time, the authors might consider including additional TB-negative controls specifically matched on time in storage.

Response: For tests planned to be done on stored specimens please refer to lines 303-309: "Candidate tests in this category include a serum- or plasma-based multiplex assay assessing 13 protein biomarkers (CRP, procalcitonin[30], sTREM-1[31,32], angiopoietin-2[33,34], interleukin-6[35], TRAIL[36] and IP-10[37]) that is being developed by the London School of Hygiene and Tropical Medicine". This multiplex is currently used on dried blood spots stored for 2 years.
We have not defined all the assays which will be performed on stored samples as this study aims to establish a biorepository for future tests including tests which are currently in development or will be developed in the future. We are unable to comment whether or not the target analytes are stable, however thank you for your suggestion regarding including additional controls matched on time in storage, as well as the matched controls already proposed. Whilst it is not currently planned (and this manuscript reports the current protocol), we will consider this.

Comment: Lines 326-327 – A definition of the reference standard used to calculate sensitivity and specificity should be provided.

Response: A reference has been added.

Comment: Line 333 – The authors mention plans for blinded interpretation of new test results which is a strength; however, other best practices for rigor and reporting of diagnostic studies (e.g. blinding to clinical information, choice and rationale for the reference standard etc.) that are outlined in the STARD reporting guidelines (BMJ. Z015;351:h5527) but not currently described in the study protocol. I suggest that these details be added and a completed STARD checklist submitted as supplementary material.

Response: The STARD checklist has been completed and submitted. This illustrates that we have considered the need to report our study according to STARD guidelines in due course and reported these aspects in the manuscript. However, we feel that the checklist itself is more relevant when presenting results rather than study protocols. We will take the editor’s advice whether or not the checklist should be included.

VERSION 2 – REVIEW

**REVIEWER**	Seas, Carlos<br>Universidad Peruana Cayetano Heredia
**REVIEW RETURNED**	19-Jun-2022

**GENERAL COMMENTS**	I am satisfied by the Author's responses to my enquires

**REVIEWER**	Davis, J. Lucian<br>Yale University, Epidemiology of Microbial Diseases<br><br>I have received funding from the National Institutes of Health for research on TB contact investigation. I declare no other competing interests.
**REVIEW RETURNED**	05-Jun-2022

**GENERAL COMMENTS**	General Comments<br>I am generally satisfied with the author’s response to my queries; I do believe that a more accurate definition of early TB detection would better serve the readers of this study protocol and the TB field.<br><br>Comment Line 67: I defer to the authors on the best inclusion criteria to use for their study, but regarding whether smear-positivity is associated with a higher yield of contact investigation, the Velen et al ERJ 2021 meta-analysis identified 58 studies with data on the yield of contact investigation among contacts of smear-positive index patients and reported a pooled prevalence of 3.7%, almost identical to the 3.6% yield for all TB patients. While the meta-analysis by Velleca et al is also very well done, they only identified 5 studies to contribute to their estimate of prevalence of 8.3% for the yield of contact investigation among smear-positive index patient households; the authors also specifically note that since very few studies reported this on smear-positivity, these estimates may not be accurate. It may not be surprising to find no association, in view of the molecular data from several studies showing that most transmission in high-prevalence countries occurs outside the household.<br><br>Comment Line 71: It’s great that the authors will be doing genotyping to confirm household transmission. However, building on the comment above about household transmission being rare, there

	may not be enough power to estimate the effects of timing of exposure on diagnostic/prognostic performance.<br><br>Comment Line 115 – The author's response confirms the need to revise the definition of early TB detection, since we agree that "before onward transmission occurs" is not measurable, a more accurate operational definition is needed, such as "before patients seek TB diagnostic evaluation." I think most people would say that cases diagnosed though contact investigation or other forms of active case-finding are early compared to cases passively detected after patients present for diagnostic evaluation.<br><br>Comment Line 251- What the authors propose is very reasonable. The interesting situations will be how to classify the asymptomatic smear-positive or Xpert-positive but culture-negative patients with normal chest radiography – cases that seem to be surprisingly common in the preclinical TB cohorts that are now being reported. In a contact investigation prevalence study, these patients would likely be labeled microbiologically confirmed TB, but it would be risky to label them as confirmed TB for a reference standard, since outcome misclassification may have big effects on sensitivity estimates in a small sample.<br><br>Comment Line 279 – Excellent.<br><br>Comment Line 333 – Ok.

VERSION 2 – AUTHOR RESPONSE

Reviewer #1
• I am satisfied by the Author's responses to my enquires

Thank you.

Reviewer #2

• I am generally satisfied with the author's response to my queries; I do believe that a more accurate definition of early TB detection would better serve the readers of this study protocol and the TB field.

Thank you. Please see our response to the definition of early TB detection.

• Comment Line 67: I defer to the authors on the best inclusion criteria to use for their study, but regarding whether smear-positivity is associated with a higher yield of contact investigation, the Velen et al ERJ 2021 meta-analysis identified 58 studies with data on the yield of contact investigation among contacts of smear-positive index patients and reported a pooled prevalence of 3.7%, almost identical to the 3.6% yield for all TB patients. While the meta-analysis by Velleca et al is also very well done, they only identified 5 studies to contribute to their estimate of prevalence of 8.3% for the yield of contact investigation among smear-positive index patient households; the authors also specifically note that since very few studies reported this on smear-positivity, these estimates may not be accurate. It may not be surprising to find no association, in view of the molecular data from several studies showing that most transmission in high-prevalence countries occurs outside the household.

We agree while most of the transmission occurs outside of households, there is a very high risk of transmission within households. For example a systematic review (Martinez et al 2017

https://pubmed.ncbi.nlm.nih.gov/28982226/) estimated that children exposed to TB in the household were 3.79 (95% confidence interval (CI): 3.01, 4.78) times more likely to be infected with Mycobacterium tuberculosis than were their community counterparts. At the population level this only translated into small proportion (<20%) of transmission attributable to household exposure. However, this is not because of the low risk of household transmission, but due to the small number of households with TB index cases.

A person living with somebody with TB has a much higher risk of developing TB than the general population. This is the rational for household contact screening. While there is a pooled TB prevalence (yield) of 3.6% among household contacts (Velen et al, Velleca et al), the TB prevalence found in National TB prevalence surveys is usually much lower.

• Comment Line 71: It's great that the authors will be doing genotyping to confirm household transmission. However, building on the comment above about household transmission being rare, there may not be enough power to estimate the effects of timing of exposure on diagnostic/prognostic performance.

Please see the response to the previous comment. Genotypic and sequencing studies have confirmed that a considerable proportion of secondary TB cases in households result from recent transmission in the household. The pooled estimate of isolate concordance determined by molecular methods (and suggestive of household transmission) across different studies was 70% in low TB incidence setting and 52% in high TB incidence settings. (https://pubmed.ncbi.nlm.nih.gov/28606177/, https://pubmed.ncbi.nlm.nih.gov/16704828/, https://pubmed.ncbi.nlm.nih.gov/22327051/, file:///Users/katharinakranzer/Downloads/Mycobacterium_tuberculosis_infection_in_Southern_A.pdf). While Mtb transmission in households (measure by tuberculin skin tests or interferon gamma release assays) is not infrequent, progression to TB disease in the 2 years following transmission only occurs in 5% of those infected.

• Comment Line 115 – The author's response confirms the need to revise the definition of early TB detection, since we agree that "before onward transmission occurs" is not measurable, a more accurate operational definition is needed, such as "before patients seek TB diagnostic evaluation." I think most people would say that cases diagnosed though contact investigation or other forms of active case-finding are early compared to cases passively detected after patients present for diagnostic evaluation.

Line 115 refers to the abstract. The aim of the study is to validate novel diagnostics which predict TB before a person excretes viable M. tuberculosis in their sputum (i.e. before the person becomes infectious). The study does not aim to identify people before they seek TB diagnostic evaluations. While the study enrolls TB household contacts, it is not about active case finding. The study is a validation study of novel diagnostic tests which are not necessarily sputum based and allow to identify people before M. tuberculosis can be detected in their sputum.

Comment Line 251- What the authors propose is very reasonable. The interesting situations will be how to classify the asymptomatic smear-positive or Xpert-positive but culture-negative patients with normal chest radiography – cases that seem to be surprisingly common in the preclinical TB cohorts that are now being reported. In a contact investigation prevalence study, these patients would likely be labeled microbiologically confirmed TB, but it would be risky to label them as confirmed TB for a reference standard, since outcome misclassification may have big effects on sensitivity estimates in a small sample.

We agree these are interesting cases. As per the protocol only participants with either symptoms or

CXRs suggestive of TB will have a sputum sample investigated. Thus a participant without symptoms and a normal CXR will not undergo any sputum investigations.
